# Hydroboration of Terminal Alkynes Catalyzed by a Mn(I) Alkyl PCP Pincer Complex Following Two Diverging Pathways

**DOI:** 10.1021/acscatal.4c03805

**Published:** 2024-08-05

**Authors:** Daniel P. Zobernig, Berthold Stöger, Luis F. Veiros, Karl Kirchner

**Affiliations:** † Institute of Applied Synthetic Chemistry, TU Wien, Getreidemarkt 9/163-AC, Wien A-1060, Austria; ‡ X-Ray Center, TU Wien, Getreidemarkt 9/163, Wien A-1060, Austria; $ Centro de Química Estrutural, Institute of Molecular Sciences, Departamento de Engenharia Química, Instituto Superior Técnico, Universidade de Lisboa, Av. Rovisco Pais, Lisboa 1049 001, Portugal

**Keywords:** hydroboration, manganese, alkynes, alkyl complex, DFT calculations

## Abstract

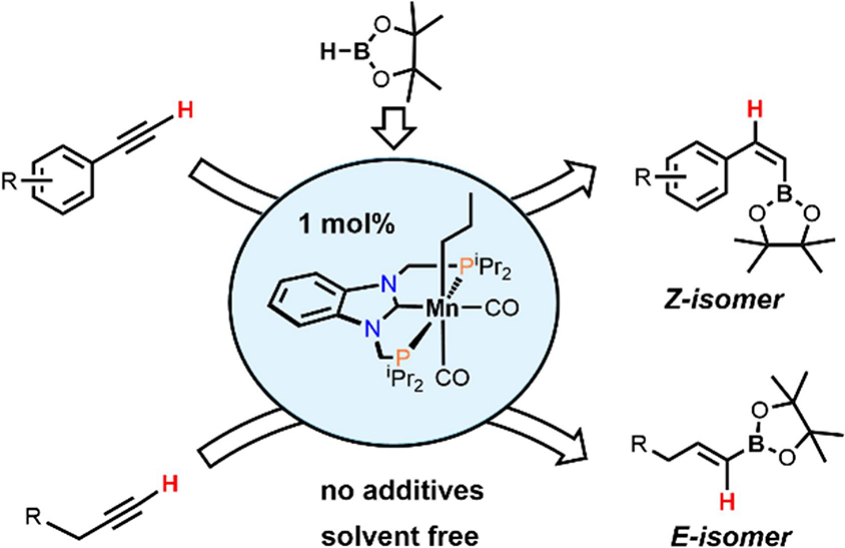

A stereo- and regioselective Mn(I)-catalyzed hydroboration of terminal alkynes with pinacolborane (HBPin) is described. The hydroboration reaction is highly *Z*-selective in the case of aryl alkynes and *E*-selective in the case of aliphatic alkynes. The reaction requires no additives or solvents and proceeds with a catalyst loading of 1 mol % at 50–70 °C. The most active precatalyst is the bench-stable alkyl Mn(I) complex *cis*-[Mn(PCP-*i*Pr)(CO)_2_(CH_2_CH_2_CH_3_)]. The catalytic process is initiated by the migratory insertion of a CO ligand into the M*n*-alkyl bond to yield an acyl intermediate. This species undergoes C–H and B–H bond cleavage of the alkyne (aromatic alkynes) and HBPin (in the case of aliphatic alkynes) forming the active Mn(I) alkynyl and boryl catalysts [Mn(PCP-*i*Pr)(CO)(C≡CR)] and [Mn(PCP-*i*Pr)(CO)(BPin)], respectively. A broad variety of aromatic and aliphatic alkynes was efficiently and selectively borylated. Mechanistic insights are provided based on experimental data and DFT calculations. The functionalized alkenes can be used for further applications in cross-coupling reactions.

## 1 Introduction

**Scheme 1:**
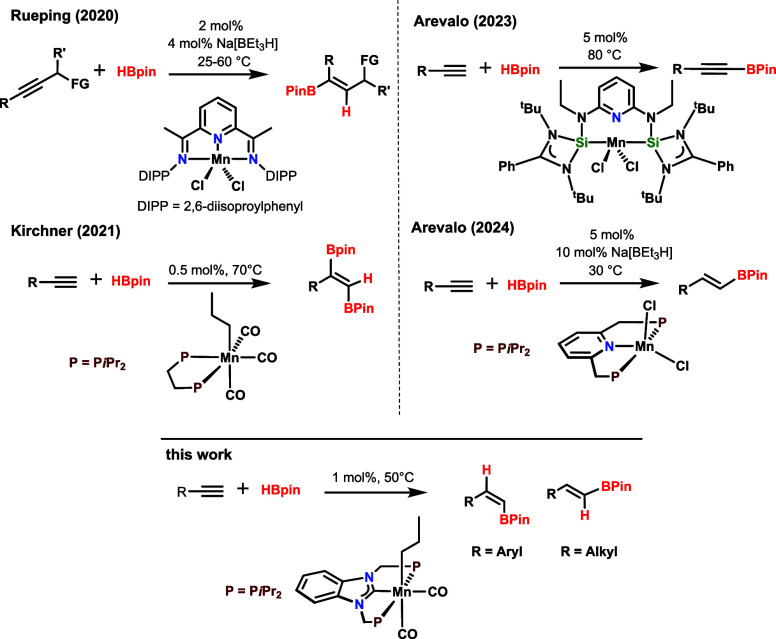
Manganese-Based Catalysts for the Hydroboration of Alkynes (FG = Functional Group)

The use of organoboron reagents especially in the field of cross-coupling chemistry has gained importance within the last few decades, in part due to the emergence of hydroboration catalysts, which make these compounds easily accessible.^1,2^ In this context, the use of dialkoxyboranes such as pinacolborane (HBPin) was introduced due to the stability of HBPin and the hydroborated products.^3^ Transition-metal catalyzed hydroboration of C–C multiple bonds hereby displays a versatile and convenient route toward the aforementioned organoboron species.^4^ Catalysts with noble metals such as Rh^5^ and Ir^6^ are already well researched in the field of C–C multiple bond hydroboration. However, in the last years, nonprecious metal catalysts based on for example Cu,^7^ Ni,^8^ Co,^9^ and Fe^10^ were successfully applied for this reaction. As manganese is concerned, several examples for hydroborations of functional groups such as carbonyls,^11^ nitriles,^12^ CO_2_,^12b,13^ and alkenes^11a,14^ were reported. The first manganese-catalyzed hydroboration of alkynes was reported by Rueping and co-workers in 2020 (Scheme 1).^15^ They reported the hydroboration of symmetrical internal alkynes to yield alkenylboronate esters from the *syn* addition of HBPin and the Mn(II) bis(imino)pyridine complex [Mn(PDI^iPr^)Cl_2_] (PDI^iPr^ = 2,6-(2,6-^i^Pr_2_–C_6_H_3_–N=CMe)_2_C_5_H_3_N) as precatalyst. as precatalyst. An *in situ* activation with Na[HBEt_3_] triggered the catalytic activity of the Mn(II) complex. We described a Mn(I)-catalyzed 1,2-diboration of terminal alkynes with HBPin.^16^ The reaction proceeds with excellent *trans*-1,2-selectivity with no additives required (Scheme 1). The active precatalyst was the bench-stable alkyl bisphosphine Mn(I) complex *fac*-[Mn(dippe) (CO)_3_(CH_2_CH_2_CH_3_)] (dippe = 1,2-bis(di-*iso*-propylphosphino)ethane).^17^ Arevalo et al. showed that the Mn(II) complex [Mn(SiNSi)Cl_2_] (SiNSi = 2,6-[^Et^NSi(N*t*Bu)_2_ CPh]_2_C_5_H_3_N) is an efficient catalyst for the chemoselective C(sp)–H borylation of terminal alkynes (Scheme 1).^18^ Most recently, the same research group reported that the Mn(II) pincer complex [Mn(PNP-*i*Pr)Cl_2_] (PNP-*i*Pr = 2,6-bis(diisopropylphosphinomethyl)pyridine) catalyzed the stereo- and regioselective hydroboration of terminal alkynes by employing HBPin and Na[HBEt_3_] as activator affording exclusively *E*-alkenylboronate esters (Scheme 1).^19^

Here, we describe the activity of *cis*-[Mn(PCP-*i*Pr)(CO)_2_(Br)] (**1**),^20^*cis*-[Mn(PCP-*i*Pr)(CO)_2_(H)] (**2**), *cis*-[Mn(PCP-*i*Pr)(CO)_2_(CH_2_CH_2_CH_3_)] (**3**), and *cis*-[Mn(PCP-*i*Pr)(CO)(κ^2^-H_2_Bpin)] (**4**) as precatalysts for the stereo- and regioselective hydroboration of terminal alkynes. For this reaction, no additives are required. A plausible reaction mechanism based on detailed experimental and theoretical studies is presented.

## 2 Results and Discussion

**Scheme 2:**
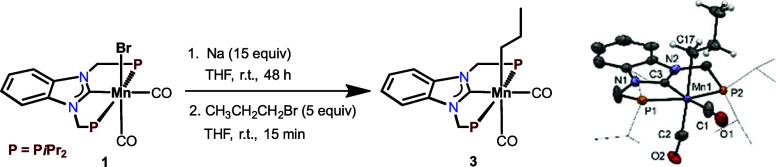
Synthesis of *cis*-[Mn(PCP-*i*Pr)(CO)_2_(CH_2_CH_2_CH_3_)] (**3**) and Structural View of **3** Showing 50% Ellipsoids (Most H Atoms Omitted for Clarity) Selected bond distances (Å) and angles (°): Mn1–C1 1.807(3), Mn1–C2 1.781(3), Mn1–C3 1.952(3), Mn1–C17 2.214(3), Mn1–P1 2.284(1), Mn1–P2 2.284(1), P1–Mn1–P2 160.52(3), C1–Mn1–C3 173.4(1), C2–Mn2–C17 175.8(1).

The alkyl Mn(I) complex *cis*-[Mn(PCP-*i*Pr)(CO)_2_(CH_2_CH_2_CH_3_)] (**3**) was obtained in 57% isolated yield by reacting *cis*-[Mn(PCP-*i*Pr)(CO)_2_(Br)] (**1**) with Na (15 equiv) at room temperature for 48 h and subsequent addition of CH_3_CH_2_CH_2_Br (Scheme 2). This complex is bench-stable for at least 1 week in the presence of air.

**Scheme 3:**
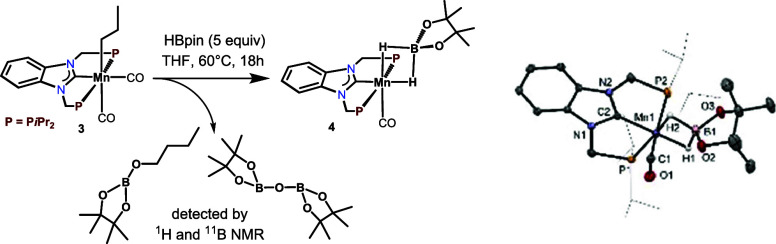
Synthesis of *cis*-[Mn(PCP-*i*Pr)(CO)(κ^2^-H_2_Bpin)] (4) with Structural View of 4 Showing 50% Ellipsoids (Most H Atoms Omitted for Clarity) Selected bond distances (Å) and angles (°): Mn1–C1 1.779(2), Mn1–C2 1.935(2), Mn1–H1 1.60(3), Mn1–H2 1.63(1), Mn1–P1 2.2480(8), Mn1–P2 2.2661(8), P1–Mn1–P2 155.94(3).

Treatment of **3** with HBpin (5 equiv) for 18 h at 60 °C afforded *cis*-[Mn(PCP-*i*Pr)(CO)(κ^2^-H_2_Bpin)] (**4**) in 33% isolated yield (Scheme 3). This reaction was accompanied by the formation of hydroborated butanal (CH_3_CH_2_CH_2_CH_2_OBpin) and (Bpin)_2_O as detected by ^1^H and ^11^B NMR spectroscopy (Scheme 3, see also SI, Figures S3 and S4) and shows that migratory insertion of a CO ligand into the M*n*-alkyl bond to yield an acyl intermediate took place. This complex features an κ^2^-bound H_2_Bpin ligand. While **3** is stable under an inert atmosphere of argon, in solution, this compound starts decomposing within a few minutes. Complexes **3** and **4** were fully characterized by ^1^H, ^11^B{^1^H}, ^13^C{^1^H}, and ^31^P{^1^H} NMR and IR spectroscopy and high-resolution mass spectrometry. In addition, the molecular structures of both complexes were determined by X-ray crystallography. Structural views are depicted in Schemes 2 and 3 with selected bond distances and angles given in the captions.

The catalytic performance of the known Mn(I) complexes **1** and **2** and complexes **3** and **4** was then investigated for the hydroboration of phenylacetylene as a model substrate. Optimization experiments are depicted in Table 1. At 50 °C under solvent-free conditions, complex **1** was catalytically inactive. With complex **2** the corresponding boronic ester **5** was obtained in 41% yield with an *E/Z* ratio of 5/95, thus, being highly *Z*-selective (Table 1, entries 1 and 2). Under the same reaction conditions, complexes **3** and **4** afforded **5** in essentially quantitative yield with *E*/*Z* ratios of 3/97 and 1/99, respectively (Table 1, entries 3 and 4). Due to the higher stability of **3**, we focused in the following on the catalytic activity of **3**. Lowering the catalyst loading to 0.5 mol reduced the yield of **5** to 87% with an *E/Z* ratio of 25/75 (Table 1, entry 5). By using a catalyst loading of 2 mol % at 25 °C, the yield of **5** was significantly reduced to 40%, which was also associated with a poorer *E*/*Z* ratio of 16/84 (Table 1, entry 6). If the catalytic reactions were performed in the solvents THF and toluene with a catalyst loading of 2 mol %, the yields of **5** were 94 and 93%, respectively, with an *E*/*Z* ratio of 1/99. In CH_2_Cl_2_ the yield of **5** dropped to 27% (Table 1, entry 9). Notably, no additives were required to activate either **3** or **4**. In the absence of a catalyst, no conversion of phenylacetylene to **5** was observed (Table 1, entry 10).

**Table 1:**
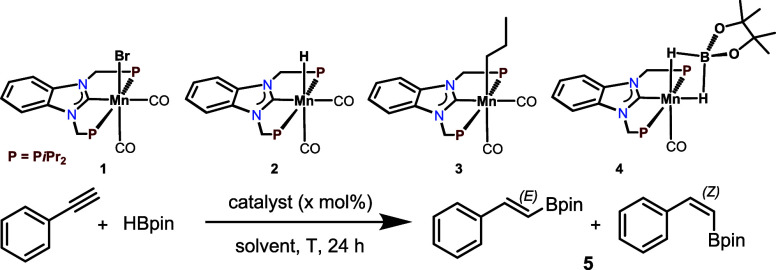
Catalyst Screening and Optimization for the Hydroboration of Phenylacetylene^a^

entry	catalyst (*x* mol %)	solvent	temp. (°C)	yield (%)	*E*/*Z* ratio
1	**1** (1)	neat	50	<1	
2	**2** (1)	neat	50	41	5/95
3	**3** (1)	neat	50	>99	3/97
4	**4** (1)	neat	50	>99	1/99
5	**3** (0.5)	neat	50	87	25/75
6	**3** (2)	neat	25	40	16/84
7	**3** (2)	THF	50	94	1/99
8	**3** (2)	toluene	50	93	1/99
9	**3** (2)	CH_2_Cl_2_	50	27	1/99
10		neat	50	<1	

a Reaction conditions: phenylacetylene (0.25 mmol, 1 equiv), HBpin (0.26 mmol, 1.1 equiv), catalyst (*x* mol %), temp., 24 h, conversion, and *E*/*Z* ratio determined by GC-MS.

Having established the optimal reaction conditions, the scope and limitations were examined (Table 2). In this context, a variety of aromatic and aliphatic alkynes with both electron-withdrawing and electron-donating moieties were tested. Most aromatic substrates react in the presence of 1 mol % catalyst to yield the corresponding alkenylboronate esters with excellent *E*/*Z* ratios up to 1/99 (Table 2, **5a**–**5q**). Likewise, also in the case of 3-ethynylthiophene, trimethylsilylacetylene, and 1-ethynylcyclohexene almost exclusively the *Z*-isomers were formed (Table 2, **5r**–**5t**). In the case of 4-ethynylbenzaldehyde and 1-(4-ethynylphenyl)ethenone) featuring formyl and acyl moieties, respectively, both functional groups were hydroborated as well yielding the respective *Z*-alkenylboronate esters in 77 and 72% isolated yields (Table 2, **5i**, **5j**). *Ortho- and meta*-substituted alkynes were also successfully hydroborated but required higher temperatures to be fully converted (Table 2, **5k**–**5n**). 1,3-Diethynylbenzene was hydroborated to afford **5o** in 75% isolated yield showing that also two triple bonds could be directly converted to the *Z*-alkenylboronate ester (Table 2).

**Table 2:**
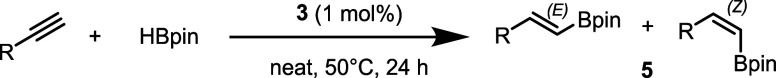

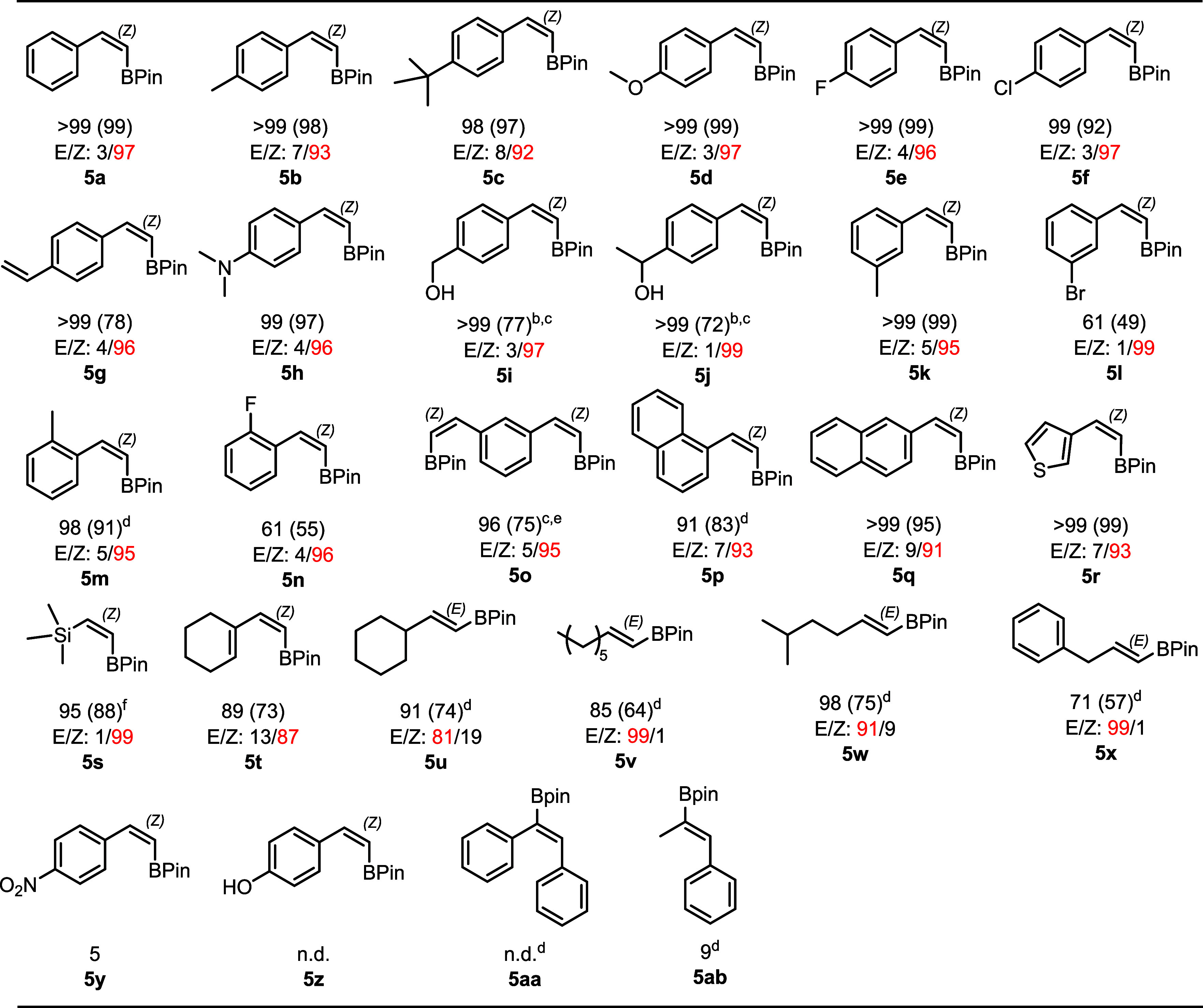
Hydroboration of Various Terminal Alkynes Catalyzed by **3**^a^

a Reaction conditions: alkyne (0.50 mmol, 1 equiv), HBpin (0.55 mmol, 1.1 equiv), **3** (1 mol %), 50 °C, 24 h, conversion determined by GC-MS, *E*/*Z* ratio determined by ^1^H NMR spectroscopy, isolated yield in parentheses.

b **3** (1.5 mol %).

c HBpin (1.05 mmol, 2.1 equiv).

d 70 °C.

e **3** (2 mol %).

f Conversion determined by ^1^H NMR spectroscopy.

Surprisingly, when aliphatic alkynes were hydroborated, the *E*/*Z* ratio was reversed and stereoselectively *E*-alkenylboronate esters were formed in very good yields (Table 2, **5u**–**5x**), but a higher reaction temperature of 70 °C was required. Alkynes bearing nitro and hydroxy groups could not be converted to the alkenylboronate esters **5y** and **5z** (Table 2). Finally, it has to be noted that internal alkynes could not be hydroborated efficiently, with the conversion staying below 10%, even at 70 °C (Table 2, **5aa**, **5ab**).

**Scheme 4:**
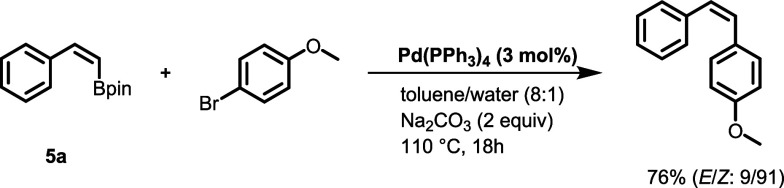
Synthetic Application of the Obtained Vinylboronates

Moreover, we showed that the obtained borylated products can be used as substrates for the stereochemically controlled synthesis of disubstituted olefins. For this purpose, the obtained solution of vinylboronate **5a** was applied without workup in a Suzuki–Miyaura cross-coupling with 4-bromoanisole in the presence of Pd(PPh_3_)_4_ (3 mol %) and Na_2_CO_3_ (2 equiv) at 110 °C for 18 h, which resulted in 76% (*E*/*Z*: 9/91) of product (Scheme 4).

In order to get some mechanistic insights, several experiments were carried out employing phenylacetylene as substrate under standard reaction conditions (Table 2). The homogeneity of the system was proven upon the addition of one drop of Hg which did not lead to a loss of productivity. On the other hand, the addition of 1 equiv of PMe_3_ resulted in only 15% conversion. This indicates that the reaction proceeds via an inner-sphere reaction since PMe_3_ blocks the vacant coordination site of the actual catalyst.

**Scheme 5:**
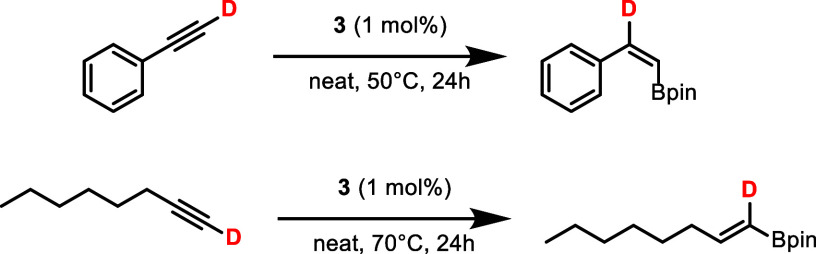
Hydroboration of Deuterated Alkynes Catalyzed by **3** Reaction conditions: alkyne (0.25 mmol, 1 equiv), HBpin (0.28 mmol, 1.1 equiv), **2** (1 mol %), 50–70 °C, 24 h, position of deuterium determined by ^2^H NMR spectroscopy.

Furthermore, in order to gain a deeper understanding of the different stereoselectivities of aromatic and aliphatic alkynes, phenylacetylene-*d*_1_, and octyne-*d*_1_ were used as substrates (Scheme 5). In the case of phenylacetylene-*d*_1_, upon hydroboration, the deuterium ended up exclusively at the benzylic position, indicating that C–D bond cleavage is taking place in the course of the reaction. In contrast, with octyne-*d*_1_, no deuterium migration occurred. These findings reveal that two diverging reaction pathways depending on the acidity of the C–H bond of the alkyne can occur.

The stereo- and regioselective hydroboration of terminal alkynes catalyzed by **3** (**A**^**C**^ in the calculations) was investigated by DFT calculations^21^ using HC≡CPh and HC≡CCH_3_ as model substrates aiming plausible mechanisms that corroborate the experimental results discussed above. The detailed free energy profiles obtained are provided in the SI (Figures S5–S14) while simplified catalytic cycles are depicted in Schemes 6 and 7 with only key intermediates shown.

**Scheme 6:**
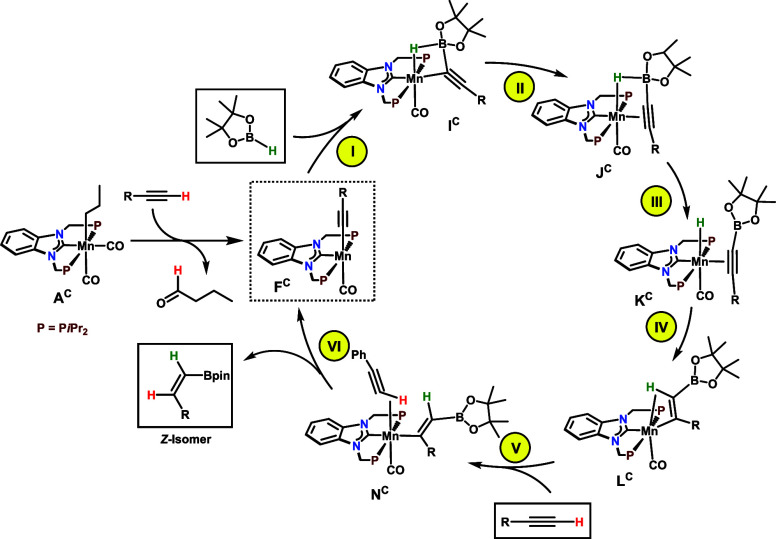
Simplified Catalytic Cycle for the Hydroboration of Terminal Alkynes (Initiation by C–H Activation)

**Scheme 7:**
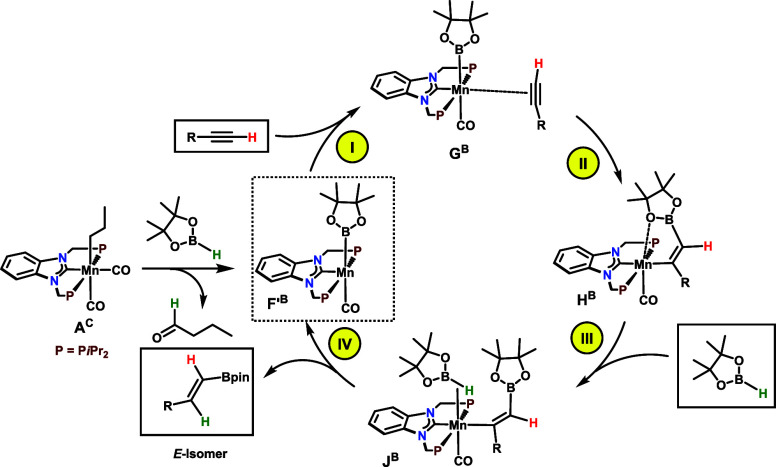
Simplified Catalytic Cycle for the Hydroboration of Terminal Alkynes (Initiation by B–H Activation)

The experimental data clearly suggest that the hydroboration takes place via two different mechanisms depending on the substituents on the carbon–carbon triple bond, i.e., aromatic versus aliphatic groups. It has to be noted that the acidity of the terminal C–H bond of aromatic and aliphatic alkynes is different (p*K*_a_ (aromatic) ≈ 23, p*K*_a_ (aliphatic) ≈ 25).^22^ Accordingly, the order of C–H and B–H bond activation steps of alkyne and HBpin, respectively, in the catalytic cycles may be the decisive factor as far as selectivity control is concerned.

For the formation of *Z*-alkenylboronate esters from aromatic alkynes, catalyst initiation starts with the migratory insertion of the propyl ligand into a Mn–CO bond, in **A**^**C**^, to form an acyl species stabilized by an agostic C–H bond. This was reported previously for *fac*-[Mn(dippe)(CO)_3_(CH_2_CH_2_CH_3_)].^23^ Addition of HC≡CPh followed by activation of the terminal C–H bond gives the 16e alkynyl catalyst [Mn(PCP-*i*Pr)(CO)(C≡CPh)] (**F**^**C**^) together with liberated butanal (hydroborated under these conditions) (see SI Figure S5). The highest barrier for the C–H bond activation and cleavage process is 27.8 kcal/mol, corresponding to HC≡CPh coordination (**TS**^**C**^_**CD**_, in Figure S5). For comparison, the equivalent barrier for the same process with HC≡CCH_3_ is somewhat higher, Δ*G*^‡^ = 30.8 kcal/mol, in agreement with the less acidic C–H bond compared to HC≡CPh (see SI Figure S6).

The addition of HBPin to **F**^**C**^ results in the formation of intermediate **I**^**C**^ where both new B–C and Mn–H bonds are formed, while the B–H bond remains almost intact. An η^1^ to η^2^ rearrangement of the alkyne moiety leads to **J**^**C**^. This corresponds to steps **I** and **II** in the cycle of Scheme 6 (see SI, Figure S7). The highest barrier along the path is Δ*G*^‡^ = 8.7 kcal/mol measured from intermediate **F**^**C**^ to **TS**^**C**^_**HI**_ corresponding to the addition of HBPin to the metallic moiety. The reaction from **J**^**C**^ to **K**^**C**^ proceeds with B–H bond cleavage forming a metal-hydride and an η^2^-coordinated alkyne in a facile process requiring merely 2.9 kcal/mol (**TS**^**C**^_**JK**_ in Figure S8). Insertion of the C–C triple bond into the Mn–H bond of **K**^**C**^ affords the vinylboryl species **L**^**C**^ featuring a stabilizing agostic C–H bond. The addition of another HC≡CPh molecule to **L**^**C**^ leads to intermediate **M**^**C**^ and to the final step in the mechanism with protonation of the vinylboryl ligand and release of the final product, the respective *Z*-alkenylboronate ester. The alkynyl ligand is regenerated to form **O**^**C**^. The barrier associated with this process from **M**^**C**^ via the alkyne complex **N**^**C**^ to the alkynyl complex with the *Z*-alkenylboronate ester **O**^**C**^ is Δ*G*^‡^ = 12.2 kcal/mol (**TS**^**C**^_**NO**_), and the step is clearly favorable from the thermodynamic point of view with Δ*G* = −13.8 kcal/mol (corresponding to steps **V** and **VI** in the cycle of Scheme 6, see SI Figure S8). Liberation of the coordinated *Z*-alkenylboronate ester closes the catalytic cycle reforming thereby **F**^**C**^, with a favorable free energy balance of Δ*G* = −1.2 kcal/mol and an overall barrier for the catalytic cycle of Δ*G*^‡^ = 12.2 kcal/mol, measured from the vinyl intermediate with HC≡CPh, **M**^**C**^, to the transition state **TS**^**C**^_**NO**_ for vinyl protonation and product formation.

The formation of *Z*-alkenylboronate esters is kinetically controlled (see the SI, Figures S8 and S10). The product forming step via **TS**^**C**^_**NO**_ to give **O**^**C**^ is 4.2 kcal/mol more favorable than the respective process to afford *E*-alkenylboronate ester **U**^**C**^ via **TS**^**C**^_**TU**_ (Figures S9 and S10).

As far as the formation of *E*-alkenylboronate esters from aliphatic alkynes is concerned, catalyst initiation also starts subsequent to the migratory insertion of the propyl ligand into a Mn–CO bond **A**^**C**^ to afford **B**^**C**^ (see SI, Figure S11). The addition of HBPin to **B**^**C**^ leads, via intermediate **C**^**B**^, to the formation of acyl species **D^B^**, which subsequently, upon rotation of the acyl moiety about the Mn–C bond by ca. 80°, affords **E**^**B**^. Both **D**^**B**^ and **E**^**B**^ contain a κ^2^-*B,H*-bound HBpin ligand. In **E**^**B**^, the HBpin ligand undergoes B–H bond cleavage accompanied by protonation of the acyl moiety to afford the boryl catalyst [Mn(PCP-*i*Pr)(CO)(Bpin)] (**F**^**B**^ as butanal adduct) that, upon addition of HC≡CCH_3_ yields **G**^**B**^ together with liberated butanal. The overall barrier for these steps, i.e., alkyl migration and B–H bond activation and cleavage, is 26.7 kcal/mol measured from the free reactants, HBPin and **A**^**C**^, to **TS**^**B**^_**AB**_, the transition state for HBPin κ^2^-coordination. For comparison, C–H bond activation of HC≡CCH_3_ to form the putative alkynyl species [Mn(PCP-*i*Pr)(CO)(C≡CCH_3_)] requires a barrier of 30.8 kcal/mol, which is 6.6 kcal/mol higher than the barrier for the formation of **F**^**B**^ via H–B bond cleavage (see SI Figures S6 and S11). Facile insertion of HC≡CCH_3_ into the Mn–B bond affords the vinylboryl intermediate **H**^**B**^ (**II** in Scheme 7) in an almost barrierless (Δ*G*^‡^ = 0.3 kcal/mol) and clearly favorable step (Δ*G* = −44.1 kcal/mol). The addition of HBpin to **H**^**B**^ leads to **I**^**B**^ and, then, to **J**^**B**^, a B–H κ^2^-complex from which protonation of the vinylboryl ligand forms intermediate **K**^**B**^ with loosely bound *E*-alkenylboronate ester, the final product (see Figure S12). The previous process from **H**^**B**^ to **K**^**B**^ corresponds to steps **III** and **IV** in the cycle of Scheme 7. It is practically thermoneutral (Δ*G* = 0.5 kcal/mol) and has a barrier of Δ*G*^‡^ = 22.7 kcal/mol (measured from **H**^**B**^ to **TS**^**B**^_**JK**_) that is also the overall barrier of the catalytic cycle. Closing the cycle with the liberation of the product, the *E*-alkenylboronate ester, and regenerating **G**^**B**^, the boryl intermediate (plus a HC≡CCH_3_ molecule) has a free energy balance of 5.6 kcal/mol.

It has to be noted that the respective *Z*-isomer may be easily formed upon isomerization starting from **H**^**B**^ (see SI, Figures S13 and S14). Gratifyingly, in agreement with the experimental results, the *E*-isomer is more stable than the *Z*-isomer by 2.7 kcal/mol. Accordingly, the formation of the *E*-isomer is thermodynamically controlled, rather than kinetically controlled.

## 3 Conclusions

In conclusion, the bench-stable alkyl Mn(I) complex *fac-*[Mn(PCP-*i*Pr)(CO)_2_(CH_2_CH_2_CH_3_)] turned out to be an efficient catalyst for the additive-free stereo- and regioselective hydroboration of terminal alkynes with HBPin. Hydroboration takes place with a catalyst loading of 1 mol % at 50–70 °C with a high *Z*-selectivity in the case of aryl alkynes and essentially *E*-selectivity in the case of aliphatic alkynes. The catalytic process is initiated by migratory insertion of a CO ligand into the M*n*-alkyl bond to yield an acyl intermediate, which undergoes C–H activation of the terminal alkyne in the case of aromatic alkynes and B–H bond cleavage of HBPin for aliphatic alkynes. Thereby, the catalytically active 16e^–^ Mn(I) alkynyl and boryl species [Mn(PCP-*i*Pr)(CO)(C≡CR)] and [Mn(PCP-*i*Pr)(CO)(BPin)], respectively, are formed. A broad variety of aromatic and aliphatic alkynes were efficiently and selectively borylated. Mechanistic insights are provided based on experimental and computational studies. The functionalized alkenes can be used for further applications, which have been demonstrated for a Suzuki–Miyaura cross-coupling reaction.

## Supporting Information Available

Synthetic procedures, ^1^H, ^13^C{^1^H}, and ^31^P{H} NMR spectra of all compounds, crystallographic data, and complete computational details (PDF)
X-ray crystallographic data for **3** and **4** (CCDC 2308703 and 2308704) (CIF)
Cartesian coordinates for DFT-optimized structures (XYZ)

## Supplementary Material

cs4c03805_si_001.pdf

cs4c03805_si_002.cif

cs4c03805_si_003.xyz
