# Supplementary material for: Hydroboration of Terminal Alkynes Catalyzed by a Mn(I) Alkyl PCP Pincer Complex Following Two Diverging Pathways
Source: ACS Catal. 2024 Aug 5;14(16):12385–91. doi: 10.1021/acscatal.4c03805 (PMC11334104; doi:10.1021/acscatal.4c03805)
Supplement: Supplementary file 1 — cs4c03805_si_001.pdf [file cs4c03805_si_001.pdf]

Hydroboration of Terminal Alkynes Catalyzed by a Mn(I) Alkyl PCP Pincer Complex following Two Diverging Pathways

Daniel P. Zobernig,[†] Berthold Stöger,[‡] Luis F. Veiros,[$] Karl Kirchner*,[†]

[†] Institute of Applied Synthetic Chemistry, TU Wien, Getreidemarkt 9/163-AC, A-1060 Wien, Austria.
[‡] X-Ray Center, TU Wien, Getreidemarkt 9/163, A-1060 Wien, Austria.
[$] Centro de Química Estrutural, Institute of Molecular Sciences, Departamento de Engenharia Química, Instituto Superior Técnico, Universidade de Lisboa, Av. Rovisco Pais, 1049 001 Lisboa, Portugal

E-mail: karl.kirchner@tuwien.ac.at

Supporting Information

Experimental Section 2
General Information 2
General Procedure for Hydroboration of Alkynes 2
Syntheses 2
Substrate Synthesis 3
Mechanistic Studies 3
Poisoning with Mercury 3
Poisoning with Trimethylphosphine 3
Deuteration Experiment with Phenylacetylene-d_1 3
Deuteration Experiment with 1-Octyne-d_1 4
Control Experiment with *cis*-[Mn(PCP-*i*Pr)$(CO)_2$Br] (**1**) 4
Control Experiment with *cis*-[Mn(PCP-*i*Pr)$(CO)_2$H] (**2**) 4
Control Experiment with *cis*-[Mn(PCP-*i*Pr(CO)(κ^2-H_2Bpin) (**4**) 4
Hydroboration of Phenylacetylene and Subsequent Suzuki-Miyaura Cross-Coupling with Bromanisole 5
Migratory Insertion and Formation of complex **4**. 5
X-ray Structure Determination. 6
Computational details 6
Characterization of Organic Products 12
References 17
NMR and IR Spectra 19
Complexes 19
Organic Compounds 24

Experimental Section

General Information. All reactions were performed under an inert atmosphere of argon by using Schlenk techniques or in a MBraun inert-gas glovebox. The solvents were purified according to standard procedures. All alkyne-substrates were purchased from Sigma-Aldrich, Acros Organics, TCI or BLDpharm and used as purchased without further purification. The deuterated solvents were purchased from Eurisotope and dried over 3 Å molecular sieves. ^{1}H, $^{11}B\{^{1}H\}$, $^{13}C\{^{1}H\}$ and $^{31}P\{^{1}H\}$ NMR spectra were recorded on Bruker AVANCE-400 and AVANCE-600 spectrometers. ^{1}H and $^{13}C\{^{1}H\}$ NMR spectra were referenced internally to residual protio-solvent, and solvent resonances, respectively, and are reported relative to tetramethylsilane (δ = 0 ppm). $^{11}B\{^{1}H\}$ NMR spectra were referenced externally to $BF_3.OEt_2$ in $CDCl_3$ (δ = 0 ppm). $^{31}P\{^{1}H\}$ NMR spectra were referenced externally to H_3PO_4 (85%) (δ = 0 ppm). Preparative flash column chromatography was conducted manually using glass columns packed with silica gel 60 (Merck, 40-63 μm).
Complexes *cis*-[Mn(PCP-*i*Pr)$(CO)_2$(Br)] (**1**) and *cis*-[Mn(PCP-*i*Pr)$(CO)_2$(H)] (**2**) were synthesiszed according to literature.[1] Phenylacetylene-d_1 (>98 % D) was synthesized from phenylacetylene, *n*-BuLi and D_2O. 1-Octyne-d_1 (>98% D) was synthesized from 1-octyne, *n*-Buli and D_2O.

High resolution-accurate mass data mass spectra were recorded on a hybrid Maxis Qq-aoTOF mass spectrometer (Bruker Daltonics, Bremen, Germany) fitted with an ESI-source. Measured accurate mass data of the $[M]^+$ ions for confirming calculated elemental compositions were typically within ±5 ppm accuracy. The mass calibration was done with a commercial mixture of perfluorinated trialkyl-triazines (ES Tuning Mix, Agilent Technologies, Santa Clara, CA, USA).

GC–MS analyses were conducted on a ISQ LT Single quadrupole MS (Thermo Fisher) directly interfaced to a TRACE 1300 Gas Chromatographic systems (Thermo Fisher), using a Rxi-5Sil MS (30 m, 0.25mm ID) cross-bonded dimethyl polysiloxane capillary column at a carrier flow of He 1.5 mL/min.

General Procedure for Hydroboration of Alkynes. Inside an argon flushed glovebox, a screw cap vial (8 mL) was charged with **3** (1 - 2 mol%), alkyne (0.5 mmol, 1.0 equiv.) and pinacolborane (0.55 – 1.05 mmol, 1.1 – 2.1 equiv.) in this order. A stirring-bar was added, the vial was closed, transferred out of the glovebox and was stirred for 24 hours at 50°C if not stated otherwise. Afterwards the reaction mixture was allowed to reach room temperature and was exposed to air to quench the catalyst. Diethyl ether (1 mL) was added and 2 μL of the sample was analyzed *via* GC-MS. To the remaining solution was added water (3 mL) and the mixture was stirred for 2 hours at room temperature. The phases were subsequently separated, the aqueous phases extracted with diethyl ether (2x2 mL) and the organic phases combined. The resulting solution was filtered through a thin pad of *silica*, after which the solvent was carefully removed, yielding the pure product. It was characterized with ^{1}H- and $^{13}C\{^{1}H\}$ NMR spectroscopy. If further purification was necessary, the product was purified *via* column chromatography.

Syntheses. *cis*-[Mn(PCP-*i*Pr)$(CO)_2(CH_2CH_2CH_3)$] (3). **1** (330 mg, 0.580 mmol, 1 equiv.) was dissolved in dry THF (15 mL) under an argon atmosphere and sodium (200 mg, 8.70 mmol, 15 equiv.) was added in one portion. The resulting mixture was stirred at room temperature for 48 hours, after which 1-bromopropane (710 mg, 5.80 mmol, 10 equiv.) was added, stirring for another 15 minutes. The solution was then decanted off, the precipitate was extracted with diethylether (2x10 mL) and the combined organic phases were taken to dryness. The solid was thereafter extracted with *n*-pentane (7x20 mL), and the solvent was removed in vacuo. The resulting orange powder was washed with cold *n*-pentane (10 mL) and dried, yielding 175 mg of **3** (57%) as a beige solid. ^{1}H NMR (600 MHz, C_6D_6): δ = 7.00 – 6.96 (m, 2H, aromatic H), 6.73 – 6.67 (m, 2H, aromatic H), 3.69 (ddt, *J* = 65.1, 12.6, 2.2 Hz, 4H, N-CH_2-P), 2.40 – 2.28 (m, 2H, P-$CH(CH_3)_2$), 1.48 (q, *J* = 7.4 Hz, 6H, P-$CH(CH_3)_2$), 1.41 – 1.37 (m, 8H, P-$CH(CH_3)_2$, superimposed by P-$CH(CH_3)_2$), 1.31 – 1.25 (m, 8H P-$CH(CH_3)_2$, superimposed by Mn-CH_2-CH_2-CH_3), 1.11 (t, *J* = 7.0 Hz, 3H, Mn-CH_2-CH_2-CH_3), 1.01 (td, *J* = 7.0, 4.5 Hz, 6H, P-$CH(CH_3)_2$), 0.07 – -0.02 (m, 2H, Mn-CH_2-CH_2-CH_3) ppm. $^{31}P\{^{1}H\}$ NMR (243 MHz, C_6D_6): δ = 118.1 ppm. $^{13}C\{^{1}H\}$ NMR (151 MHz, C_6D_6): δ = 236.5 (CO), 234.0 (CO), 228.3 (N-C-N), 136.1 (aromatic C), 121.8 (aromatic CH), 109.5 (aromatic CH), 48.3 (N-CH_2-P), 30.5 (P-$CH(CH_3)_2$), 30.2 (P-$CH(CH_3)_2$), 29.7 (Mn-CH_2-CH_2-CH_3), 27.0 (t, J = 8.5 Hz, P-$CH(CH_3)_2$), 26.5 (t, J = 5.3 Hz, P-$CH(CH_3)_2$), 23.0 (Mn-CH_2-CH_2-CH_3), 20.4 (P-$CH(CH_3)_2$), 20.0 (P-$CH(CH_3)_2$), 19.3 (P-$CH(CH_3)_2$), 18.8 (P-$CH(CH_3)_2$), 11.3 (t, J = 16.7 Hz, Mn-CH_2-CH_2-CH_3) ppm. FTIR (cm^{-1}): 1881 (CO), 1808 (CO). HR-MS: *m*/*z* calcd for $C_{26}H_{43}MnN_2O_2P_2$ $[M]^+$ 532.2174, found 532.2162.

[Mn(PCP-*i*Pr)(CO)(κ^2-H$_2$Bpin)] (4). **3** (50 mg, 0.094 mmol) was dissolved in dry THF (1 mL) under an argon atmosphere and pinacolborane (68 µL, 0.47 mmol, 5 equiv.) was added. The solution was heated to 60°C for 1 day, after which all solvent was removed in *vacuo*. The resulting residue was washed with *n*-pentane (2x3 mL) and finally extracted with *n*-pentane (3x8 mL), yielding 18 mg (33%) of **4** as a yellow solid. ^{1}H NMR (400 MHz, C_6D_6): δ = 7.05 – 6.97 (m, 2H, aromatic H), 6.82 – 6.71 (m, 2H, aromatic H), 3.78 (dd, *J* = 12.9, 6.2 Hz, 2H, P-CH$_2$-N), 3.36 (d, *J* = 12.9 Hz, 2H, P-CH$_2$-N), 3.03 – 2.88 (m, 2H, P-CH(CH$_3$)$_2$), 2.36 – 2.19 (m, 2H, P-CH(CH$_3$)$_2$), 1.50 – 1.30 (m, 12H, P-CH(CH$_3$)$_2$), 1.26 (s, 6H, O-C(CH$_3$)$_2$), 1.19 (s, 6H, O-C(CH$_3$)$_2$), 1.16 – 0.94 (m, 12H, P-CH(CH$_3$)$_2$) -9.70 (bs, 1H, BH$_2$), -11.13 (bs, 1H, BH$_2$) ppm. ^{11}B{^{1}H} NMR (193 MHz, C_6D_6): δ = 30.3 ppm. ^{31}P{^{1}H} NMR (243 MHz, C_6D_6): δ = 120.4 ppm. ^{13}C{^{1}H} NMR (151 MHz, C_6D_6): δ = 234.8 (CO), 136.6 (aromatic C), 121.4 (aromatic CH), 108.7 (aromatic CH), 80.9 (O-C(CH$_3$)$_2$), 43.0 – 42.8 (m, P-CH$_2$-N), 27.1 (t, *J* = 8.2 Hz, P-CH(CH$_3$)$_2$), 26.0 (t, J = 6.4 Hz, P-CH(CH$_3$)$_2$), 24.9 (d, *J* = 5.4 Hz, O-C(CH$_3$)$_2$), 19.8 (P-CH(CH$_3$)$_2$), 19.3 (P-CH(CH$_3$)$_2$), 18.9 (P-CH(CH$_3$)$_2$), 17.6 (P-CH(CH$_3$)$_2$) ppm (N-C-N could not be detected). FTIR (cm^{-1}): 1824 (CO). HR-MS: *m/z* calcd for $C_{22}H_{37}MnN_2OP_2$ [M-$C_6H_{14}BO_2$]$^+$ 461.1677, found 461.1685.

Substrate Synthesis. 4-Ethynylstyrene. This compound was synthesized according to literature.[2] 4-Bromostyrene (1.8 g, 10.0 mmol, 1.0 equiv.), trimethylamine (20 mL), trimethylsilylacetylene (2.26 g, 23 mmol, 2.3 equiv.) and PdCl$_2$(PPh$_3$)$_2$ (280 mg, 0.4 mmol, 4 mol%) were combined in a Schlenk flask under inert atmosphere and heated to 50 °C for 5 minutes, after which CuI (57 mg, 0.3 mmol, 3 mol%) was added. The resulting mixture was stirred at 50 °C for 24 hours and was subsequently quenched with a saturated NH_4Cl solution (20 mL). The suspension was extracted with diethyl ether (2x20 mL), the organic phases were combined and washed with water (10 mL) and brine (10 mL) and dried over anhydrous Na_2SO_4. The solvent was removed in *vacuo*, after which methanol (20 mL) and K_2CO_3 (2.76 g, 20 mmol, 2.0 equiv.) were added and the mixture was stirred for 24 hours at room temperature. A saturated NH_4Cl solution (10 mL) was subsequently added, the resulting suspension extracted with diethyl ether (2x15 mL), the organic phases combined, washed with water (10 mL) and brine (10 mL) and dried over anhydrous Na_2SO_4. After removing the solvent under reduced the pressure, the crude product was purified *via* column chromatography (eluent: petroleum ether), affording 200 mg (16%) of 4-ethynylstyrene as a colourless oil. ^{1}H NMR (400 MHz, $CDCl_3$): δ = 7.50 – 7.42 (m, 2H), 7.41 – 7.32 (m, 2H), 6.70 (dd, *J* = 17.6, 10.9 Hz, 1H), 5.77 (d, *J* = 17.6 Hz, 1H), 5.31 (d, *J* = 10.9 Hz, 1H), 3.11 (s, 1H) ppm. ^{13}C{^{1}H} NMR (101 MHz, $CDCl_3$): δ = 138.1, 136.3, 132.5, 126.2, 121.5, 115.2, 83.8, 77.9 ppm.

Mechanistic Studies

Poisoning with Mercury. Inside an argon flushed glovebox, a screw cap vial (8 mL) was charged with **3** (1.3 mg, 2.5 µmol, 1 mol%), phenylacetylene (28 µL, 0.25 mmol, 1 equiv.), pinacolborane (40 µL, 0.27 mmol, 1.1 equiv.) and one drop of mercury in this order. A stirring-bar was added, the vial was closed, transferred out of the glovebox and was stirred for 24 hours at 50°C. Afterwards the reaction mixture was allowed to reach room temperature and was exposed to air to quench the catalyst. Diethyl ether (1 mL) was added and 2 µL of the sample was analyzed *via* GC-MS. A conversion of 96% was observed.

Poisoning with Trimethylphosphine. Inside an argon flushed glovebox, a screw cap vial (8 mL) was charged with **3** (1.3 mg, 2.5 µmol, 1 mol%), phenylacetylene (28 µL, 0.25 mmol, 1.0 equiv.), pinacolborane (40 µL, 0.27 mmol, 1.1 equiv.) and trimethylphosphine (26 µL, 0.25 mmol, 1.0 equiv.) in this order. A stirring-bar was added, the vial was closed, transferred out of the glovebox and was stirred for 24 hours at 50°C. Afterwards the reaction mixture was allowed to reach room temperature and was exposed to air to quench the catalyst. Diethyl ether (1 mL) was added and 2 µL of the sample was analyzed *via* GC-MS. A conversion of only 15% was observed.

Deuteration Experiment with Phenylacetylene-d$_1$. Inside an argon flushed glovebox, a screw cap vial (8 mL) was charged with **3** (1.3 mg, 2.5 µmol, 1 mol%), phenylacetylene-d$_1$ (28 µL, 0.25 mmol, 1 equiv.) and pinacolborane (40 µL, 0.27 mmol, 1.1 equiv.) in this order. A stirring-bar was added, the vial was closed, transferred out of the glovebox and was stirred for 24 hours at 50°C. Afterwards the reaction mixture was allowed to reach room temperature and was exposed to air to quench the catalyst. Benzene (0.7 mL) was added and analyzed *via* ^{2}H-NMR spectroscopy. The deuterium could exclusively be found in the benzylic position.

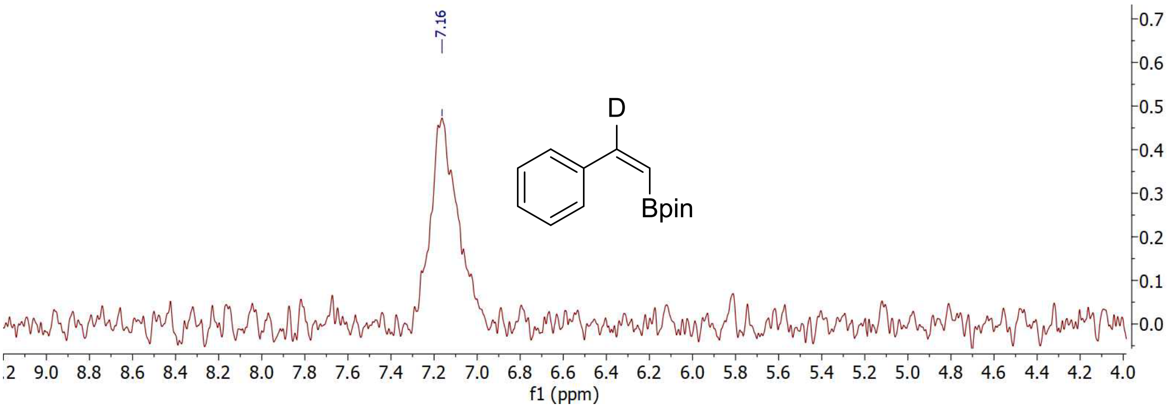

Figure S1. ^{2}H-NMR spectrum of phenylacetylene-d$_1$ after hydroboration reaction

Deuteration Experiment with 1-Octyne-d$_1$. Inside an argon flushed glovebox, a screw cap vial (8 mL) was charged with **3** (1.3 mg, 2.5 µmol, 1 mol%), 1-octyne-d$_1$ (37 µL, 0.25 mmol, 1 equiv.) and pinacolborane (40 µL, 0.27 mmol, 1.1 equiv.) in this order. A stirring-bar was added, the vial was closed, transferred out of the glovebox and was stirred for 24 hours at 70°C. Afterwards the reaction mixture was allowed to reach room temperature and was exposed to air to quench the catalyst. Benzene (0.7 mL) was added and analyzed via 2H-NMR spectroscopy. The deuterium could exclusively be found in the terminal position.

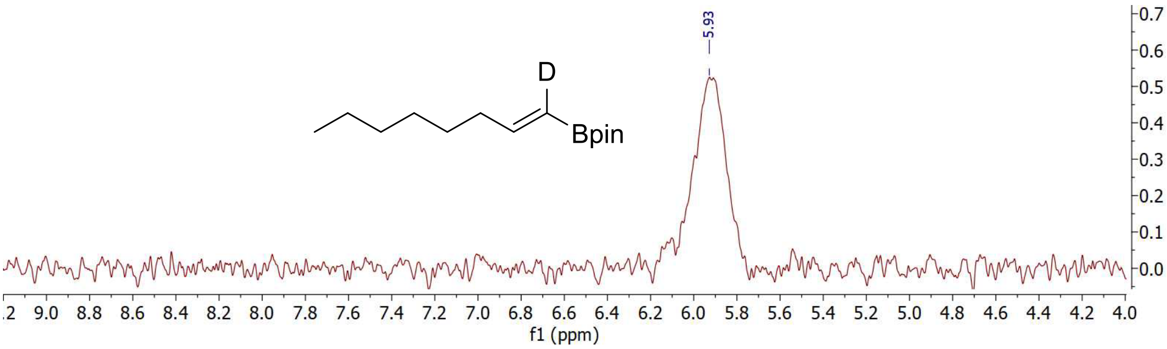

Figure S2. ^{2}H-NMR spectrum of 1-octyne-d$_1$ after hydroboration reaction

Control Experiment with *cis*-[Mn(PCP-*i*Pr)(CO)$_2$(Br)] (1). Inside an argon flushed glovebox, a screw cap vial (8 mL) was charged with **1** (1.3 mg, 2.5 µmol, 1.0 mol%), phenylacetylene (28 µL, 0.25 mmol, 1.0 equiv.) and pinacolborane (40 µL, 0.27 mmol, 1.1 equiv.). A stirring-bar was added, the vial was closed, transferred out of the glovebox and was stirred for 24 hours at 50°C. Afterwards the reaction mixture was allowed to reach room temperature and was exposed to air to quench the catalyst. Diethyl ether (1 mL) was added and 2 µL of the sample was analyzed *via* GC-MS. A conversion of less than 1% was observed.

Control Experiment with *cis*-[Mn(PCP-*i*Pr)(CO)$_2$(H)] (2). Inside an argon flushed glovebox, a screw cap vial (8 mL) was charged with **2** (1.2 mg, 2.5 µmol, 1.0 mol%), phenylacetylene (28 µL, 0.25 mmol, 1.0 equiv.) and pinacolborane (40 µL, 0.27 mmol, 1.1 equiv.) in this order. A stirring-bar was added, the vial was closed, transferred out of the glovebox and was stirred for 24 hours at 50°C. Afterwards the reaction mixture was allowed to reach room temperature and was exposed to air to quench the catalyst. Diethyl ether (1 mL) was added and 2 µL of the sample was analyzed *via* GC-MS. A conversion of 35 % was observed.

Control Experiment with *cis*-[Mn(PCP-*i*Pr)(CO)(κ^2-H$_2$Bpin)] (4). Inside an argon flushed glovebox, a screw cap vial (8 mL) was charged with **4** (1.5 mg, 2.5 µmol, 1.0 mol%), phenylacetylene (28 µL, 0.25 mmol, 1.0 equiv.) and pinacolborane (40 µL, 0.27 mmol, 1.1 equiv.) in this order. A stirring-bar was added, the vial was closed, transferred out of the glovebox and was stirred for 24 hours at 50°C. Afterwards the reaction mixture was allowed to reach room temperature and was exposed to air to quench the catalyst. Diethyl ether (1 mL) was added and 2 µL of the sample was analyzed *via* GC-MS. A conversion of 99% was observed.

Hydroboration of Phenylacetylene and Subsequent Suzuki-Miyaura Cross-Coupling with Bromanisole. The reaction was conducted using a modified procedure from literature.[3] Inside an argon flushed glovebox, a screw cap vial (8 mL) was charged with **3** (2.6 mg, 0.5 µmol, 1 mol%), phenylacetylene (55 µL, 0.5 mmol, 1 equiv.) and pinacolborane (80 µL, 0.55 mmol, 1.1 equiv.) in this order. A stirring-bar was added, the vial was closed, transferred out of the glovebox and was stirred for 24 hours at 50°C. Afterwards the reaction mixture was allowed to reach room temperature and was exposed to air to quench the catalyst for 10 min. Tetrakis(triphenylphosphine)palladium(0) (17.3 mg, 1.5 µmol, 3 mol%), Na_2CO_3 (106 mg, 1 mmol, 2 equiv.), 4-bromoanisole (63 µL, 0.5 mmol, 1 equiv.), toluene (4 mL) and water (0.5 mL) were added to the reaction and the mixture was stirred at 110°C for 18 hours. The reaction was left to cool and 6µL of the organic phase was analyzed *via* GC-MS. A conversion of 76% (*E*/*Z*: 9/91) was detected.

Migratory Insertion and Formation of complex 4. 3 (10 mg, 0.019 mmol) was dissolved in dry THF-d_8 (0.1 mL) under an argon atmosphere and pinacolborane (14 µL, 0.094 mmol, 5 equiv.) was added. The solution was heated to 60°C for 18 hours, after which more THF-d_8 (0.5 mL) was added under inert atmosphere. The sample was characterized by ^{1}H-, ^{11}B{^{1}H}- and COSY-NMR spectroscopy.

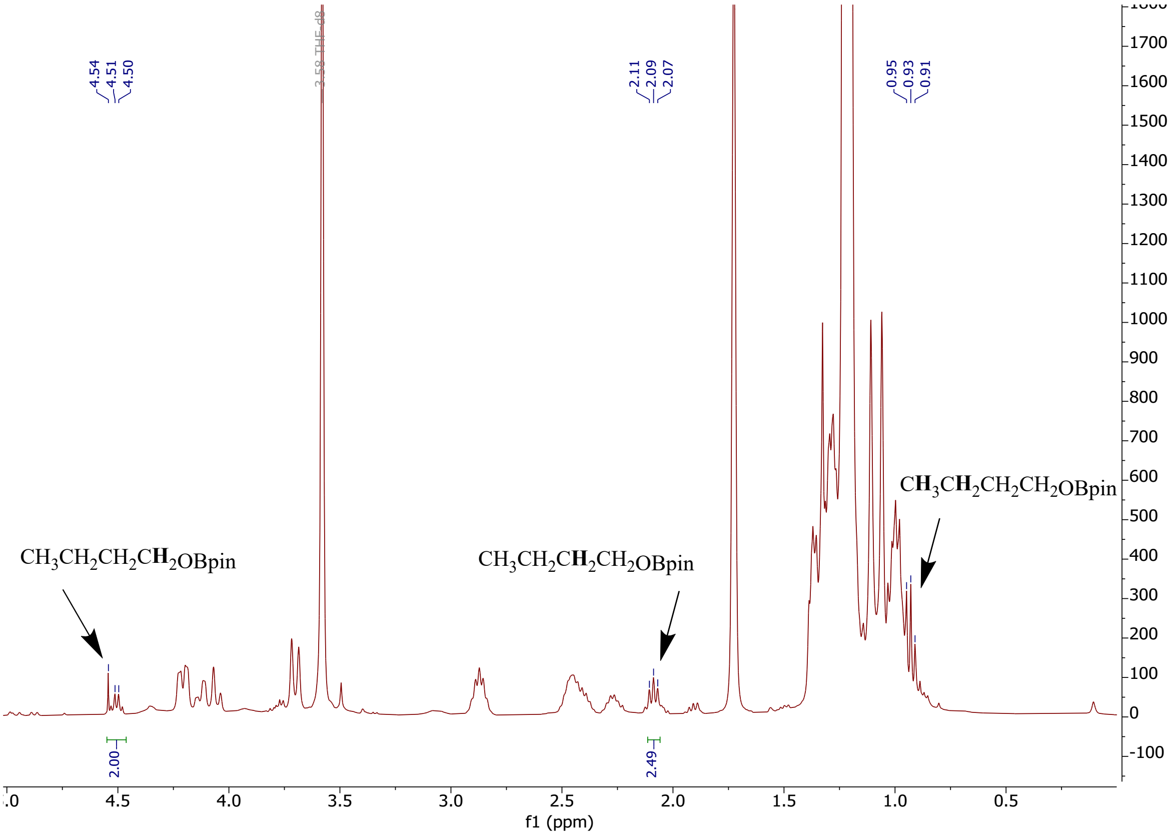

Figure S3. ^{1}H-NMR spectrum of complex 3 with 5 equiv. HBpin in THF-d_8.

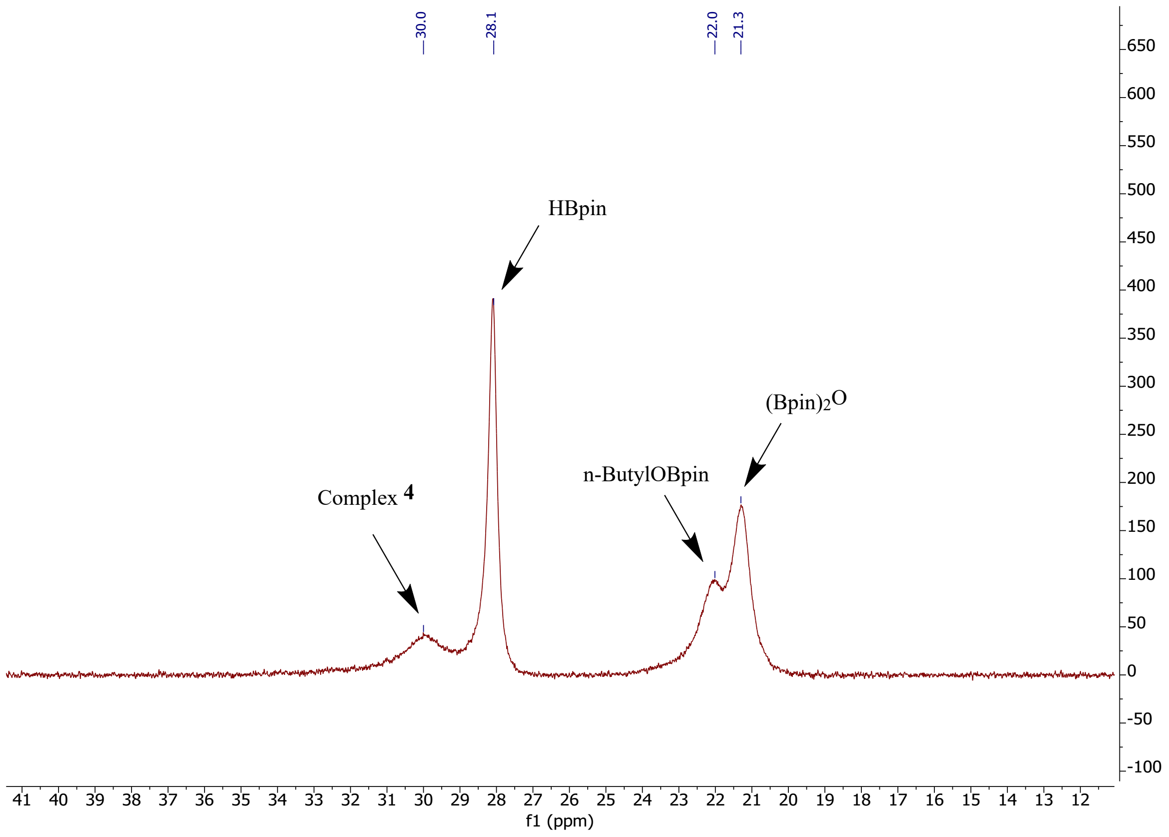

Figure S4. $^{11}B\{^1H\}$-NMR spectrum of complex 3 with 5 equiv. of HBpin in THF-d$_8$.

X-ray Structure Determination. X-ray diffraction data of **3** and **4** (CCDC 2308703, 2308704) were collected at T = 100 K in a dry stream of nitrogen on a Bruker Kappa APEX II diffractometer system using graphite-monochromatized Mo-$K\alpha$ radiation (λ = 0.71073 Å) and fine sliced φ- and ω-scans. Data were reduced to intensity values with SAINT and a correction for absorption effects was applied with the multi-scan approach followed by a spherical absorption correction using SADABS or TWINABS.[4] The structures were solved by the dual-space approach implemented in SHELXT[5] and refined against F^2 with SHELXL.[6] Non-hydrogen atoms were refined with anisotropic displacement parameters. H atoms attached to C were placed in calculated positions and thereafter refined as riding on the parent atoms. The positions of boride hydrogen atoms were refined freely. Molecular graphics were generated with the program MERCURY.[7]

Computational details. Calculations were performed using the GAUSSIAN 09 software package[8] and the PBE0 functional, without symmetry constraints. That functional uses a hybrid generalized gradient approximation (GGA), including 25 % mixture of Hartree-Fock[9] exchange with DFT[10] exchange-correlation, given by Perdew, Burke and Ernzerhof functional (PBE).[11] The optimized geometries were obtained with the Stuttgart Effective Core Potentials and associated basis set (SDD)[12] for Mn, and a standard 6-31G(d,p)[13] for the remaining elements (basis b1). Transition state optimizations were performed with the Synchronous Transit-Guided Quasi-Newton Method (STQN) developed by Schlegel *et al*,[14] following extensive searches of the Potential Energy Surface. Frequency calculations were performed to confirm the nature of the stationary points, yielding one imaginary frequency for the transition states and none for the minima. Each transition state was further confirmed by following its vibrational mode downhill on both sides and obtaining the minima presented on the energy profile. The electronic energies (E_{b1}) obtained at the PBE0/b1 level of theory were converted to free energy at 298.15 K and 1 atm (G_{b1}) by using zero point energy and thermal energy corrections based on structural and vibration frequency data calculated at the same level.

Single point energy calculations were performed on the geometries obtained at the PBE0/b1 level using the same functional and a 6-311++G(d,p) basis set.[15] The free energy values presented (G_{b2}-D3) were corrected for dispersion by means of Grimme DFT-D3 method[16] with Becke and Johnson short distance damping,[17] being

derived from the electronic energy values obtained at the PBE0-D3/6-311++G(d,p)//PBE0/b1 level (E_{b2}-D3) according to the following expression: (G_{b2}-D3) = (E_{b2}-D3) + G_{b1} – E_{b1}

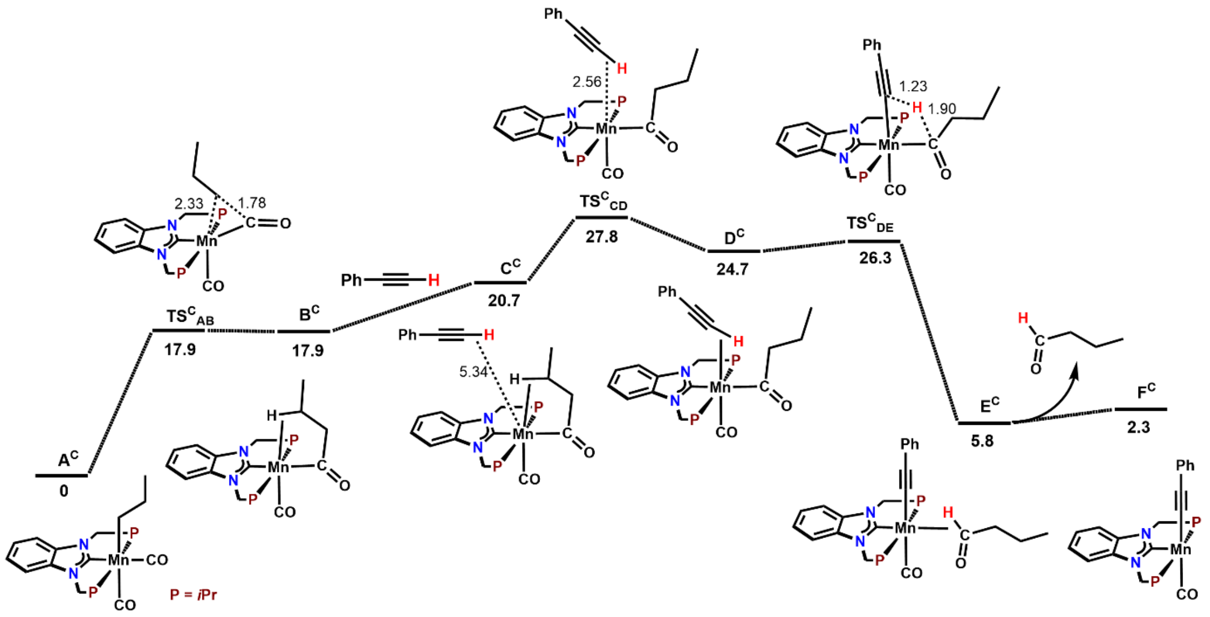

Figure S5. Free energy profile of the catalyst activation with phenylacetylene. Free energies (kcal/mol) are referred to *cis*-[Mn(PCP-*i*Pr)(CO)$_2$(CH$_2$CH$_2$CH$_3$)] (**A**$^{\mathbf{C}}$) (pre-catalyst activation).

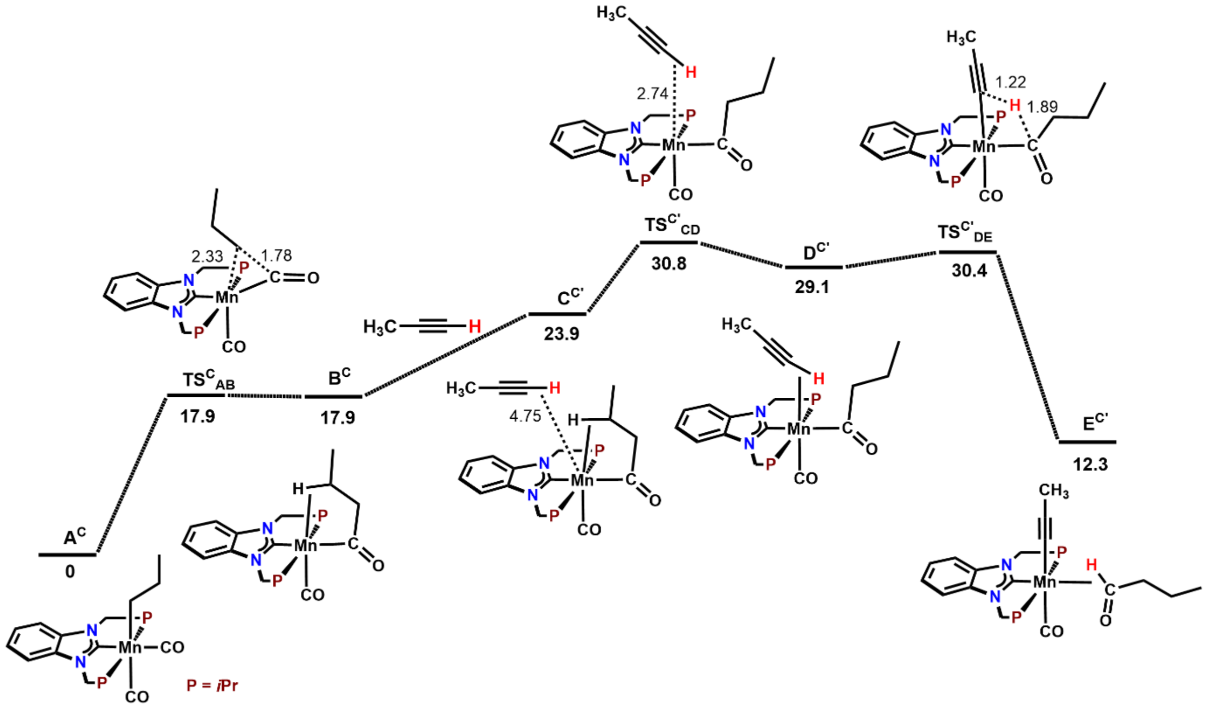

Figure S6. Free energy profile of the catalyst activation with propyne. Free energies (kcal/mol) are referred to *cis*-[Mn(PCP-*i*Pr)(CO)$_2$(CH$_2$CH$_2$CH$_3$)] (**A**$^{\mathbf{C}}$) (pre-catalyst activation).

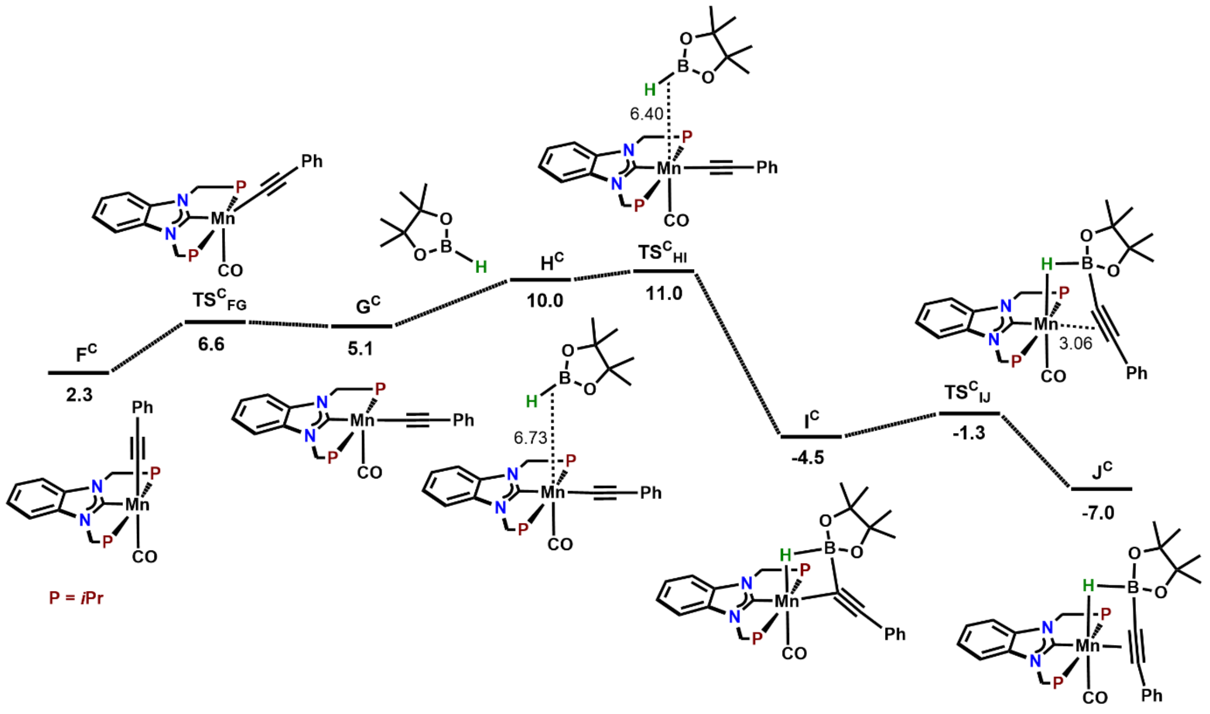

Figure S7. Free energy profile of the hydroboration of phenylacetylene. Formation of the *Z*-isomer. Free energies (kcal/mol) are referred to *cis*-[Mn(PCP-*i*Pr)(CO)$_2$(CH$_2$CH$_2$CH$_3$)] (**A**^{C}) (hydroboration steps).

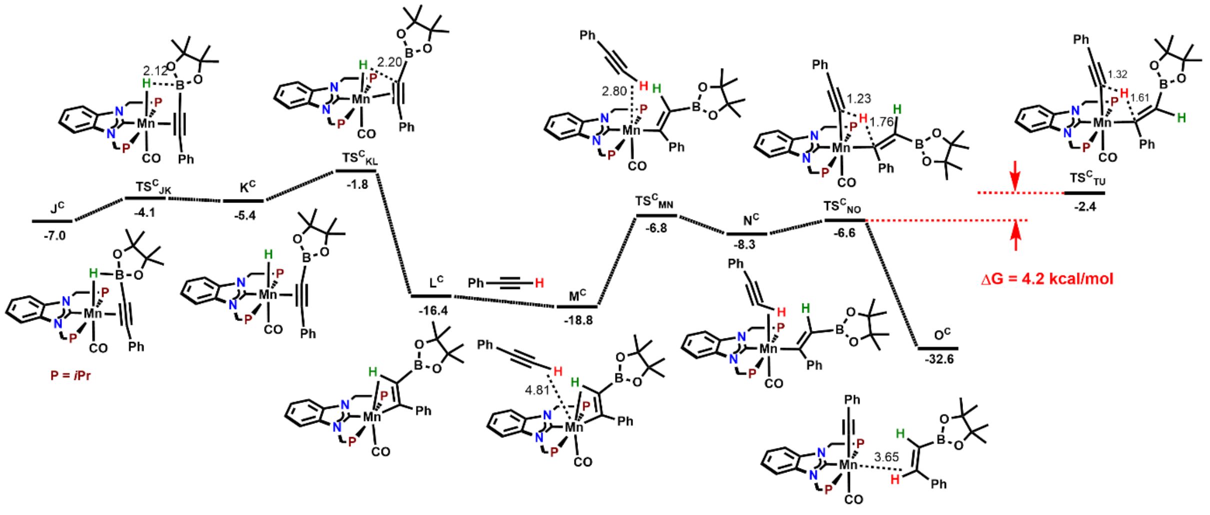

Figure S8. Free energy profile of the hydroboration of phenylacetylene. Formation of the *Z*-isomer. Free energies (kcal/mol) are referred to *cis*-[Mn(PCP-*i*Pr)(CO)$_2$(CH$_2$CH$_2$CH$_3$)] (**A**^{C}) (hydroboration steps).

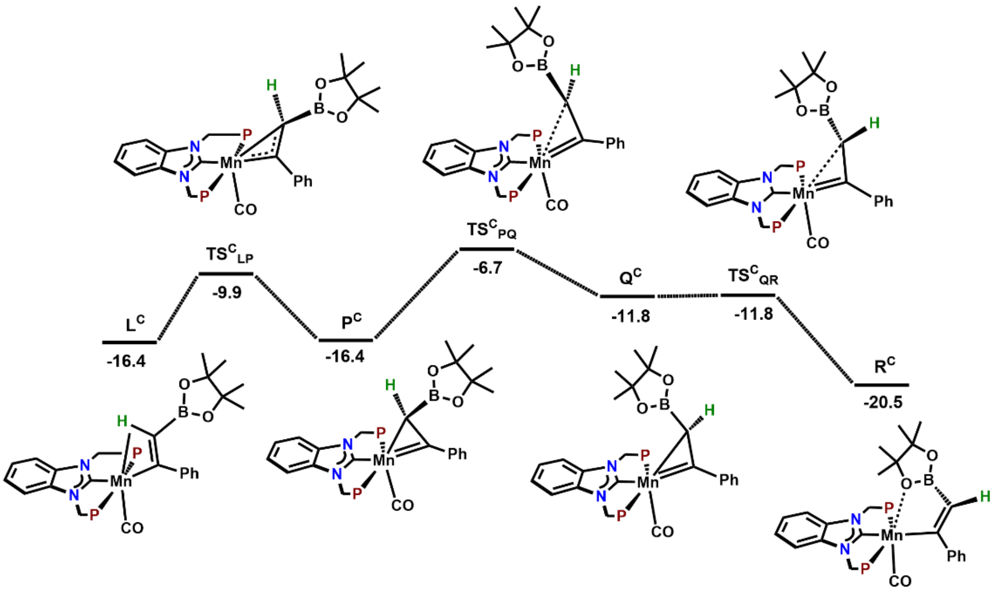

Figure S9. Free energy profile of the hydroboration of phenylacetylene. Isomerization to the *E*-Isomer. Free energies (kcal/mol) are referred to *cis*-[Mn(PCP-*i*Pr)(CO)$_2$(CH$_2$CH$_2$CH$_3$)] ($\mathbf{A^C}$) (hydroboration steps).

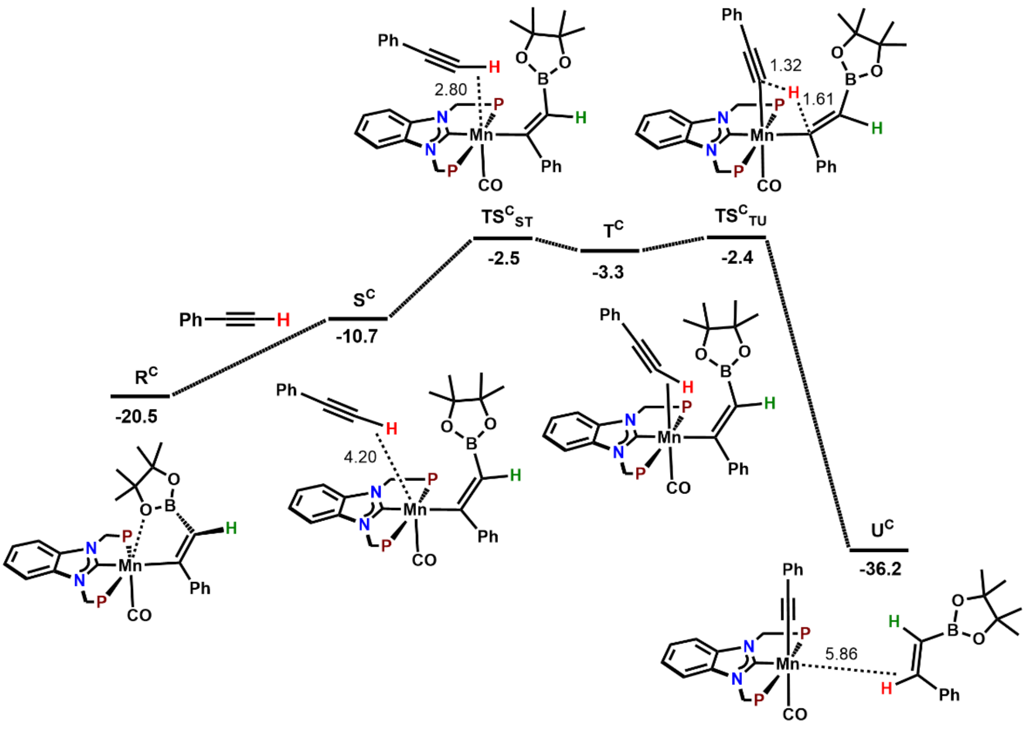

Figure S10. Free energy profile of the hydroboration of phenylacetylene. Isomerization to the *E*-Isomer. Free energies (kcal/mol) are referred to *cis*-[Mn(PCP-*i*Pr)(CO)$_2$(CH$_2$CH$_2$CH$_3$)] ($\mathbf{A^C}$) (hydroboration steps).

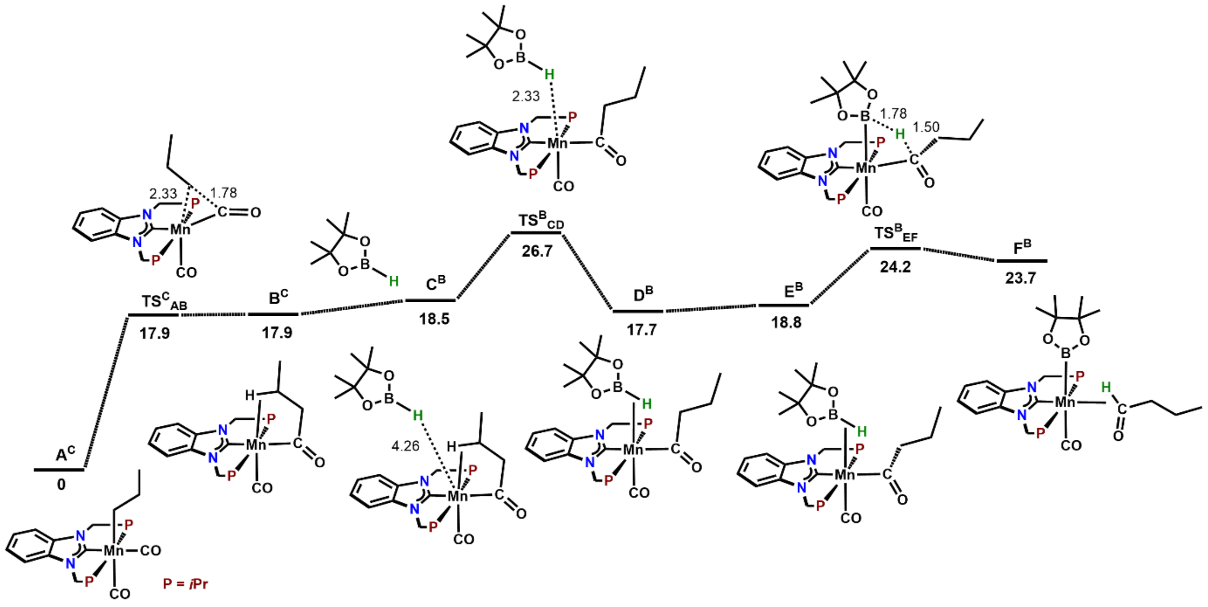

Figure S11. Free energy profile of the hydroboration of propyne. Free energies (kcal/mol) are referred to *cis*-[Mn(PCP-*i*Pr)(CO)$_2$(CH$_2$CH$_2$CH$_3$)] (**A^C**) (pre-catalyst activation).

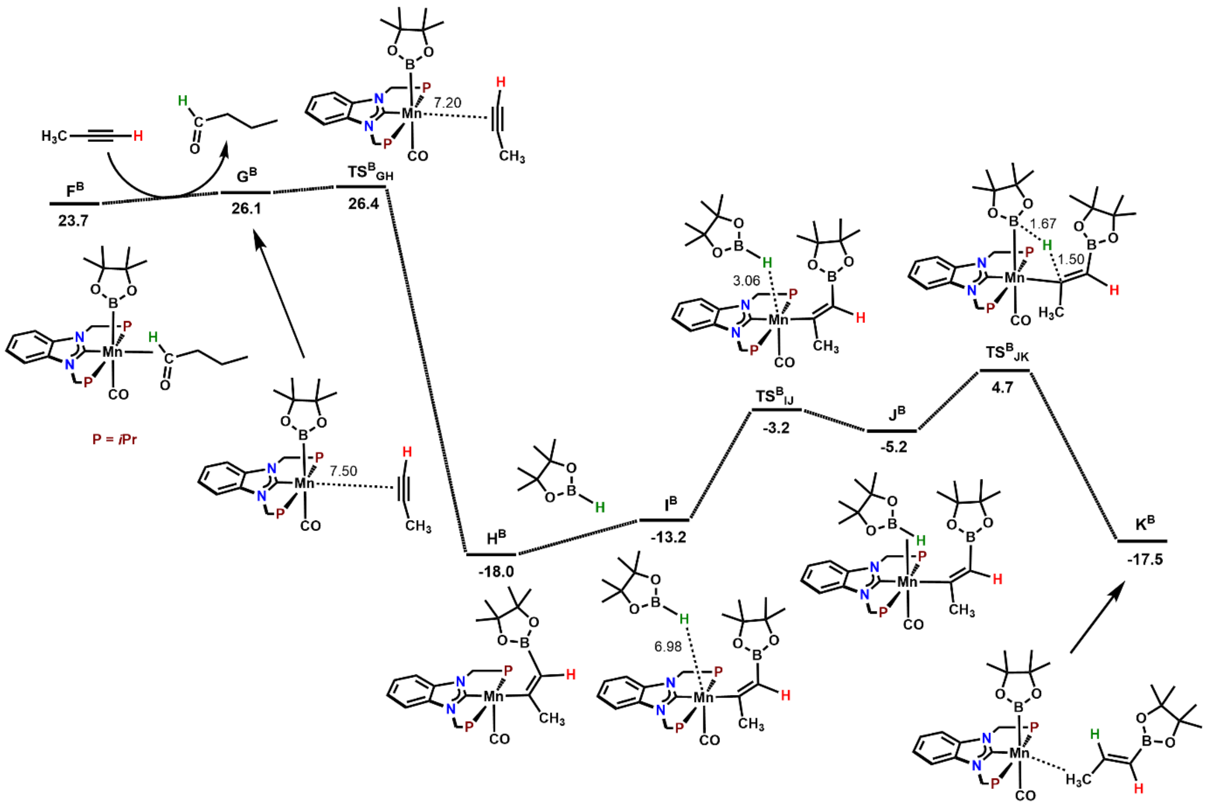

Figure S12. Free energy profile of the hydroboration of propyne. Formation of the *E*-isomer. Free energies (kcal/mol) are referred to *cis*-[Mn(PCP-*i*Pr)(CO)$_2$(CH$_2$CH$_2$CH$_3$)] (**A^C**) (hydroboration steps).

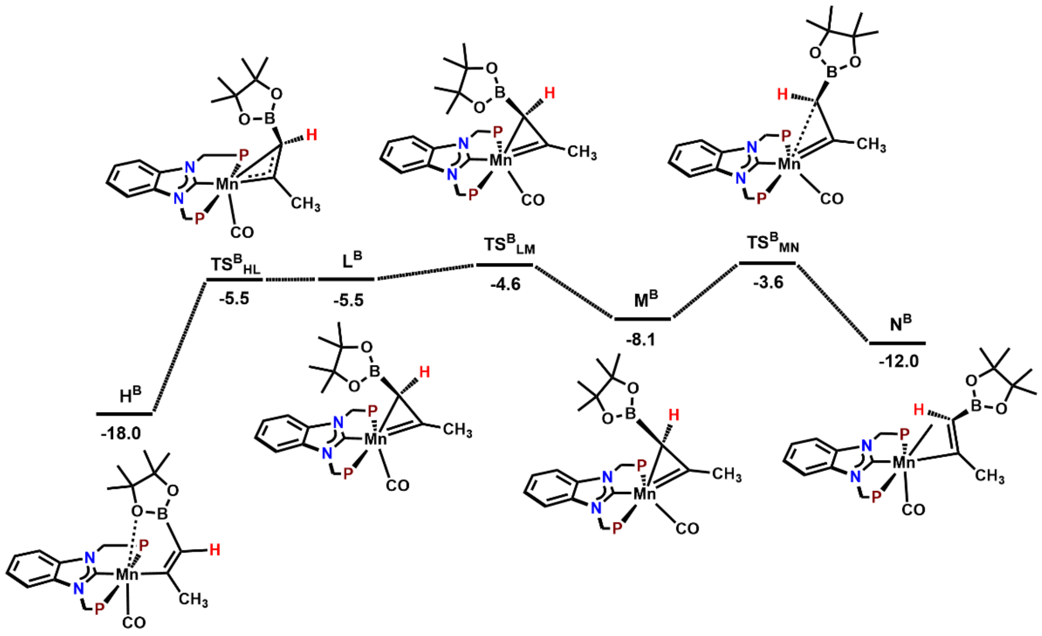

Figure S13. Free energy profile of the hydroboration of propyne. Isomerization to the *Z*-Isomer. Free energies (kcal/mol) are referred to *cis*-[Mn(PCP-*i*Pr)(CO)$_2$(CH$_2$CH$_2$CH$_3$)] (**A**^{C}) (hydroboration steps).

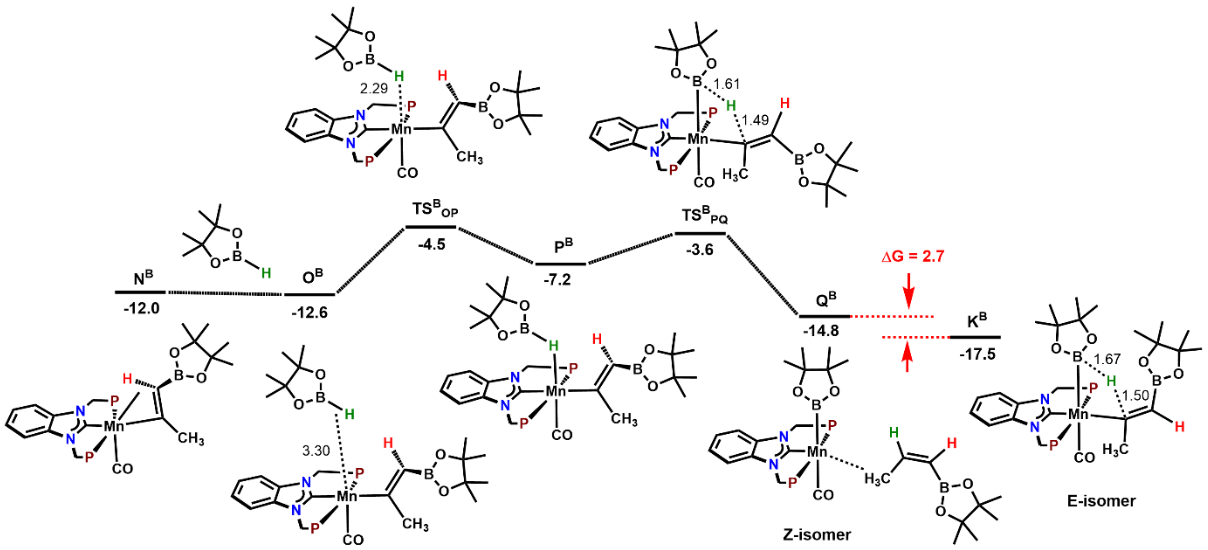

Figure S14. Free energy profile of the hydroboration of propyne. Isomerization to the *Z*-Isomer. Free energies (kcal/mol) are referred to *cis*-[Mn(PCP-*i*Pr)(CO)$_2$(CH$_2$CH$_2$CH$_3$)] (**A**^{C}) (hydroboration steps).

Characterization of Organic Products

4,4,5,5-Tetramethyl-2-[(1*Z*)-2-phenylethenyl]-1,3,2-dioxaborolane (5a)

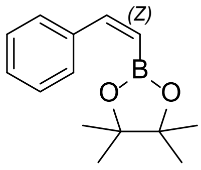

Phenylacetylene (55 µL, 0.50 mmol, 1.0 equiv.); pinacolborane (80 µL, 0.55 mmol, 1.1 equiv.); catalyst (2.7 mg, 5.0 µmol, 1.0 mol%); 114 mg (99%, E/Z: 3/97) of a yellow oil
^{1}H NMR (400 MHz, $CDCl_3$): δ = 7.58 – 7.50 (m, 2H), 7.36 – 7.27 (m, 3H), 7.25 – 7.18 (m, 1H), 5.60 (d, *J* = 14.9 Hz, 1H), 1.29 (s, 12H) ppm.
^{13}C{^{1}H} NMR (101 MHz, $CDCl_3$): δ = 148.3, 138.6, 128.8, 128.1, 128.1, 83.6, 24.9 ppm.
These spectroscopic data are in accordance with literature.[18]

4,4,5,5-Tetramethyl-2-[(1*Z*)-2-(4-methylphenyl)ethenyl]-1,3,2-dioxaborolane (5b)

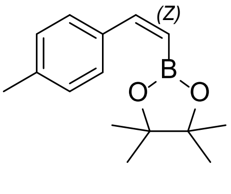

4-Ethynyltoluene (63 µL, 0.50 mmol, 1.0 equiv.); pinacolborane (80 µL, 0.55 mmol, 1.1 equiv.); catalyst (2.7 mg, 5.0 µmol, 1.0 mol%); 120 mg (98%, E/Z: 7/93) of a yellow oil
^{1}H NMR (400 MHz, $CDCl_3$): δ = 7.49 – 7.43 (m, 2H), 7.18 (d, *J* = 15.4 Hz, 1H), 7.15 – 7.09 (m, 2H), 5.52 (d, *J* = 14.9 Hz, 1H), 2.34 (s, 3H), 1.30 (s, 12H) ppm.
^{13}C{^{1}H} NMR (101 MHz, $CDCl_3$): δ = 148.3, 138.1, 135.8, 128.8, 128.8, 83.6, 25.0, 21.4 ppm.
These spectroscopic data are in accordance with literature.[18]

2-[(1*Z*)-2-[4-(1,1-Dimethylethyl)phenyl]ethenyl]-4,4,5,5-tetramethyl-1,3,2-dioxaborolane (5c)

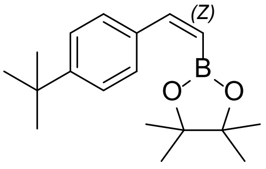

1-(*tert*-Butyl)-4-ethynylbenzene (87 µL, 0.50 mmol, 1.0 equiv.); pinacolborane (80 µL, 0.55 mmol, 1.1 equiv.); catalyst (2.7 mg, 5.0 µmol, 1.0 mol%); 139 mg (97%, E/Z: 8/92) of a yellow oil
^{1}H NMR (400 MHz, $CDCl_3$): δ = 7.55 – 7.50 (m, 2H), 7.35 – 7.31 (m, 2H), 7.17 (d, *J* = 15.0 Hz, 1H), 5.53 (d, *J* = 14.9 Hz, 1H), 1.32 (s, 9H), 1.31 (s, 12H) ppm.
^{13}C{^{1}H} NMR (101 MHz, $CDCl_3$): δ = 151.3, 148.2, 135.7, 128.6, 125.1, 83.6, 34.8, 31.4, 25.0 ppm.
These spectroscopic data are in accordance with literature.[19]

2-[(1*Z*)-2-(4-Methoxyphenyl)ethenyl]-4,4,5,5-tetramethyl-1,3,2-dioxaborolane (5d)

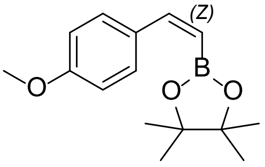

4-Ethynylanisole (65 µL, 0.50 mmol, 1.0 equiv.); pinacolborane (80 µL, 0.55 mmol, 1.1 equiv.); catalyst (2.7 mg, 5.0 µmol, 1.0 mol%); 129 mg (99%, E/Z: 3/97) of a yellow oil
^{1}H NMR (400 MHz, $CDCl_3$): δ = 7.58 – 7.50 (m, 2H), 7.15 (d, *J* = 14.9 Hz, 1H), 6.90 – 6.80 (m, 2H), 5.45 (d, *J* = 14.9 Hz, 1H), 3.82 (s, 3H), 1.30 (s, 12H) ppm.
^{13}C{^{1}H} NMR (101 MHz, $CDCl_3$): δ = 159.7, 148.2, 131.4, 130.4, 113.5, 83.5, 55.4, 25.0 ppm.
These spectroscopic data are in accordance with literature.[18]

2-[(1*Z*)-2-(4-Fluorophenyl)ethenyl]-4,4,5,5-tetramethyl-1,3,2-dioxaborolane (5e)

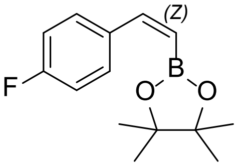

1-Ethynyl-4-fluorobenzene (57 µL, 0.50 mmol, 1.0 equiv.); pinacolborane (80 µL, 0.55 mmol, 1.1 equiv.); catalyst (2.7 mg, 5.0 µmol, 1.0 mol%); 123 mg (99%, E/Z: 4/96) of a yellow oil
^{1}H NMR (400 MHz, $CDCl_3$): δ = 7.58 – 7.50 (m, 2H), 7.17 (d, *J* = 14.7 Hz, 1H), 7.03 – 6.94 (m, 2H), 5.56 (d, *J* = 14.8 Hz, 1H), 1.29 (s, 12H) ppm.
^{13}C{^{1}H} NMR (101 MHz, $CDCl_3$): δ = 162.8 (d, *J* = 247.3 Hz), 147.4, 134.7 (d, *J* = 3.2 Hz), 130.6 (d, *J* = 8.1 Hz), 114.9 (d, *J* = 21.5 Hz), 83.7, 24.9 ppm.
These spectroscopic data are in accordance with literature.[19]

2-[(1*Z*)-2-(4-Chlorophenyl)ethenyl]-4,4,5,5-tetramethyl-1,3,2-dioxaborolane (5f)

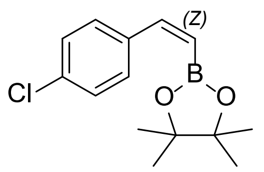

4-Chloro-1-ethynylbenzene (68 mg, 0.50 mmol, 1.0 equiv.); pinacolborane (80 µL, 0.55 mmol, 1.1 equiv.); catalyst (2.7 mg, 5.0 µmol, 1.0 mol%); 122 mg (92%, E/Z: 3/97) of a yellow oil

^{1}H NMR (400 MHz, $CDCl_3$): δ = 7.51 – 7.46 (m, 2H), 7.30 – 7.27 (m, 1H), 7.27 – 7.25 (m, 1H), 7.15 (d, *J* = 14.9 Hz, 1H), 5.61 (d, *J* = 14.9 Hz, 1H), 1.29 (s, 12H) ppm.

^{13}C{^{1}H} NMR (101 MHz, $CDCl_3$): δ = 147.1, 137.0, 133.9, 130.1, 128.2, 83.7, 24.9 ppm.

These spectroscopic data are in accordance with literature.[20]

2-[(1*Z*)-2-(4-Ethenylphenyl)ethenyl]-4,4,5,5-tetramethyl-1,3,2-dioxaborolane (5g)

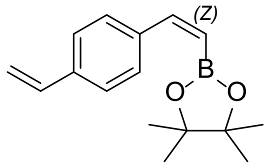

4-Ethynylstyrene (64 mg, 0.50 mmol, 1.0 equiv.); pinacolborane (80 µL, 0.55 mmol, 1.1 equiv.); catalyst (2.7 mg, 5.0 µmol, 1.0 mol%); 106 mg (83%, E/Z: 4/96) of a yellow oil

^{1}H NMR (400 MHz, $CDCl_3$): δ = 7.57 – 7.49 (m, 2H), 7.38 – 7.30 (m, 2H), 7.18 (d, *J* = 14.9 Hz, 1H), 6.71 (dd, *J* = 17.6, 10.9 Hz, 1H), 5.76 (dd, *J* = 17.6, 1.0 Hz, 1H), 5.58 (d, *J* = 14.9 Hz, 1H), 5.25 (dd, *J* = 10.9, 0.9 Hz, 1H), 1.30 (s, 12H) ppm.

^{13}C{^{1}H} NMR (101 MHz, $CDCl_3$): δ = 147.9, 138.1, 137.4, 136.7, 129.1, 126.0, 114.0, 83.7, 25.0 ppm.

***N*,*N*-Dimethyl-4-[(1*Z*)-2-(4,4,5,5-tetramethyl-1,3,2-dioxaborolan-2-yl)ethenyl]benzenamine (5h)**

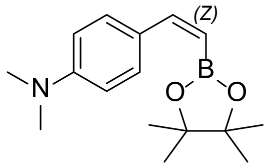

N,N-Dimethyl-4-ethynylaniline (73 mg, 0.50 mmol, 1.0 equiv.); pinacolborane (80 µL, 0.55 mmol, 1.1 equiv.); catalyst (2.7 mg, 5.0 µmol, 1.0 mol%); 132 mg (97%, E/Z: 4/96) of a yellow oil

^{1}H NMR (400 MHz, $CDCl_3$): δ = 7.60 – 7.52 (m, 2H), 7.11 (d, *J* = 14.9 Hz, 1H), 6.67 – 6.63 (m, 2H), 5.31 (d, *J* = 14.9 Hz, 1H), 2.98 (s, 6H), 1.31 (s, 12H) ppm.

^{13}C{^{1}H} NMR (101 MHz, $CDCl_3$): δ = 150.6, 149.1, 130.4, 127.1, 111.7, 83.3, 40.5, 25.0 ppm.

These spectroscopic data are in accordance with literature.[19]

4-[(1*Z*)-2-(4,4,5,5-Tetramethyl-1,3,2-dioxaborolan-2-yl)ethenyl]benzenemethanol (5i)

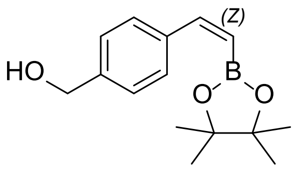

4-Ethynylbenzaldehyde (65 mg, 0.50 mmol, 1.0 equiv.); pinacolborane (153 µL, 1.05 mmol, 2.1 equiv.); catalyst (4.0 mg, 7.5 µmol, 1.5 mol%); 101 mg (77%, E/Z: 3/97) of a yellow oil

^{1}H NMR (400 MHz, $CDCl_3$): δ = 7.58 – 7.51 (m, 2H), 7.33 – 7.28 (m, 2H), 7.20 (d, *J* = 15.1 Hz, 1H), 5.59 (d, *J* = 14.9 Hz, 1H), 4.69 (d, *J* = 5.8 Hz, 2H), 1.73 – 1.65 (m, 1H), 1.29 (s, 12H) ppm.

^{13}C{^{1}H} NMR (101 MHz, $CDCl_3$): δ = 147.9, 140.8, 138.0, 129.0, 126.7, 83.7, 65.3, 24.9 ppm.

4-[(1*Z*)-2-(4,4,5,5-Tetramethyl-1,3,2-dioxaborolan-2-yl)ethenyl]benzene-1-ethanol (5j)

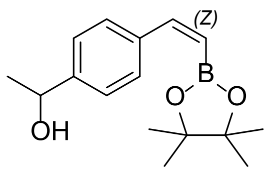

1-(4-Ethynylphenyl)ethanone (72 mg, 0.50 mmol, 1.0 equiv.); pinacolborane (153 µL, 1.05 mmol, 2.1 equiv.); catalyst (4.0 mg, 7.5 µmol, 1.5 mol%); 133 mg (97%, E/Z: 9/91) of a yellow oil

^{1}H NMR (400 MHz, $CDCl_3$): δ = 7.58 – 7.52 (m, 2H), 7.34 – 7.29 (m, 2H), 7.19 (d, *J* = 15.0 Hz, 1H), 5.58 (d, *J* = 14.9 Hz, 1H), 4.95 – 4.85 (m, 1H), 1.81 (d, *J* = 3.5 Hz, 1H), 1.49 (d, *J* = 6.5 Hz, 3H), 1.30 (s, 12H) ppm.

^{13}C{^{1}H} NMR (101 MHz, $CDCl_3$): δ = 147.9, 145.8, 137.8, 129.0, 125.2, 83.7, 70.4, 25.2, 24.9 ppm.

HR-MS: *m*/*z* calcd for $C_{16}H_{23}BO_3Na$ $[M+Na]^+$ 297.1632, found 297.1636.

4,4,5,5-Tetramethyl-2-[(1*Z*)-2-(3-methylphenyl)ethenyl]-1,3,2-dioxaborolane (5k)

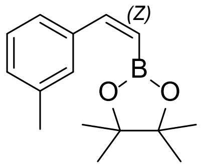

3-Ethynyltoluene (65 µL, 0.50 mmol, 1.0 equiv.); pinacolborane (80 µL, 0.55 mmol, 1.1 equiv.); catalyst (2.7 mg, 5.0 µmol, 1.0 mol%); 121 mg (99%, E/Z: 5/95) of a yellow oil

^{1}H NMR (400 MHz, $CDCl_3$): δ = 7.42 – 7.38 (m, 1H), 7.33 – 7.28 (m, 1H), 7.23 – 7.16 (m, 2H), 7.12 – 7.05 (m, 1H), 5.57 (d, *J* = 14.9 Hz, 1H), 2.34 (s, 3H), 1.30 (s, 12H) ppm.

^{13}C{^{1}H} NMR (101 MHz, $CDCl_3$): δ = 148.3, 138.55, 136.0, 129.3, 128.9, 128.0, 126.0, 83.6, 25.0, 21.5 ppm.

These spectroscopic data are in accordance with literature.[21]

2-[(1*Z*)-2-(3-Bromophenyl)ethenyl]-4,4,5,5-tetramethyl-1,3,2-dioxaborolane (5l)

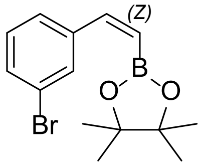

3-Bromo-1-ethynylbenzene (62 µL, 0.50 mmol, 1.0 equiv.); pinacolborane (80 µL, 0.55 mmol, 1.1 equiv.); catalyst (2.7 mg, 5.0 µmol, 1.0 mol%); product was purified via column chromatography, eluent: petroleum ether and diethyl ether (9:1); 76 mg (49%, E/Z: 1/99) of a yellow oil

^{1}H NMR (400 MHz, $CDCl_3$): δ = 7.82 (t, *J* = 1.9 Hz, 1H), 7.42 – 7.32 (m, 2H), 7.23 – 7.09 (m, 2H), 5.65 (d, *J* = 14.8 Hz, 1H), 1.31 (s, 12H) ppm.

^{13}C{^{1}H} NMR (101 MHz, $CDCl_3$) δ 146.6, 140.6, 131.3, 131.0, 129.6, 127.7, 122.3, 83.9, 25.0 ppm.

2-[(1*Z*)-2-(2-Methylphenyl)ethenyl]-4,4,5,5-tetramethyl-1,3,2-dioxaborolane (5m)

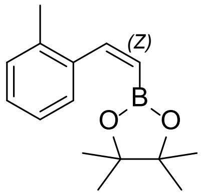

2-Ethynyltoluene (63 µL, 0.50 mmol, 1.0 equiv.); pinacolborane (80 µL, 0.55 mmol, 1.1 equiv.); catalyst (2.7 mg, 5.0 µmol, 1.0 mol%); reaction temperature: 70°C; 120 mg (98%, E/Z: 7/93) of a yellow oil

^{1}H NMR (400 MHz, $CDCl_3$): δ = 7.44 – 7.33 (m, 2H), 7.20 – 7.08 (m, 3H), 5.65 (d, *J* = 14.5 Hz, 1H), 2.32 (s, 3H), 1.23 (s, 12H) ppm.

^{13}C{^{1}H} NMR (101 MHz, $CDCl_3$): δ = 147.0, 138.1, 136.1, 129.8, 128.8, 128.1, 125.3, 83.5, 24.8, 20.0 ppm.

These spectroscopic data are in accordance with literature.[20]

2-[(1*Z*)-2-(2-Fluorophenyl)ethenyl]-4,4,5,5-tetramethyl-1,3,2-dioxaborolane (5n)

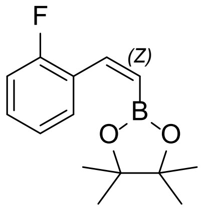

1-Ethynyl-2-fluorobenzene (57 µL, 0.50 mmol, 1.0 equiv.); pinacolborane (80 µL, 0.55 mmol, 1.1 equiv.); catalyst (2.7 mg, 5.0 µmol, 1.0 mol%); 68 mg (55%, E/Z: 4/96) of a yellow oil

^{1}H NMR (400 MHz, $CDCl_3$): δ = 7.61 (td, *J* = 7.7, 1.8 Hz, 1H), 7.32 (d, *J* = 14.8 Hz, 1H), 7.26 – 7.20 (m, 1H), 7.10 – 6.97 (m, 2H), 5.74 (d, *J* = 14.8 Hz, 1H), 1.27 (s, 12H) ppm.

^{13}C{^{1}H} NMR (101 MHz, $CDCl_3$): δ 160.5 (d, *J* = 248.8 Hz), 140.4 (d, *J* = 4.1 Hz), 130.3 (d, *J* = 3.2 Hz), 129.8 (d, *J* = 8.4 Hz), 126.6 (d, *J* = 12.6 Hz), 123.5 (d, *J* = 3.7 Hz), 115.3 (d, *J* = 22.0 Hz), 83.7, 24.9 ppm.

2,2′-[1,3-phenylenedi-(1*Z*)-2,1-ethenediyl]bis[4,4,5,5-tetramethyl-1,3,2-dioxaborolane] (5o)

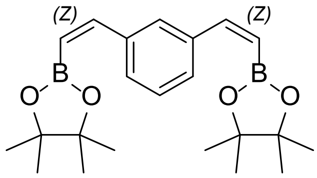

1,3-Diethynylbenzene (66 µL, 0.50 mmol, 1.0 equiv.); pinacolborane (153 µL, 1.05 mmol, 2.1 equiv.); catalyst (5.4 mg, 10 µmol, 2.0 mol%); product was purified *via* column chromatography, eluent: petroleum ether and diethyl ether (9:1); 143 mg (75%, E/Z: 5/95) of a slight yellow solid

^{1}H NMR (400 MHz, $CDCl_3$): δ = 7.62 – 7.60 (m, 1H), 7.48 (d, *J* = 1.8 Hz, 1H), 7.46 (d, *J* = 1.8 Hz, 1H), 7.28 – 7.23 (m, 1H), 7.20 (d, J = 14.8 Hz, 2H), 5.59 (d, *J* = 14.8 Hz, 2H), 1.28 (s, 24H) ppm.

^{13}C{^{1}H} NMR (101 MHz, $CDCl_3$): δ = 148.2, 138.4, 129.4, 128.3, 127.7, 83.6, 25.0 ppm.

These spectroscopic data are in accordance with literature.[19]

4,4,5,5-Tetramethyl-2-[(1*Z*)-2-(1-naphthalenyl)ethenyl]-1,3,2-dioxaborolane (5p)

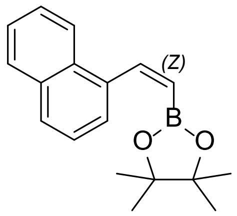

1-Ethynylnaphthalene (71 µL, 0.50 mmol, 1.0 equiv.); pinacolborane (80 µL, 0.55 mmol, 1.1 equiv.); catalyst (2.7 mg, 5.0 µmol, 1.0 mol%); reaction temperature: 70°C; 116 mg (83%, E/Z: 7/93) of a yellow oil

^{1}H NMR (400 MHz, $CDCl_3$): δ = 8.08 – 7.98 (m, 1H), 7.91 – 7.70 (m, 3H), 7.58 – 7.35 (m, 4H), 5.88 (d, *J* = 14.4 Hz, 1H), 1.16 (s, 12H) ppm.

^{13}C{^{1}H} NMR (101 MHz, $CDCl_3$): δ = 146.3, 136.5, 133.4, 131.7, 128.5, 128.4, 126.5, 126.1, 125.8, 125.2, 124.8, 83.4, 24.8 ppm.

These spectroscopic data are in accordance with literature.[20]

4,4,5,5-Tetramethyl-2-[(1*Z*)-2-(2-naphthalenyl)ethenyl]-1,3,2-dioxaborolane (5q)

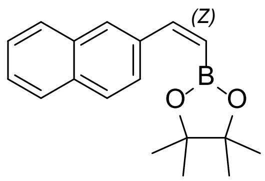

2-Ethynylnaphthalene (76 µL, 0.50 mmol, 1.0 equiv.); pinacolborane (80 µL, 0.55 mmol, 1.1 equiv.); catalyst (2.7 mg, 5.0 µmol, 1.0 mol%); 133 mg (95%, E/Z: 9/91) of a yellow oil

^{1}H NMR (400 MHz, $CDCl_3$): δ = 8.00 – 7.96 (m, 1H), 7.82 – 7.71 (m, 4H), 7.49 – 7.42 (m, 2H), 7.39 (d, *J* = 15.0 Hz, 1H), 5.69 (d, *J* = 14.8 Hz, 1H), 1.32 (s, 12H) ppm.

^{13}C{^{1}H} NMR (101 MHz, $CDCl_3$): δ = 148.3, 136.2, 133.4, 133.3, 128.3, 128.2, 127.7, 127.5, 126.7, 126.2, 126.1, 83.7, 25.0 ppm.

These spectroscopic data are in accordance with literature.[21]

4,4,5,5-Tetramethyl-2-[(1*Z*)-2-(3-thienyl)ethenyl]-1,3,2-dioxaborolane (5r)

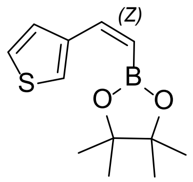

3-Ethynylthiophene (49 µL, 0.50 mmol, 1.0 equiv.); pinacolborane (80 µL, 0.55 mmol, 1.1 equiv.); catalyst (2.7 mg, 5.0 µmol, 1.0 mol%); 117 mg (99%, E/Z: 7/93) of a yellow oil

^{1}H NMR (400 MHz, $CDCl_3$): δ = 7.69 – 7.64 (m, 1H), 7.59 – 7.53 (m, 1H), 7.25 – 7.20 (m, 1H), 7.16 (d, *J* = 15.1 Hz, 1H), 5.47 (d, *J* = 15.1 Hz, 1H), 1.32 (s, 12H) ppm.

^{13}C{^{1}H} NMR (101 MHz, $CDCl_3$): δ = 142.1, 140.8, 128.8, 126.4, 125.0, 83.6, 25.0 ppm.

These spectroscopic data are in accordance with literature.[19]

4,4,5,5-Tetramethyl-2-[(1*Z*)-2-(trimethylsilyl)ethenyl]-1,3,2-dioxaborolane (5s)

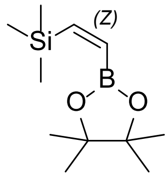

Trimethylsilylacetylene (71 µL, 0.50 mmol, 1.0 equiv.); pinacolborane (80 µL, 0.55 mmol, 1.1 equiv.); catalyst (2.7 mg, 5.0 µmol, 1.0 mol%); 100 mg (88%, E/Z: 1/99) of a yellow oil

^{1}H NMR (400 MHz, $CDCl_3$): δ = 6.87 (d, *J* = 18.9 Hz, 1H), 6.41 (d, *J* = 18.8 Hz, 1H), 1.27 (s, 12H), 0.15 (s, 9H) ppm.

^{13}C{^{1}H} NMR (101 MHz, $CDCl_3$): δ = 158.2, 83.5, 25.0, -0.1 ppm.

These spectroscopic data are in accordance with literature.[22]

2-[(1*Z*)-2-(1-Cyclohexen-1-yl)ethenyl]-4,4,5,5-tetramethyl-1,3,2-dioxaborolane (5t)

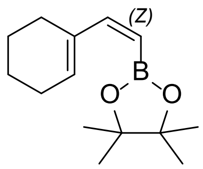

1-Ethynylcyclohexene (59 µL, 0.50 mmol, 1.0 equiv.); pinacolborane (80 µL, 0.55 mmol, 1.1 equiv.); catalyst (2.7 mg, 5.0 µmol, 1.0 mol%); 85 mg (73%, E/Z: 13/87) of a yellow oil

^{1}H NMR (400 MHz, $CDCl_3$): δ = 7.02 (d, *J* = 18.2 Hz, 0.1H, *E*-Isomer), 6.64 (d, *J* = 14.9 Hz, 0.9H *Z*-Isomer), 5.99 – 5.94 (m, 0.1H, *E*-Isomer), 5.87 – 5.79 (m, 0.9H, *Z*-Isomer), 5.43 (dd, *J* = 18.3, 0.8 Hz, 0.1H, *E*-Isomer) 5.17 (d, *J* = 14.8 Hz, 0.9H, *Z*-Isomer), 2.30 – 2.21 (m, 2H), 2.18 – 2.08 (m, 2H), 1.70 – 1.53 (m, 4H), 1.28 (s, 12H) ppm.

^{13}C{^{1}H} NMR (101 MHz, $CDCl_3$): δ = 153.4 (*E*-Isomer), 149.9 (*Z*-Isomer), 138.1, 134.4 (*E*-Isomer), 132.3 (*Z*-Isomer), 83.5 (*Z*-Isomer), 83.2 (*E*-Isomer), 26.4, 26.1, 24.9, 24.9, 22.6, 22.3 ppm.

These spectroscopic data are in accordance with literature.[23]

2-[(1*E*)-2-Cyclohexylethenyl]-4,4,5,5-tetramethyl-1,3,2-dioxaborolane (5u)

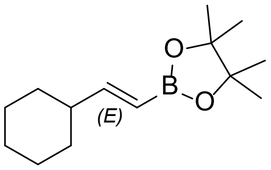

Cyclohexylacetylene (65 µL, 0.50 mmol, 1.0 equiv.); pinacolborane (80 µL, 0.55 mmol, 1.1 equiv.); catalyst (2.7 mg, 5.0 µmol, 1.0 mol%); reaction temperature: 70°C; 87 mg (74%, E/Z: 81/19) of a yellow oil

^{1}H NMR (400 MHz, $CDCl_3$): δ = 6.58 (dd, *J* = 18.2, 6.2 Hz, 0.8H, *E*-Isomer), 6.25 (dd, *J* = 13.5, 9.3 Hz, 0.2H, *Z*-Isomer), 5.37 (dd, *J* = 18.2, 1.5 Hz, 0.8H, E-Isomer), 5.22 (dd, *J* = 13.5, 0.9 Hz, 0.2H, *Z*-Isomer), 2.08 – 1.95 (m, 1H), 1.77 – 1.60 (m, 6H), 1.26 (s, 12H), 1.20 – 1.00 (m, 4H) ppm.

^{13}C{^{1}H} NMR (101 MHz, $CDCl_3$): δ = 160.8 (*Z*-Isomer), 160.0 (*E*-Isomer), 83.1 (*E*-Isomer), 82.9 (*Z*-Isomer), 43.4, 33.5 (*Z*-Isomer), 32.1 (*E*-Isomer), 26.3, 26.1, 24.9 ppm.

These spectroscopic data are in accordance with literature.[24]

4,4,5,5-Tetramethyl-2-(1*E*)-1-octen-1-yl-1,3,2-dioxaborolane (5v)

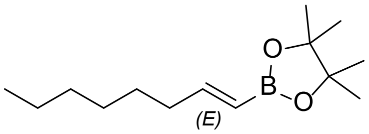

1-Octyne (74 µL, 0.50 mmol, 1.0 equiv.); pinacolborane (80 µL, 0.55 mmol, 1.1 equiv.); reaction temperature: 70°C; catalyst (2.7 mg, 5.0 µmol, 1.0 mol%); 76 mg (64%, E/Z: 99/1) of a yellow oil

^{1}H NMR (400 MHz, $CDCl_3$): δ = 6.63 (dt, *J* = 18.0, 6.4 Hz, 1H), 5.42 (dt, *J* = 17.9, 1.6 Hz, 1H), 2.18 – 2.09 (m, 2H), 1.43 – 1.35 (m, 2H), 1.26 (s, 16H), 0.93 – 0.80 (m, 5H) ppm.

^{13}C{^{1}H} NMR (101 MHz, $CDCl_3$): δ = 155.0, 83.1, 36.0, 31.9, 29.1, 28.3, 24.9, 22.8, 14.2 ppm.

These spectroscopic data are in accordance with literature.[25]

4,4,5,5-Tetramethyl-2-[(1*E*)-5-methyl-1-hexen-1-yl]-1,3,2-dioxaborolane (5w)

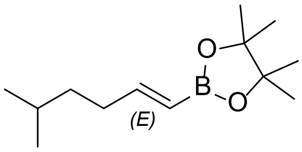

5-Methyl-1-hexyne (66 µL, 0.50 mmol, 1.0 equiv.); pinacolborane (80 µL, 0.55 mmol, 1.1 equiv.); reaction temperature: 70°C; catalyst (2.7 mg, 5.0 µmol, 1.0 mol%); 84 mg (75%, E/Z: 91/9) of a yellow oil

^{1}H NMR (400 MHz, $CDCl_3$): δ = 6.63 (dt, *J* = 17.9, 6.5 Hz, 1H), 5.42 (dt, *J* = 17.9, 1.6 Hz, 1H), 2.20 – 2.10 (m, 2H), 1.61 – 1.51 (m, 1H), 1.26 (s, 14H), 0.87 (d, *J* = 6.7 Hz, 6H) ppm.

^{13}C{^{1}H} NMR (101 MHz, $CDCl_3$): δ = 155.1, 83.1, 37.5, 33.8, 27.6, 25.0, 24.9, 22.6 ppm.

These spectroscopic data are in accordance with literature.[26]

4,4,5,5-Tetramethyl-2-[(1*E*)-3-phenyl-1-propen-1-yl]-1,3,2-dioxaborolane (5x)

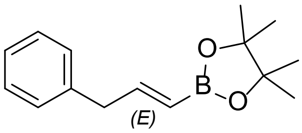

3-Phenyl-1-propyne (62 µL, 0.50 mmol, 1.0 equiv.); pinacolborane (80 µL, 0.55 mmol, 1.1 equiv.); reaction temperature: 70°C; catalyst (2.7 mg, 5.0 µmol, 1.0 mol%); 70 mg (57%, E/Z: 99/1) of a yellow oil

^{1}H NMR (400 MHz, $CDCl_3$): δ = 7.36 – 7.26 (m, 2H), 7.21 – 7.15 (m, 3H), 6.76 (dt, *J* = 17.8, 6.3 Hz, 1H), 5.45 (dt, *J* = 17.8, 1.7 Hz, 1H), 3.48 (dd, *J* = 6.3, 1.7 Hz, 2H), 1.25 (s, 12H) ppm.

^{13}C{^{1}H} NMR (101 MHz, $CDCl_3$): δ = 152.6, 139.2, 129.1, 128.6, 126.3, 83.3, 42.4, 24.9 ppm.

These spectroscopic data are in accordance with literature.[27]

References

(1) Zobernig, D. P.; Luxner, M.; Stöger, B.; Veiros, L. F.; Kirchner, K. Hydrogenation of Terminal Alkenes Catalyzed by Air-Stable Mn(I) Complexes Bearing an N-Heterocyclic Carbene-Based PCP Pincer Ligand. *Chem. Eur. J.* **2024**, *30*, e202302455.

(2) Zhang, X.; Xie, X.; Liu, Y. Nickel-Catalyzed Highly Regioselective Hydrocyanation of Terminal Alkynes with $Zn(CN)_2$ Using Water as the Hydrogen Source. *J. Am. Chem. Soc.* **2018**, *140*, 7385–7389.

(3) N. Miyaura, K. Yamada, A. Suzuki, *Tetrahedron Lett.* **1979**, *20*, 3437–3440.

(4) Bruker computer programs: APEX3, SAINT and SADABS (Bruker AXS Inc., Madison, WI, 2020).

(5) G. M. Sheldrick, *Acta Crystallogr.* **2015**, *A71*, 3–8.

(6) G. M. Sheldrick, *Acta Crystallogr.* **2015**, *C71*, 3–8

(7) C. F. Macrae, P. R. Edgington, P. McCabe, E. Pidcock, G. P. Shields, R. Taylor, M. Towler and J. van de Streek, *J. Appl. Cryst.* **2006**, *39*, 453–457.

(8) Gaussian 09, Revision **A.01**, Frisch, M. J.; Trucks, G. W.; Schlegel, H. B.; Scuseria, G. E.; Robb, M. A.; Cheeseman, J. R.; Scalmani, G.; Barone, V.; Mennucci, B.; Petersson, G. A.; Nakatsuji, H.; Caricato, M.; Li, X.; Hratchian, H. P.; Izmaylov, A. F.; Bloino, J.; Zheng, G.; Sonnenberg, J. L.; Hada, M.; Ehara, M.; Toyota, K.; Fukuda, R.; Hasegawa, J.; Ishida, M.; Nakajima, T.; Honda, Y.; Kitao, O.; Nakai, H.; Vreven, T.; Montgomery, Jr., J. A.; Peralta, J. E.; Ogliaro, F.; Bearpark, M.; Heyd, J. J.; Brothers, E.; Kudin, K. N.; Staroverov, V. N.; Kobayashi, R.; Normand, J.; Raghavachari, K.; Rendell, A.; Burant, J. C.; Iyengar, S. S.; Tomasi, J.; Cossi, M.; Rega, N.; Millam, J. M.; Klene, M.; Knox, J. E.; Cross, J. B.; Bakken, V.; Adamo, C.; Jaramillo, J.; Gomperts, R.; Stratmann, R. E.; Yazyev, O.; Austin, A. J.; Cammi, R.; Pomelli, C.; Ochterski, J. W.; Martin, R. L.; Morokuma, K.; Zakrzewski, V. G.; Voth, G. A.; Salvador, P.; Dannenberg, J. J.; Dapprich, S.; Daniels, A. D.; Farkas, Ö.; Foresman, J. B.; Ortiz, J. V.; Cioslowski, J.; Fox, D. J. Gaussian, Inc., Wallingford CT, 2009.

(9) Hehre, W. J.; Radom, L.; Schleyer, P. v.R.; Pople, J. A. *Ab Initio Molecular Orbital Theory*, John Wiley & Sons, NY, 1986.

(10) Parr, R. G.; Yang, W. *Density Functional Theory of Atoms and Molecules*; Oxford University Press: New York, 1989.

(11) (a) Perdew, J. P.; Burke, K.; Ernzerhof, M. Generalized Gradient Approximation Made Simple *Phys. Rev. Lett.* **1996**, *77*, 3865-3868; (b) Perdew, J. P.; Burke, K.; Ernzerhof, M. Generalized Gradient Approximation Made Simple *Phys. Rev. Lett.* **1997**, *78*, 1396-1396. (c) Perdew, J. P. Density-functional approximation for the correlation energy of the inhomogeneous electron gas *Phys. Rev. B* **1986**, *33*, 8822-8824.

(12) (a) Haeusermann, U.; Dolg, M.; Stoll, H.; Preuss, H.; Schwerdtfeger, P.; Pitzer, R. M. Accuracy of energy-adjusted quasirelativistic ab initio pseudopotentials *Mol. Phys.* **1993**, *78*, 1211-1224. (b) Kuechle, W.; Dolg, M.; Stoll, H.; Preuss, H. Energy-adjusted pseudopotentials for the actinides. Parameter sets and test calculations for thorium and thorium monoxide *J. Chem. Phys.* **1994**, *100*, 7535-7542. (c) Leininger, T.; Nicklass, A.; Stoll, H.; Dolg, M.; Schwerdtfeger, P. The accuracy of the pseudopotential approximation. II. A comparison of various core sizes for indium pseudopotentials in calculations for spectroscopic constants of InH, InF, and InCl *J. Chem. Phys.* **1996**, *105*, 1052-1059.

(13) (a) Ditchfield, R.; Hehre, W. J.; Pople, J. A. Self-Consistent Molecular-Orbital Methods. IX. An Extended Gaussian-Type Basis for Molecular-Orbital Studies of Organic Molecules *J. Chem. Phys.* **1971**, *54*, 724-728. (b) Hehre, W. J.; Ditchfield, R.; Pople, J. A. Self-Consistent Molecular Orbital Methods. 12. Further extensions of Gaussian-type basis sets for use in molecular-orbital studies of organic-molecules *J. Chem. Phys.* **1972**, *56*, 2257-2261. (c) Hariharan, P. C.; Pople, J. A. Accuracy of AH equilibrium geometries by single determinant molecular-orbital theory *Mol. Phys.* **1974**, *27*, 209-214. (d) Gordon, M. S. The isomers of silacyclopropane *Chem. Phys. Lett.* **1980**, *76*, 163-168. (e) Hariharan, P. C.; Pople, J. A. Influence of polarization functions on molecular-orbital hydrogenation energies *Theor. Chim. Acta* **1973**, *28*, 213-222.

(14) (a) Peng, C.; Ayala, P. Y.; Schlegel, H. B.; Frisch, M. J. Using redundant internal coordinates to optimize equilibrium geometries and transition states *J. Comp. Chem.* **1996**, *17*, 49-56. (b) Peng, C.; Schlegel, H. B. Combining Synchronous Transit and Quasi-Newton Methods for Finding Transition States *Israel J. Chem.* **1993**, *33*, 449-454.

(15) (a) McClean, A. D.; Chandler, G. S. Contracted Gaussian basis sets for molecular calculations. I. Second row atoms, Z=11-18 *J. Chem. Phys.* **1980**, *72*, 5639-5648. (b) Krishnan, R.; Binkley, J. S.; Seeger, R.; Pople, J. A. Self-consistent molecular orbital methods. XX. A basis set for correlated wave functions *J. Chem. Phys.* **1980**, *72*, 650-654. (c) Wachters, A. J. H. Gaussian Basis Set for Molecular Wavefunctions Containing Third-Row Atoms *J. Chem. Phys.* **1970**, *52*, 1033-1036. (d) Hay, P. J. Gaussian basis sets for molecular

calculations - representation of 3D orbitals in transition-metal atoms *J. Chem. Phys.* **1977**, *66*, 4377-4384. (e) Raghavachari, K.; Trucks, G. W. Highly correlated systems: Excitation energies of first row transition metals Sc-Cu *J. Chem. Phys.* **1989**, *91*, 1062-1065. (f) Binning Jr., R. C.; Curtiss, L. A. Compact contracted basis-sets for 3rd-row atoms - Ga-Kr *J. Comp. Chem.* **1990**, *11*, 1206-1216. (g) McGrath, M. P.; Radom, L. Extension of Gaussian-1 (G1) theory to bromine-containing molecules *J. Chem. Phys.* **1991**, *94*, 511-516. (h) Curtiss, L. A.; McGrath, M. P.; Blaudeau, J.-P.; Davis, N. E.; Binning Jr., R. C.; Radom, L. Extension of Gaussian-2 theory to molecules containing third-row atoms Ga-Kr *J. Chem. Phys.*, **1995**, *103*, 6104-6113. (i) Clark, T.; Chandrasekhar, J.; Spitznagel, G. W.; Schleyer, P. v. R. Efficient diffuse function-augmented basis-sets for anion calculations. 3. The 3-21+G basis set for 1st-row elements, Li-F *J. Comp. Chem.* **1983**, *4*, 294-301. (j) Frisch, M. J.; Pople, J. A.; Binkley, J. S. Self-Consistent Molecular Orbital Methods. 25. Supplementary Functions for Gaussian Basis Sets *J. Chem. Phys.* **1984**, *80*, 3265-3269.

(16) Grimme, S.; Antony, J.; Ehrlich, S.; Krieg, H. A consistent and accurate ab initio parameterization of density functional dispersion correction (DFT-D) for the 94 elements H-Pu *J. Chem. Phys.* **2010**, *132*, 154104.

(17) (a) Becke, A. D.; Johnson, E. R. A density-functional model of the dispersion interaction *J. Chem. Phys.* **2005**, *122*, 154101. (b) Johnson, E. R.; Becke, A. D. A post-Hartree-Fock model of intermolecular interactions *J. Chem. Phys.* **2005**, *123*, 24101. (c) Johnson, E. R.; Becke, A. D. A post-Hartree-Fock model of intermolecular interactions: Inclusion of higher-order corrections *J. Chem. Phys.* **2006**, *124*, 174104.

(18) Obligacion, J. V; Neely, J. M.; Yazdani, A. N.; Pappas, I.; Chirik, P. J. Cobalt Catalyzed Z-Selective Hydroboration of Terminal Alkynes and Elucidation of the Origin of Selectivity. *J. Am. Chem. Soc.* **2015**, *137*, 5855–5858.

(19) Gorgas, N.; Alves, L. G.; Stöger, B.; Martins, A. M.; Veiros, L. F.; Kirchner, K. Stable, Yet Highly Reactive Nonclassical Iron(II) Polyhydride Pincer Complexes: Z-Selective Dimerization and Hydroboration of Terminal Alkynes. *J. Am. Chem. Soc.* **2017**, *139*, 8130–8133.

(20) Polášek, J.; Paciorek, J.; Stošek, J.; Semrád, H.; Munzarová, M.; Mazal, C. Stereoselective Bromoboration of Acetylene with Boron Tribromide: Preparation and Cross-Coupling Reactions of (Z)-Bromovinylboronates. *J. Org. Chem.* **2020**, *85*, 6992–7000.

(21) Garhwal, S.; Fridman, N.; de Ruiter, G. Z-Selective Alkyne Functionalization Catalyzed by a Trans-Dihydride N-Heterocyclic Carbene (NHC) Iron Complex. *Inorg. Chem.* **2020**, *59*, 13817–13821.

(22) Ohmura, T.; Yamamoto, Y.; Miyaura, N. Rhodium- or Iridium-Catalyzed Trans-Hydroboration of Terminal Alkynes, Giving (Z)-1-Alkenylboron Compounds. *J. Am. Chem. Soc.* **2000**, *122*, 4990–4991.

(23) Lyu, Y.; Toriumi, N.; Iwasawa, N. (Z)-Selective Hydroboration of Terminal Alkynes Catalyzed by a PSP–Pincer Rhodium Complex. *Org. Lett.* **2021**, *23*, 9262–9266.

(24) Yang, Z.; Zhong, M.; Ma, X.; Nijesh, K.; De, S.; Parameswaran, P.; Roesky, H. W. An Aluminum Dihydride Working as a Catalyst in Hydroboration and Dehydrocoupling. *J. Am. Chem. Soc.* **2016**, *138*, 2548–2551.

(25) Bismuto, A.; Thomas, S. P.; Cowley, M. J. Aluminum Hydride Catalyzed Hydroboration of Alkynes. *Angew. Chem., Int. Ed.* **2016**, *55*, 15356–15359.

(26) Ben-Daat, H.; Rock, C. L.; Flores, M.; Groy, T. L.; Bowman, A. C.; Trovitch, R. J. Hydroboration of Alkynes and Nitriles Using an α-Diimine Cobalt Hydride Catalyst. *Chem. Commun.* **2017**, *53*, 7333–7336.

(27) Hemelaere, R.; Carreaux, F.; Carboni, B. Synthesis of Alkenyl Boronates from Allyl-Substituted Aromatics Using an Olefin Cross-Metathesis Protocol. *J. Org. Chem.* **2013**, *78*, 6786–6792.

NMR and IR Spectra

Complexes

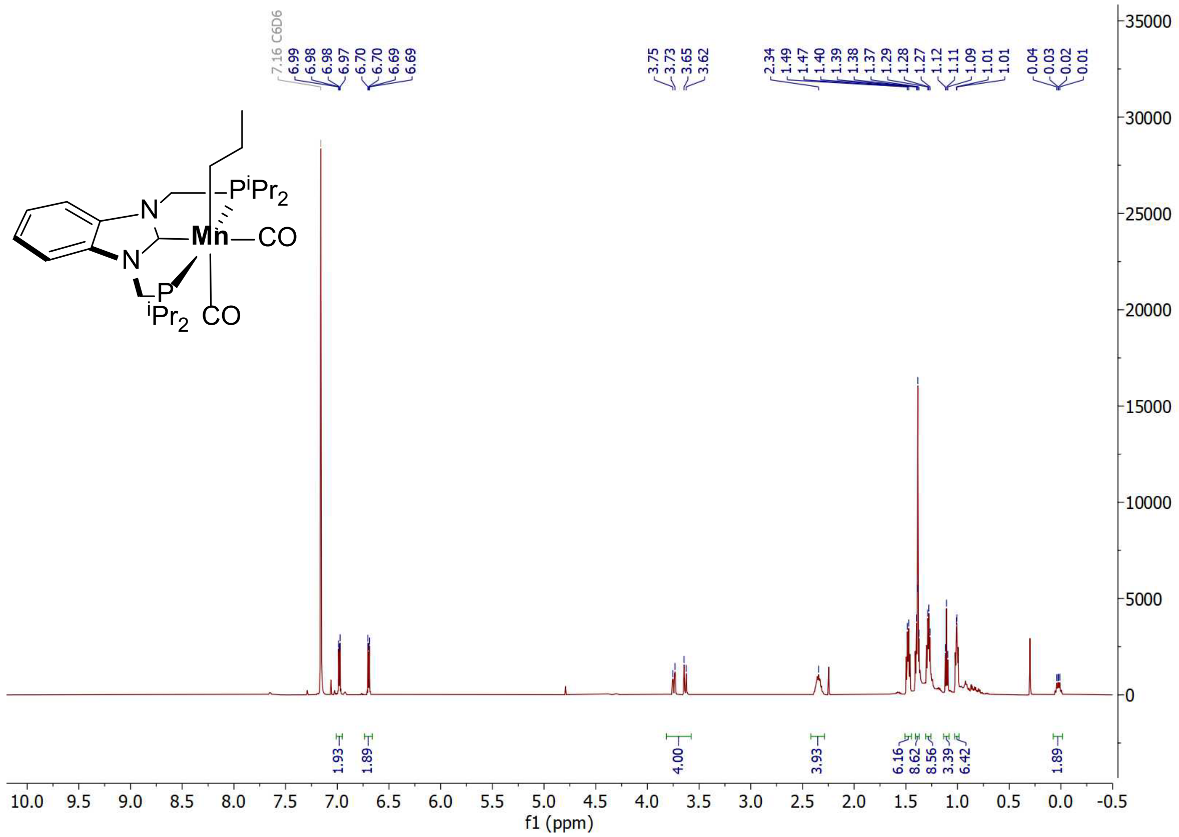

Figure S15. **^{1}H-NMR spectrum of complex 3**

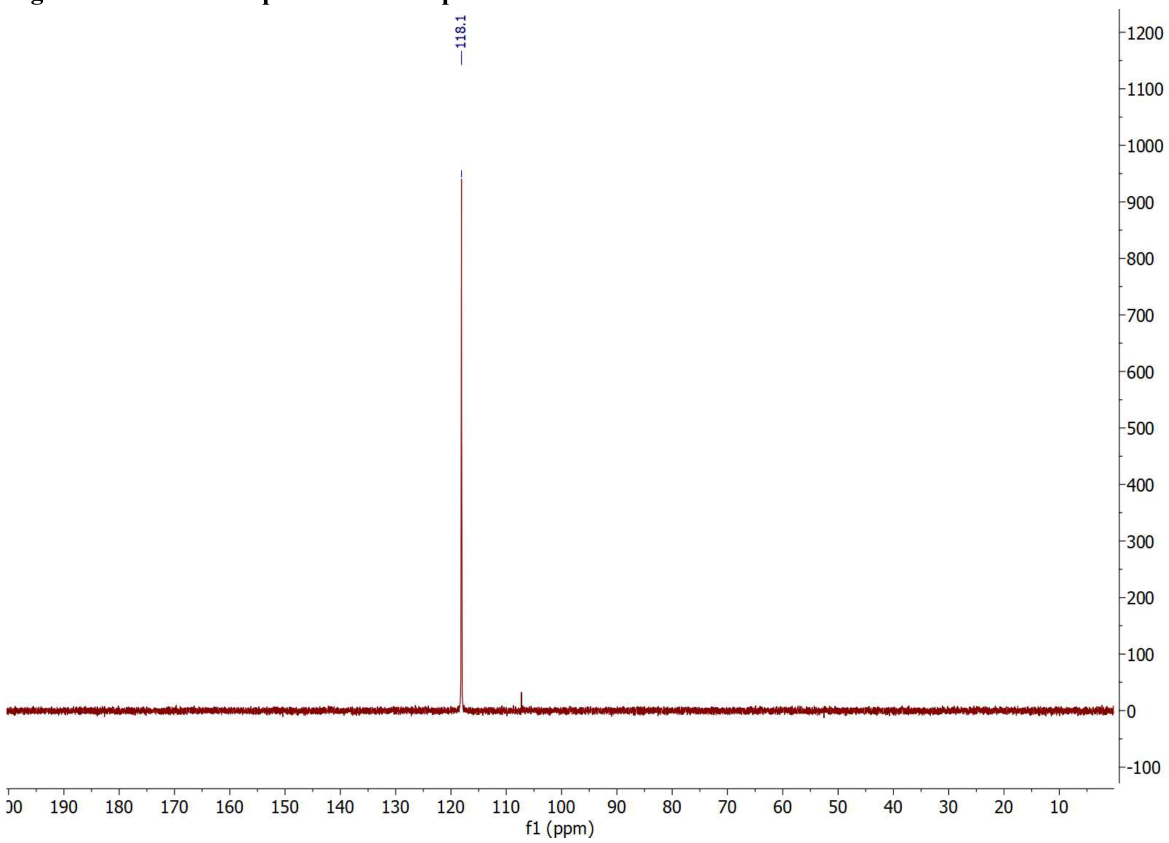

Figure S16. **^{31}P{^{1}H}-NMR spectrum of complex 3**

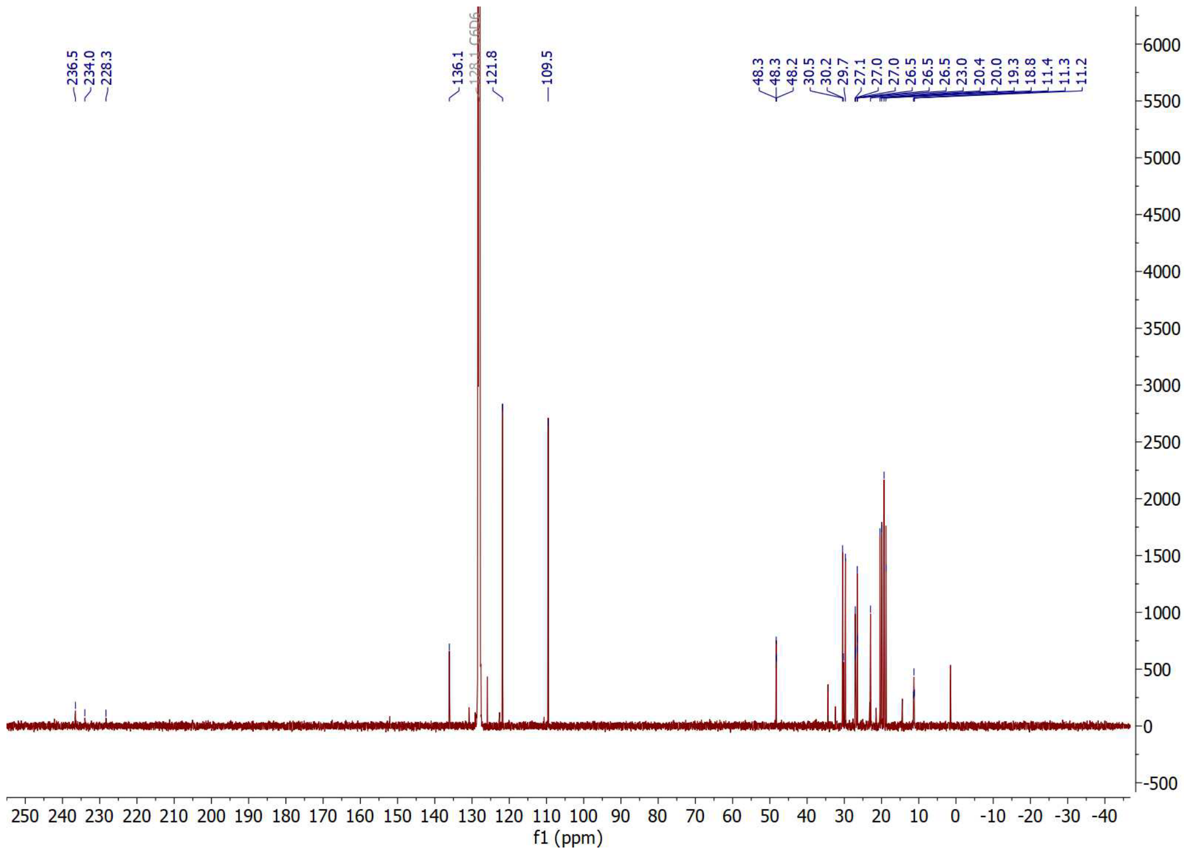

Figure S17. $^{13}C\{^{1}H\}$**-NMR spectrum of complex 3**

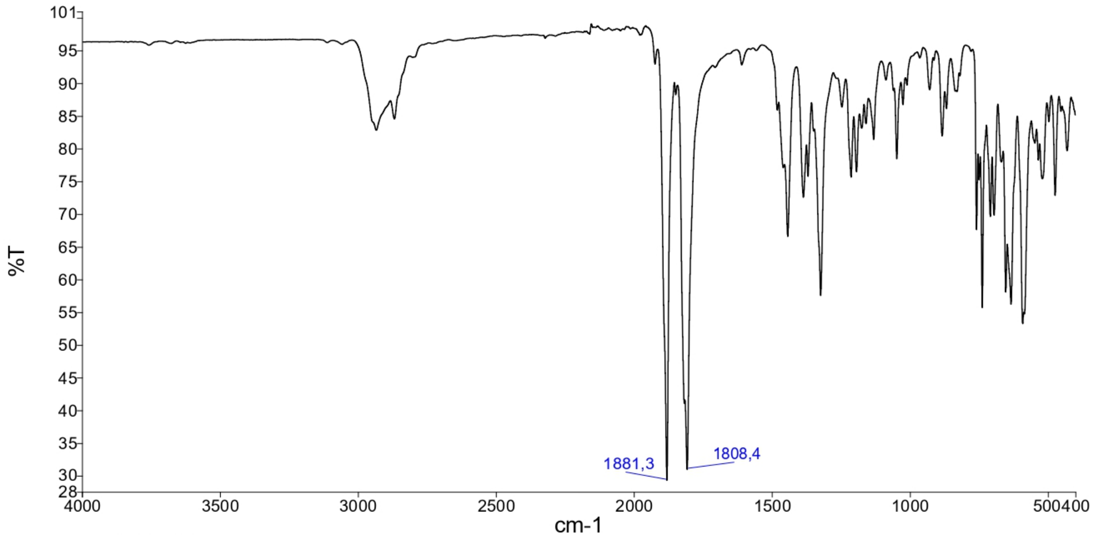

Figure S18. IR spectrum of complex 3

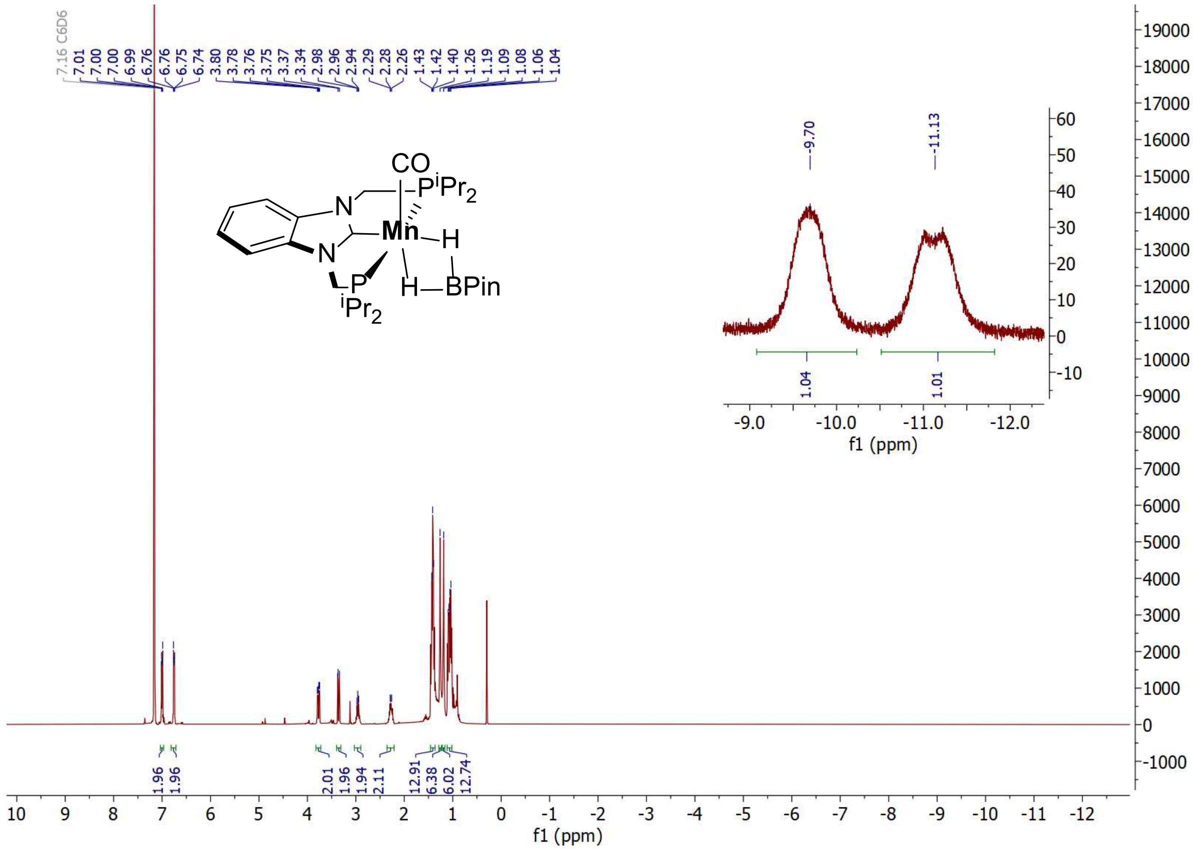

Figure S19. ^{1}**H-NMR spectrum of complex 4**

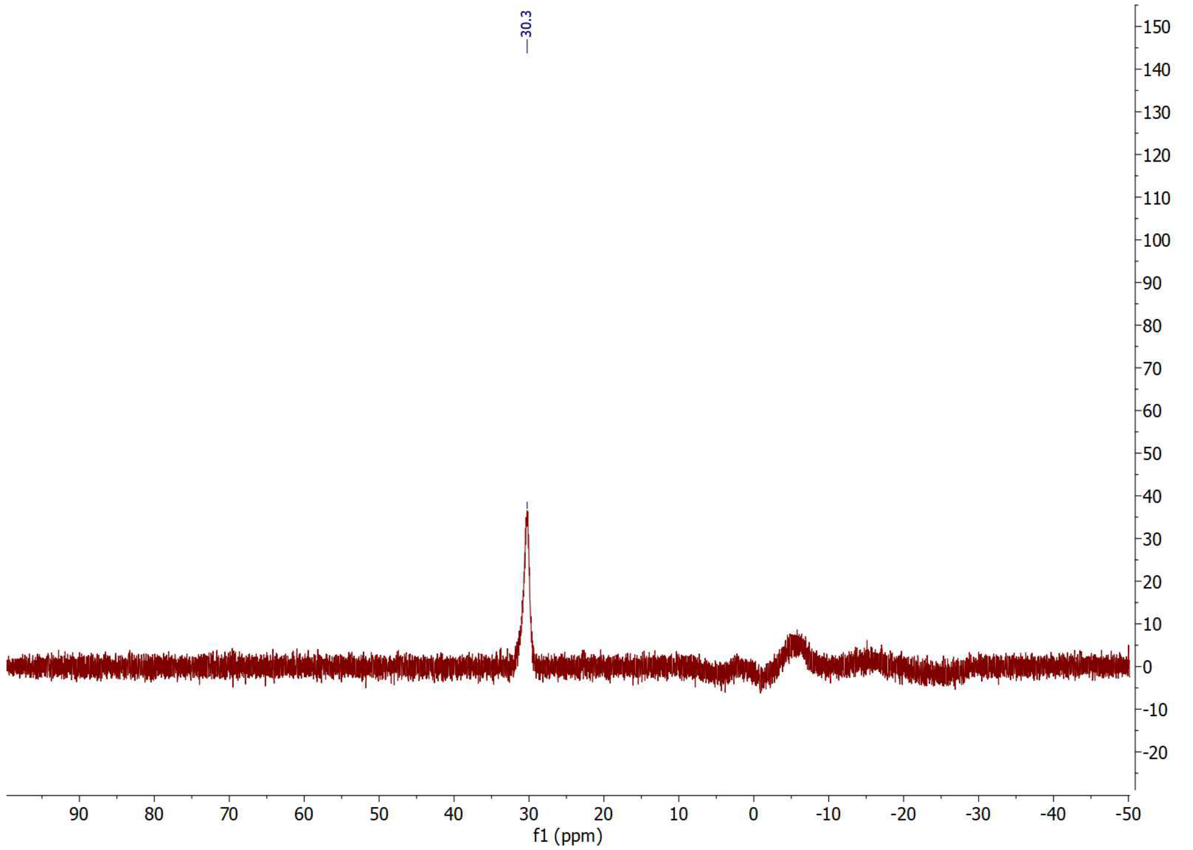

Figure S20. ^{11}**B{**^{1}**H}-NMR spectrum of complex 4**

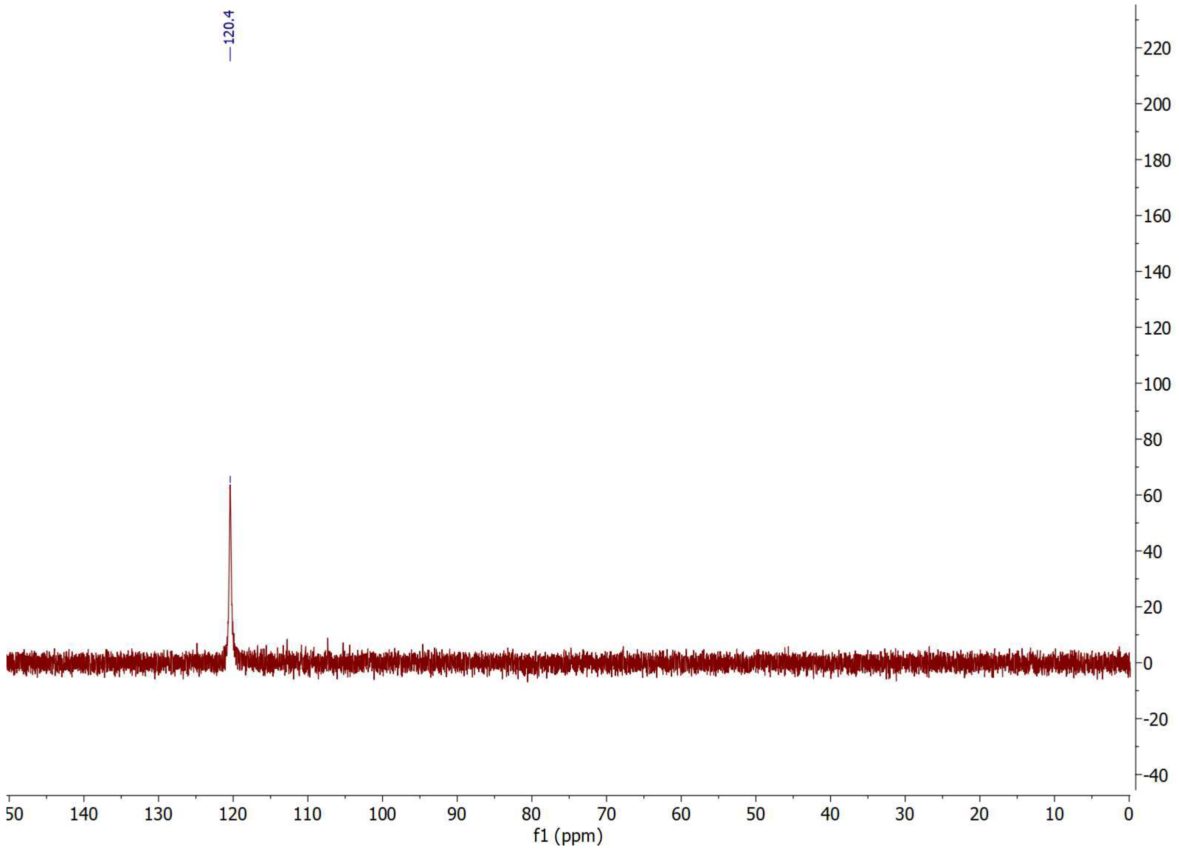

Figure S21. $^{31}P\{^{1}H\}$**-NMR spectrum of complex 4**

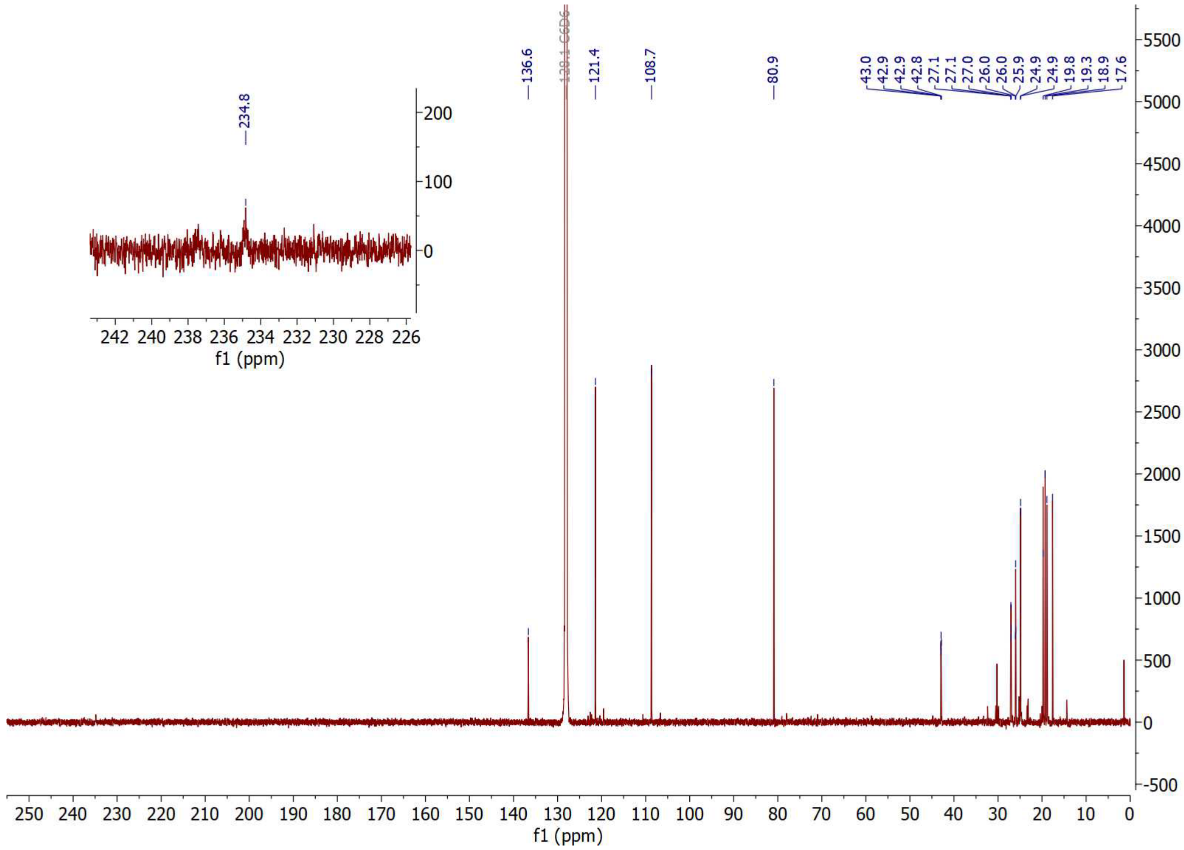

Figure S22. $^{13}C\{^{1}H\}$**-NMR spectrum of complex 4**

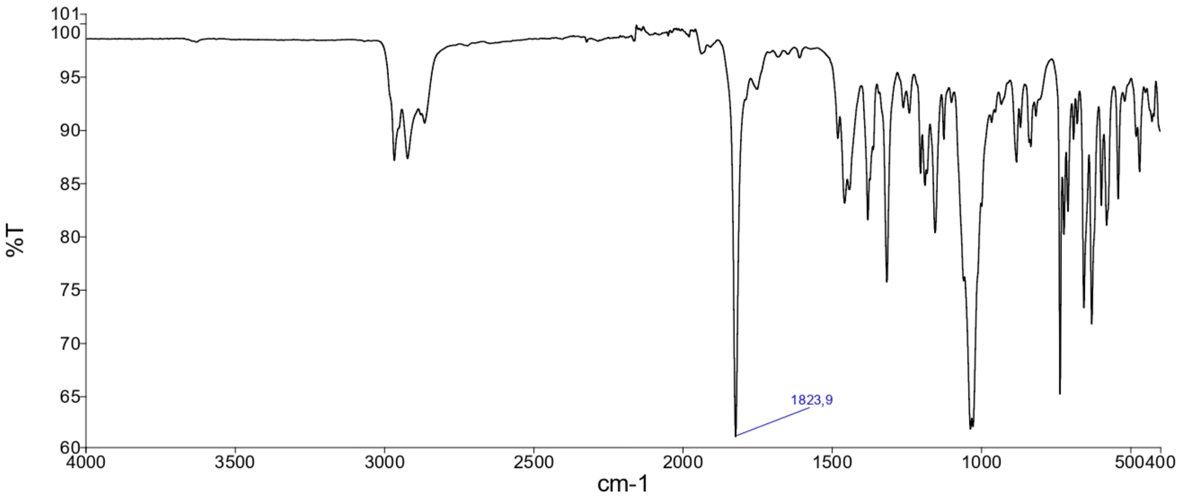

Figure S23. IR spectrum of complex 4

Organic Compounds

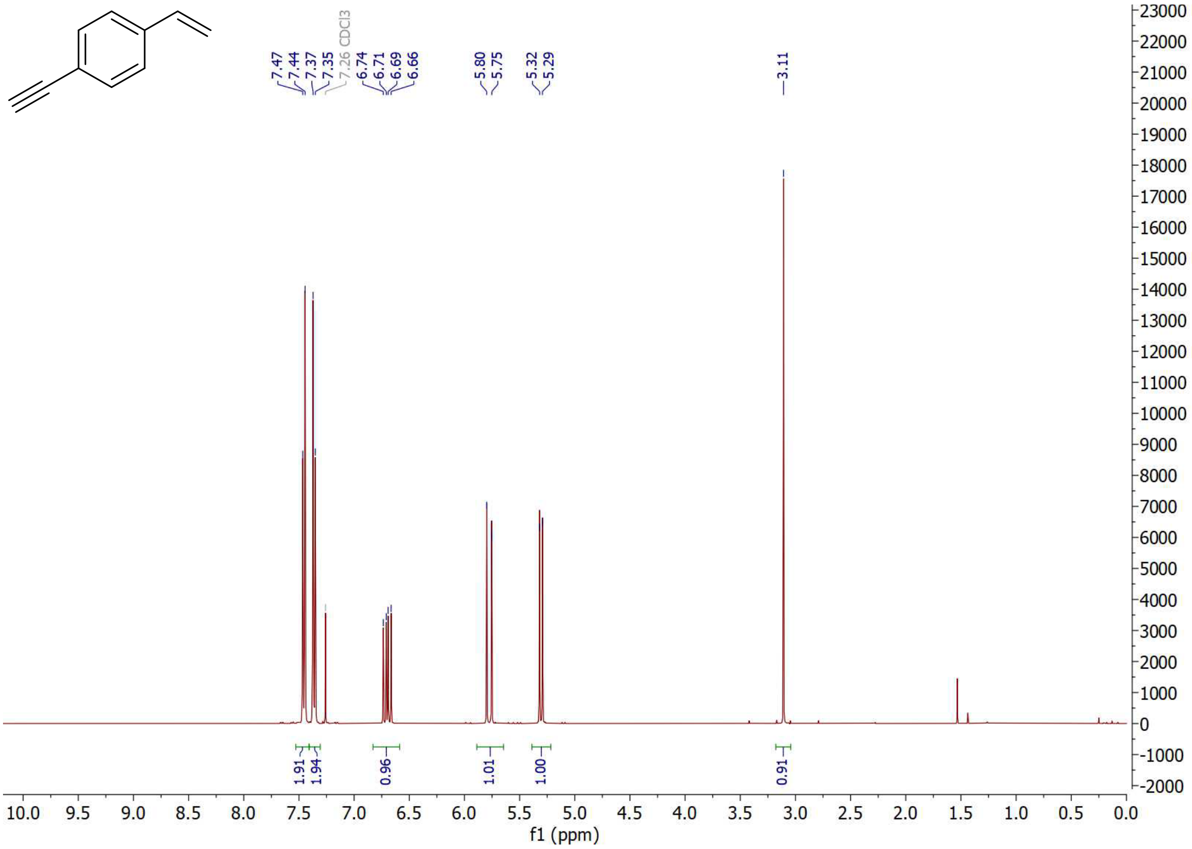

Figure S24. ^{1}**H-NMR spectrum of 4-ethynylstyrene**

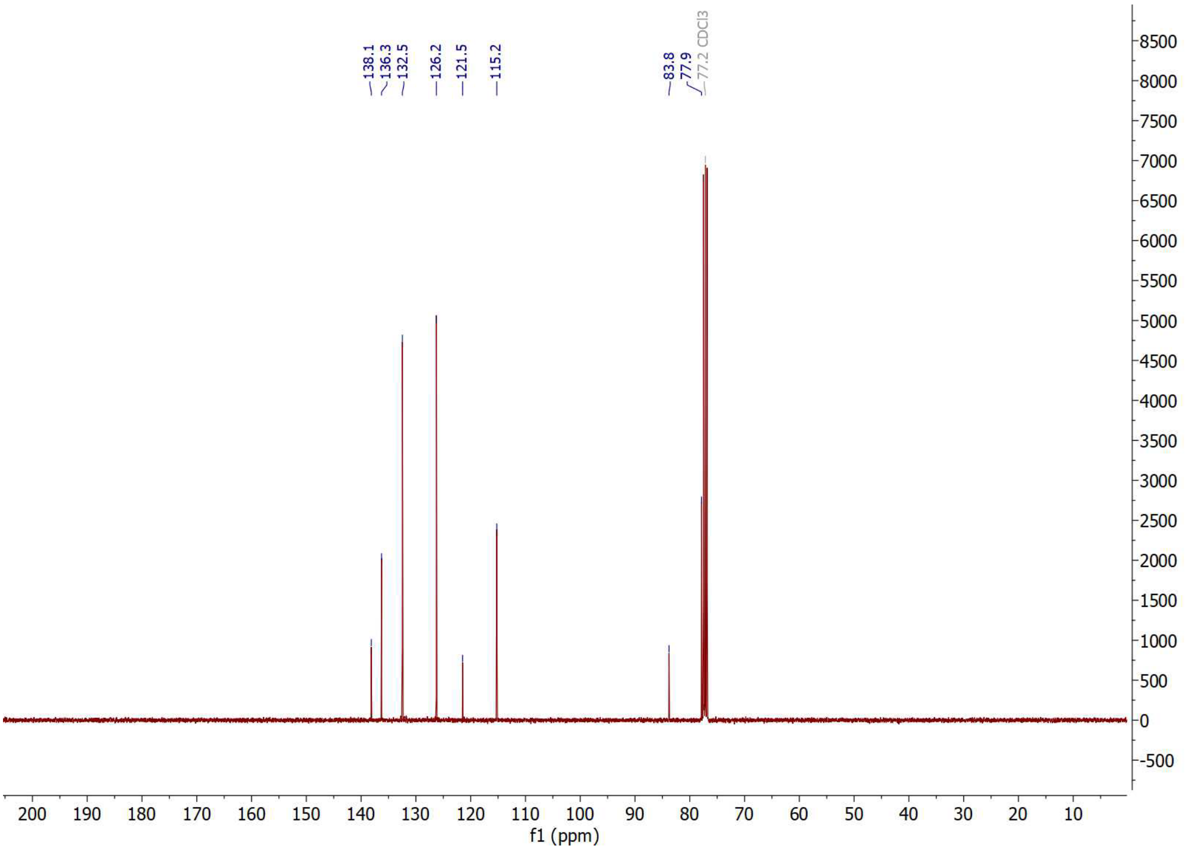

Figure S25. ^{13}**C{**^{1}**H}-NMR spectrum of 4-ethynylstyrene**

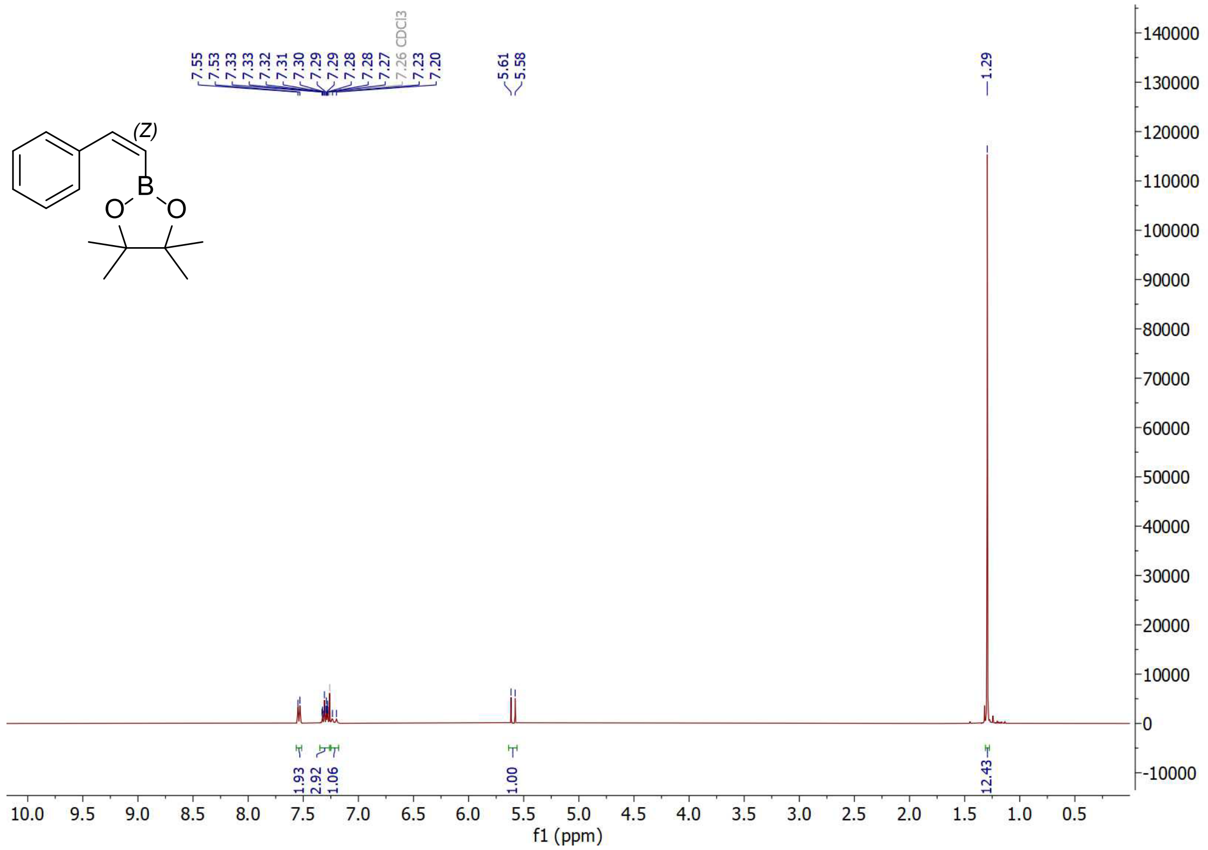

Figure S26. ^{1}H-NMR spectrum of substrate 5a

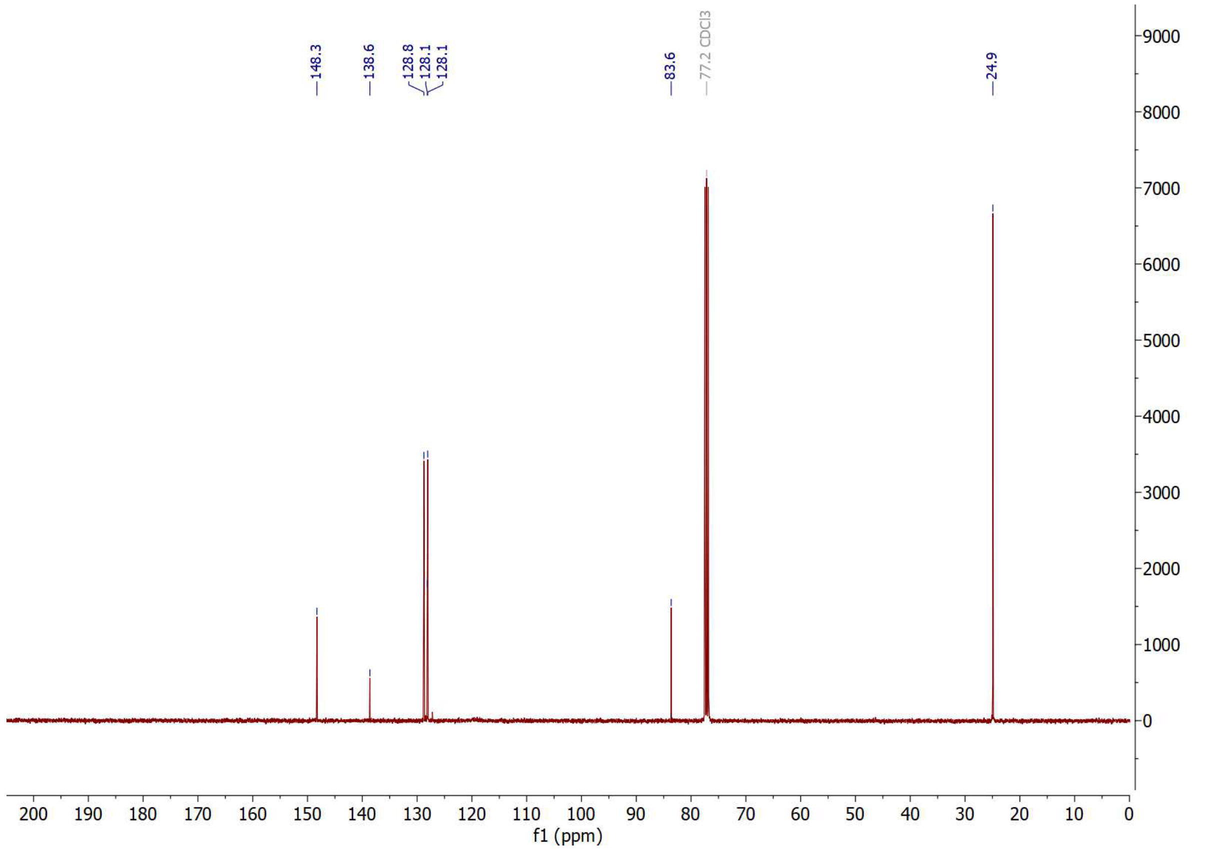

Figure S27. $^{13}C\{^{1}H\}$-NMR spectrum of substrate 5a

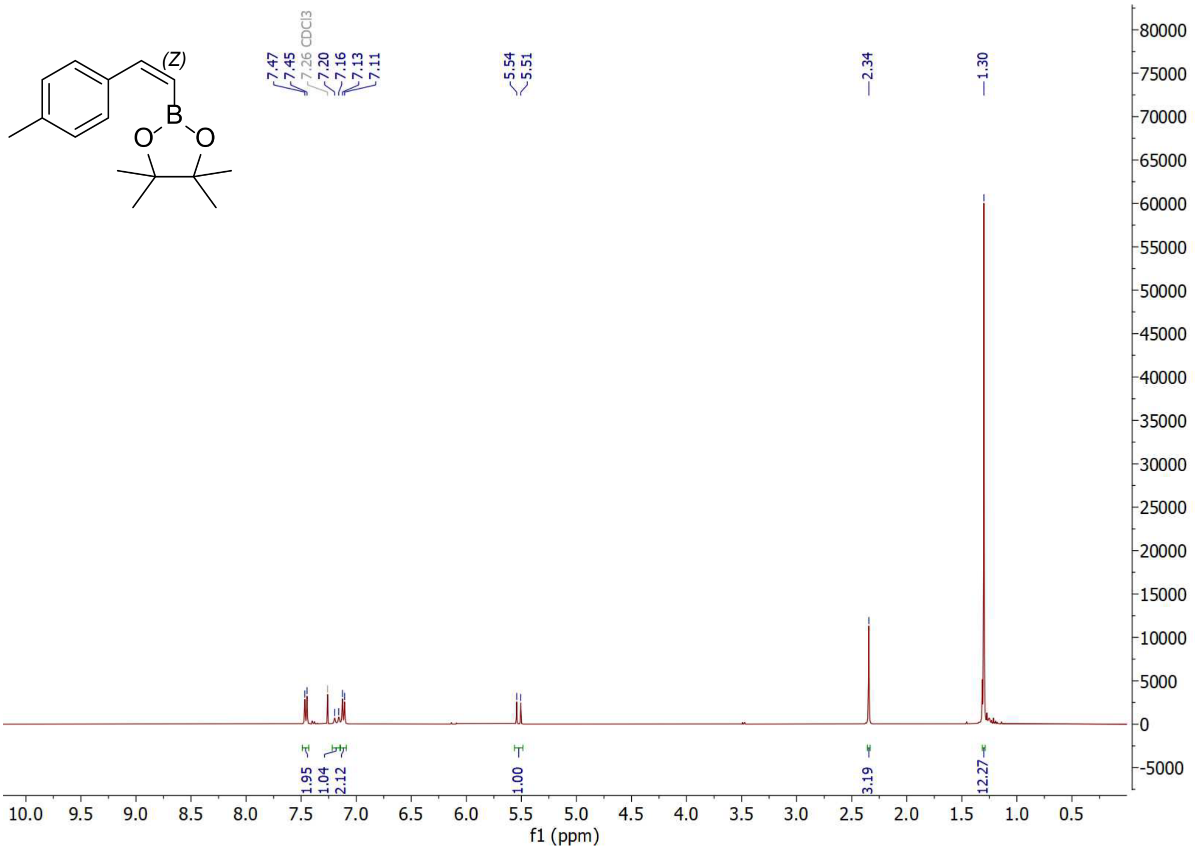

Figure S28. ^{1}H-NMR spectrum of substrate 5b

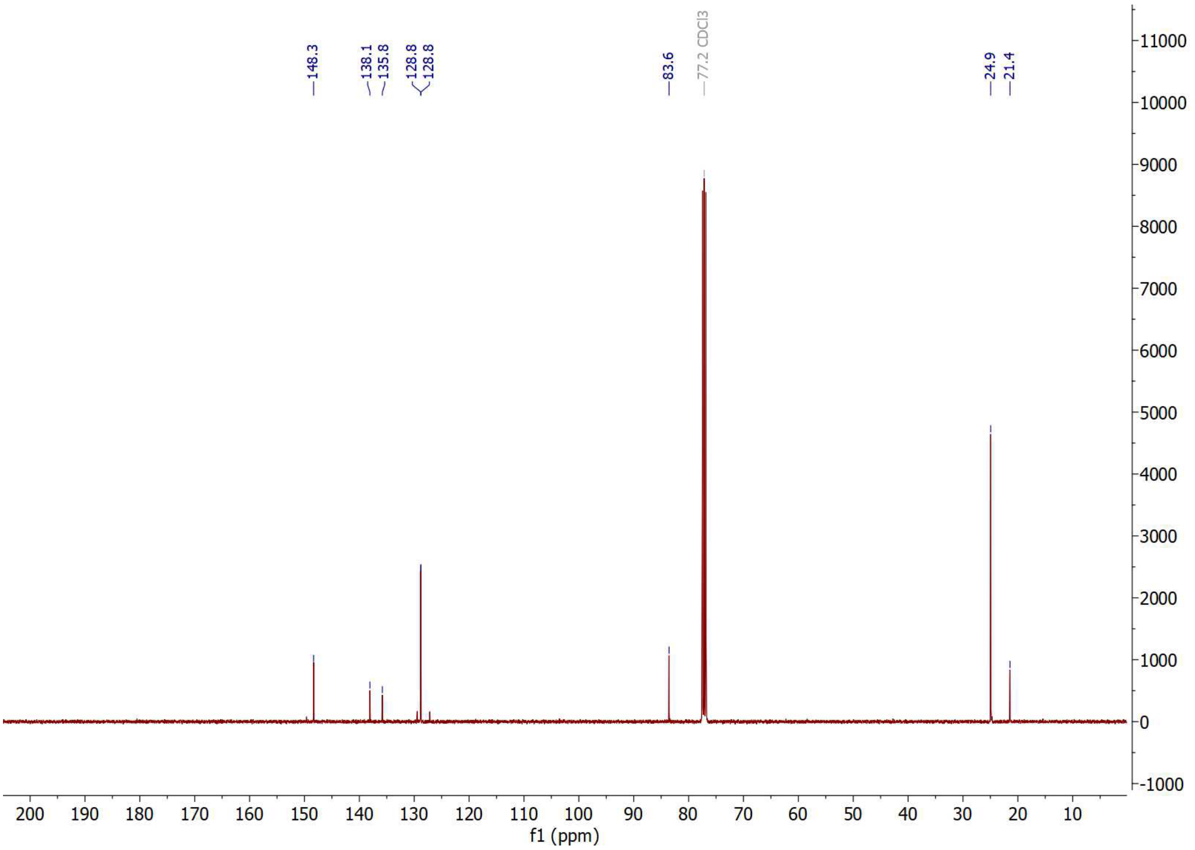

Figure S29. ^{13}C{^{1}H}-NMR spectrum of substrate 5b

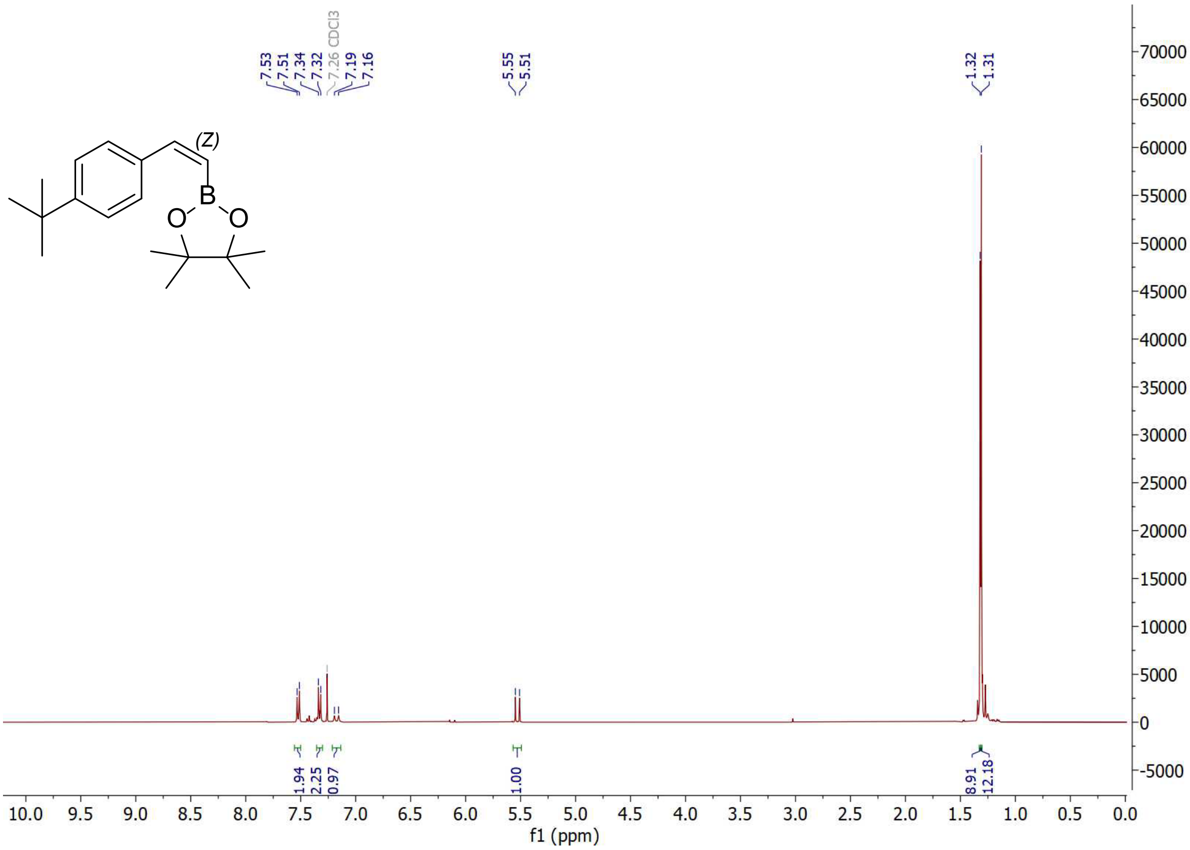

Figure S30. ^{1}H-NMR spectrum of substrate 5c

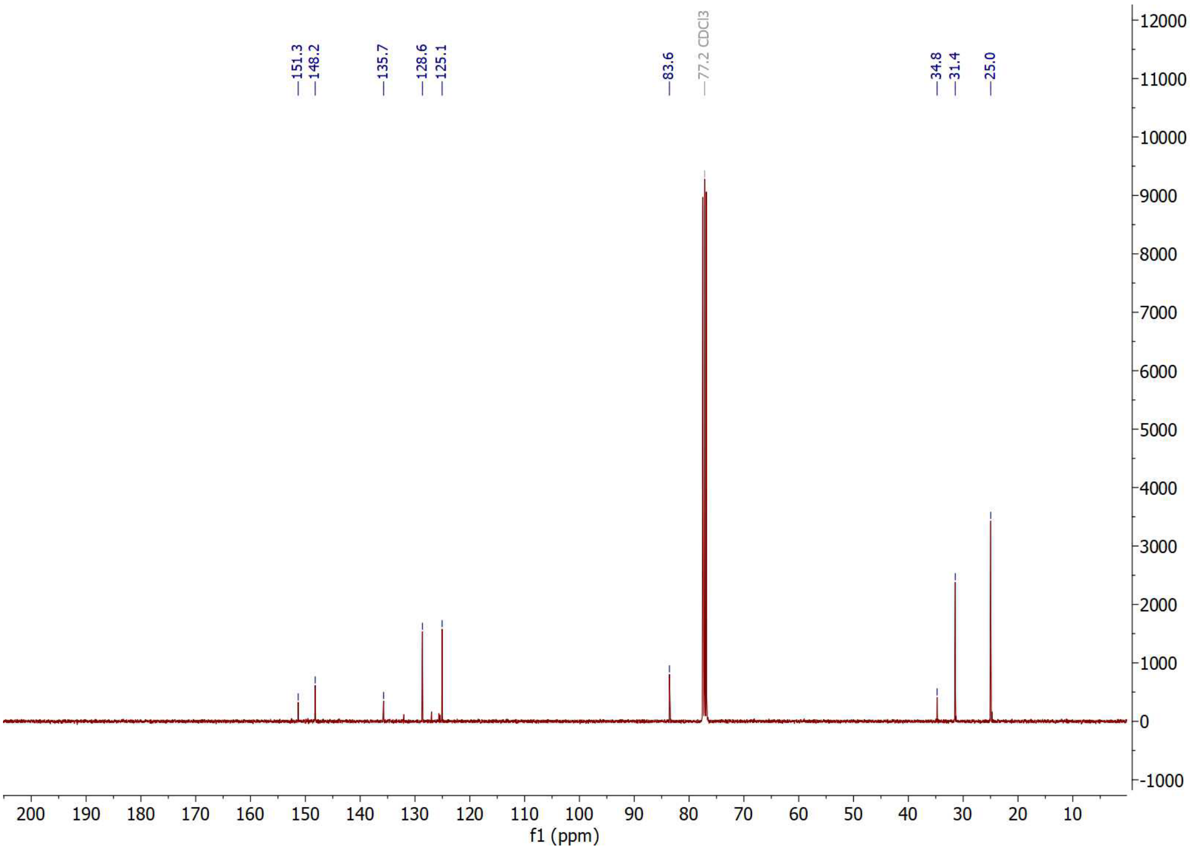

Figure S31. $^{13}C\{^{1}H\}$-NMR spectrum of substrate 5c

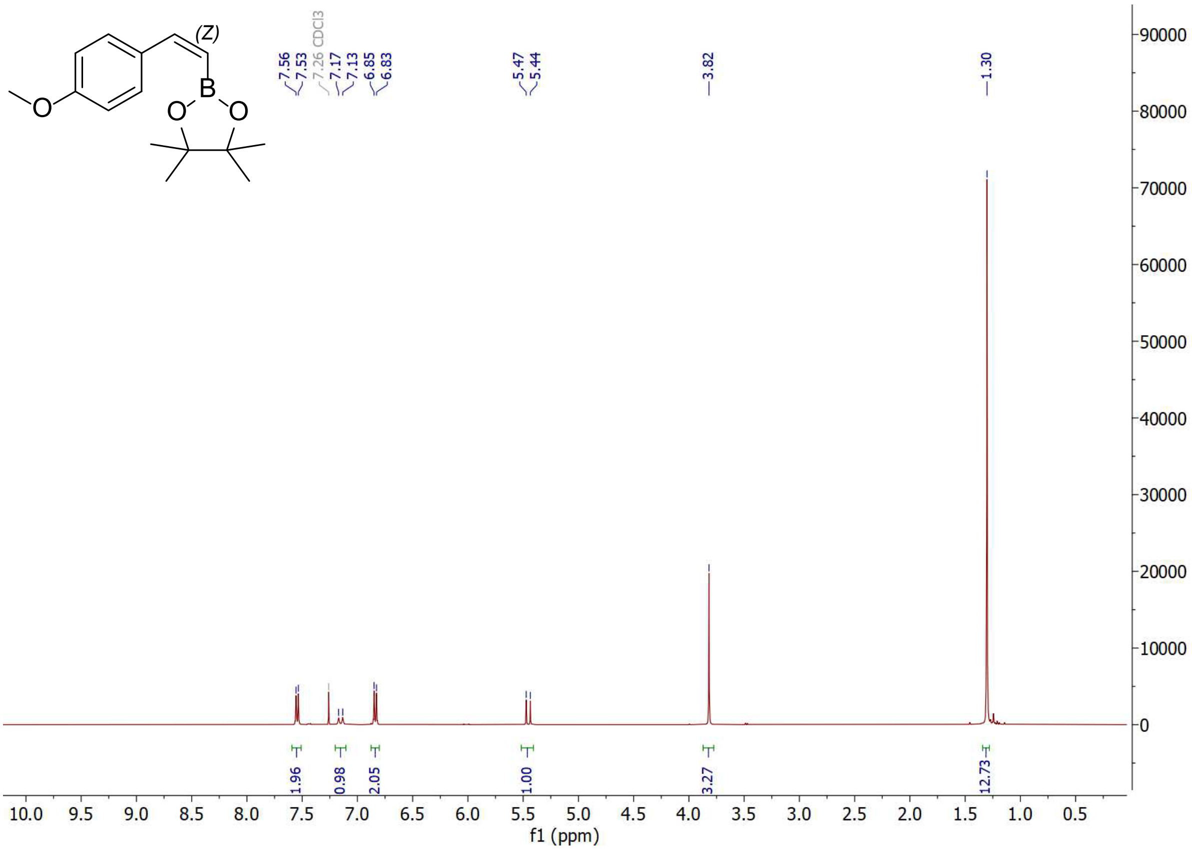

Figure S32. **^{1}H-NMR spectrum of substrate 5d**

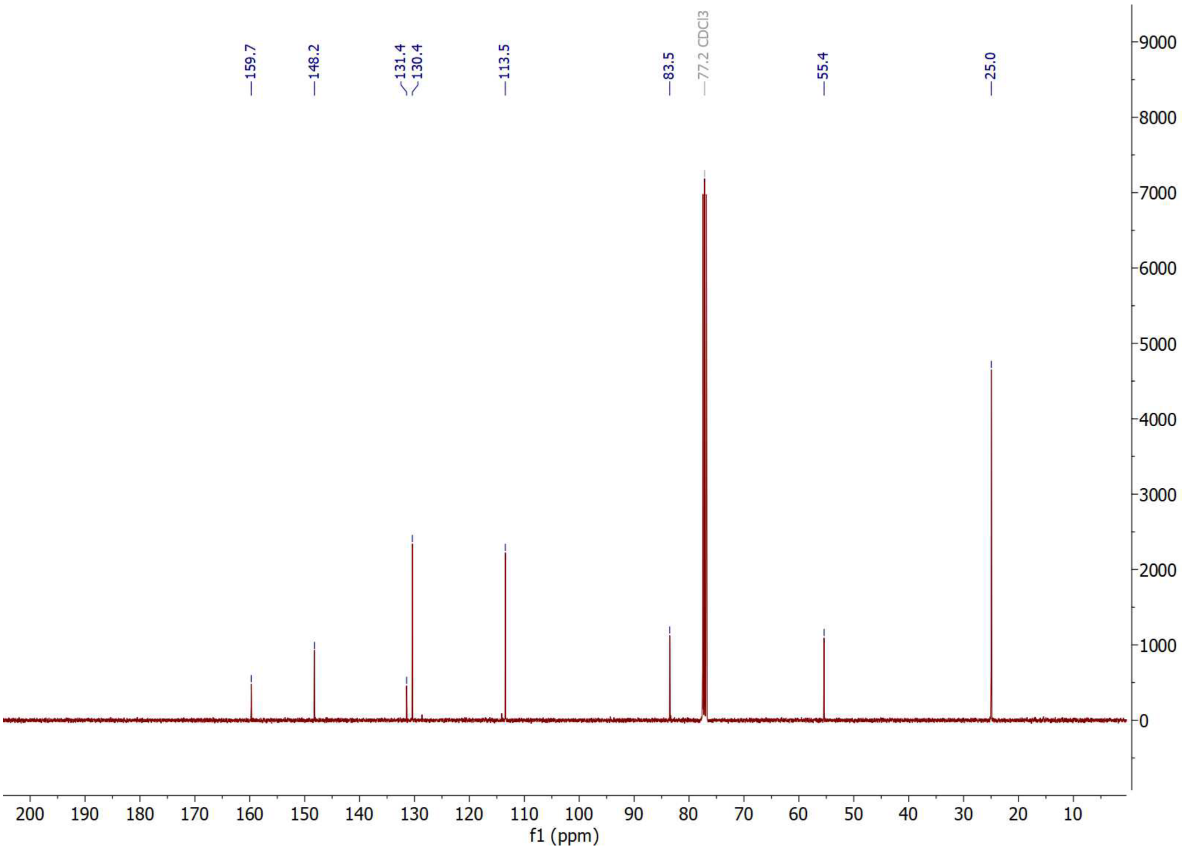

Figure S33. **^{13}C{^{1}H}-NMR spectrum of substrate 5d**

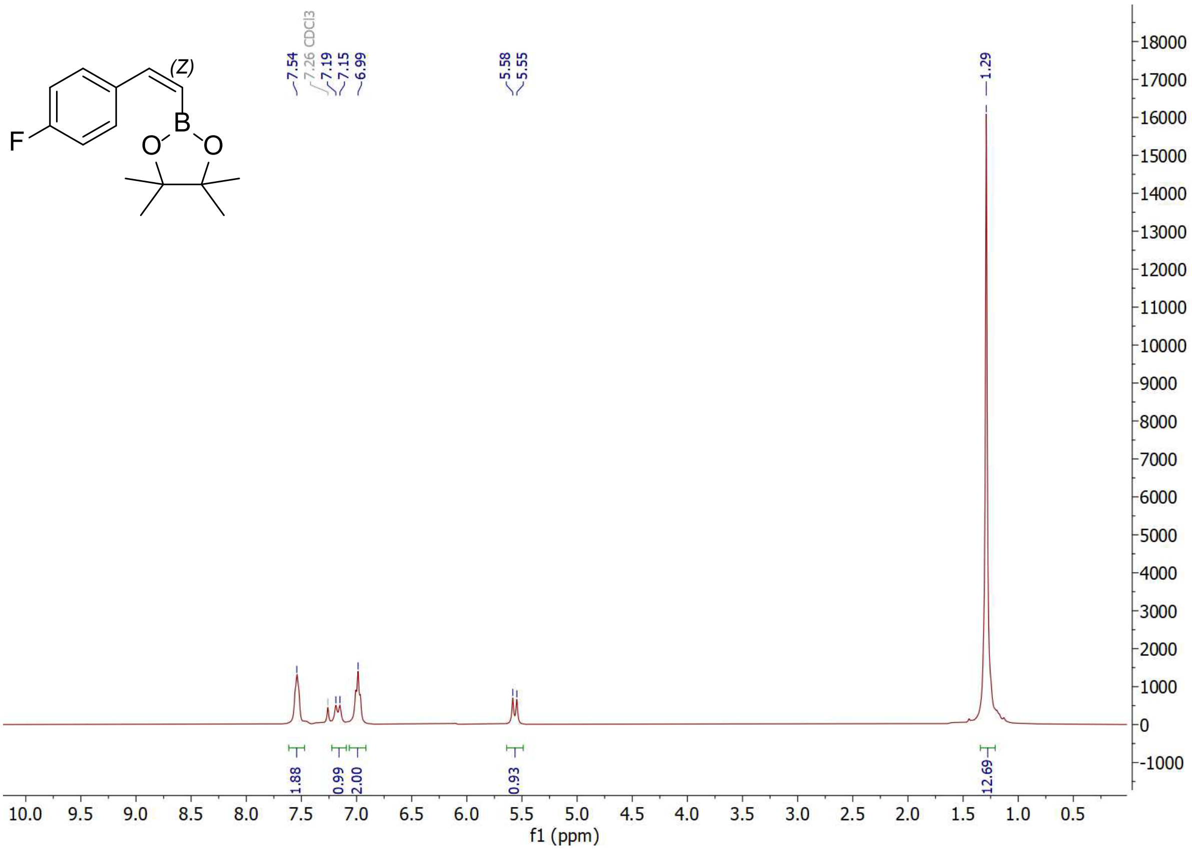

Figure S34. **^{1}H-NMR spectrum of substrate 5e**

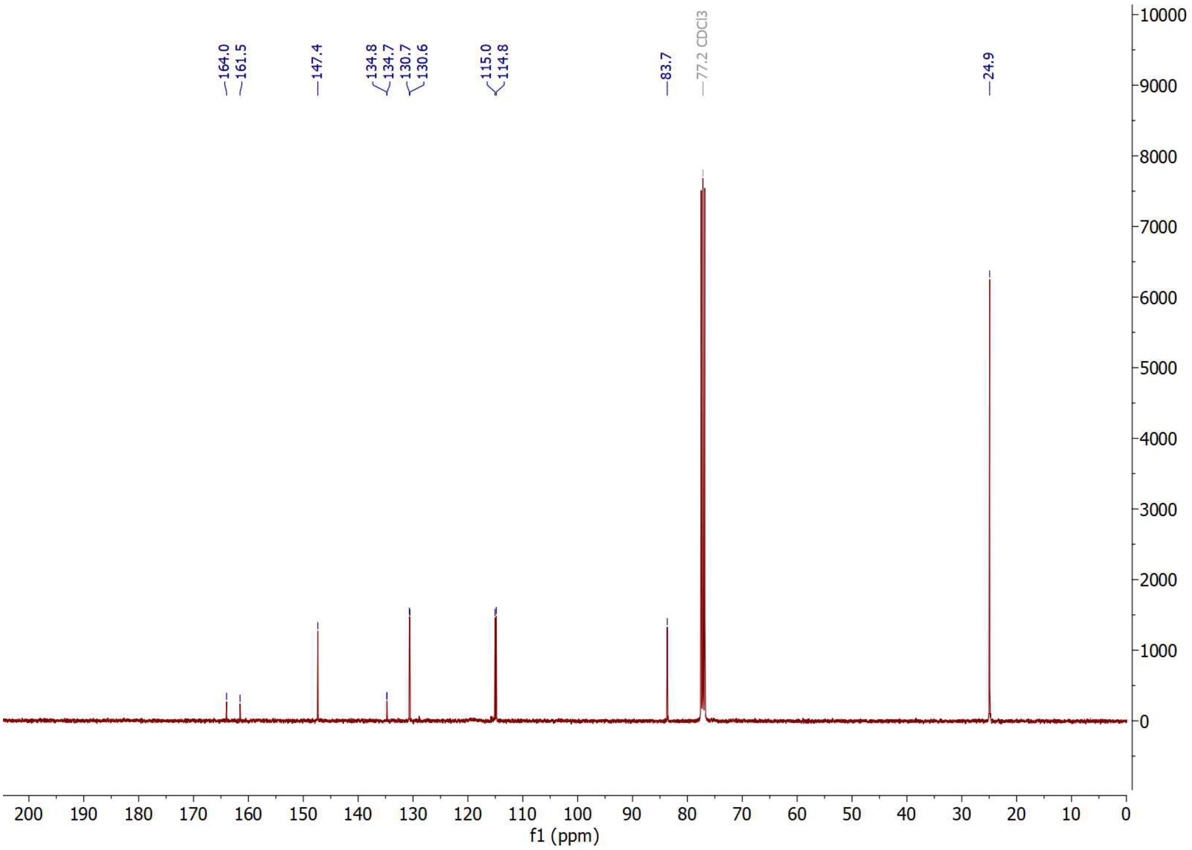

Figure S35. **^{13}C{^{1}H}-NMR spectrum of substrate 5e**

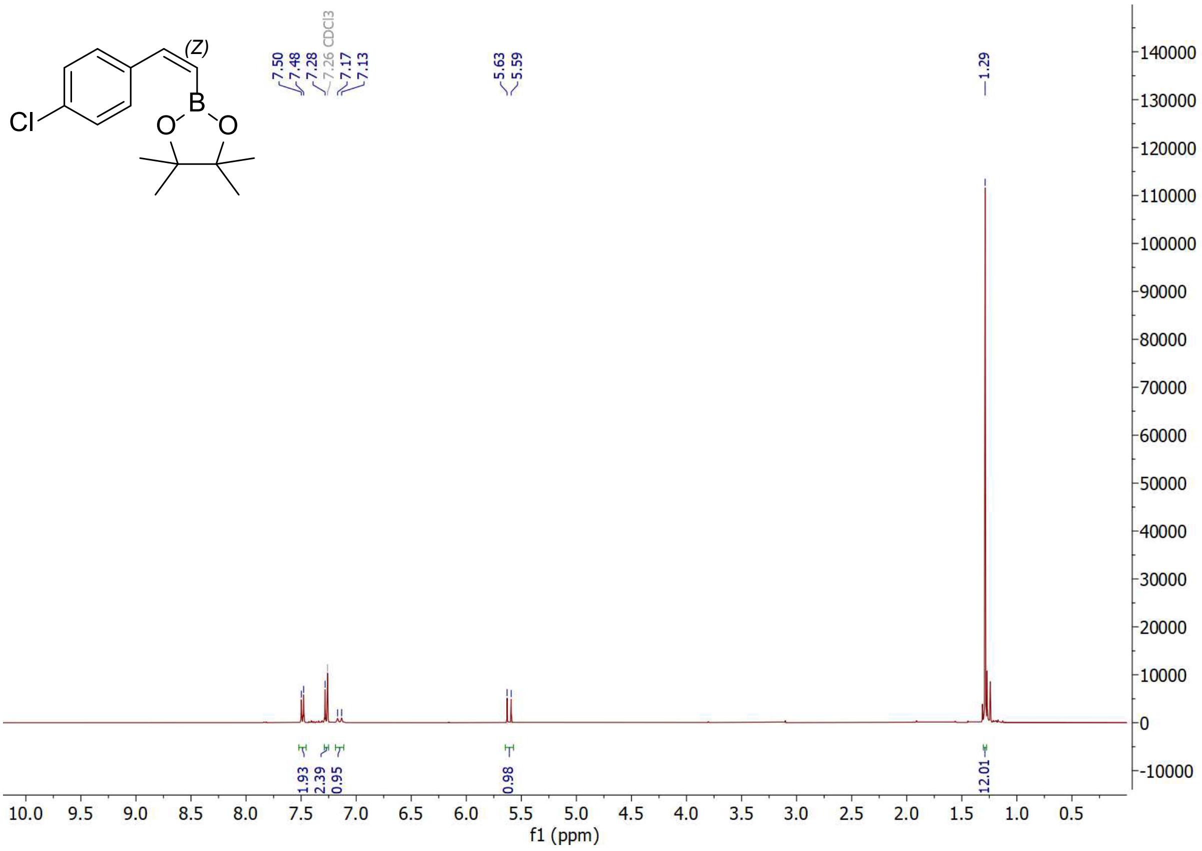

Figure S36. ^{1}H-NMR spectrum of substrate 5f

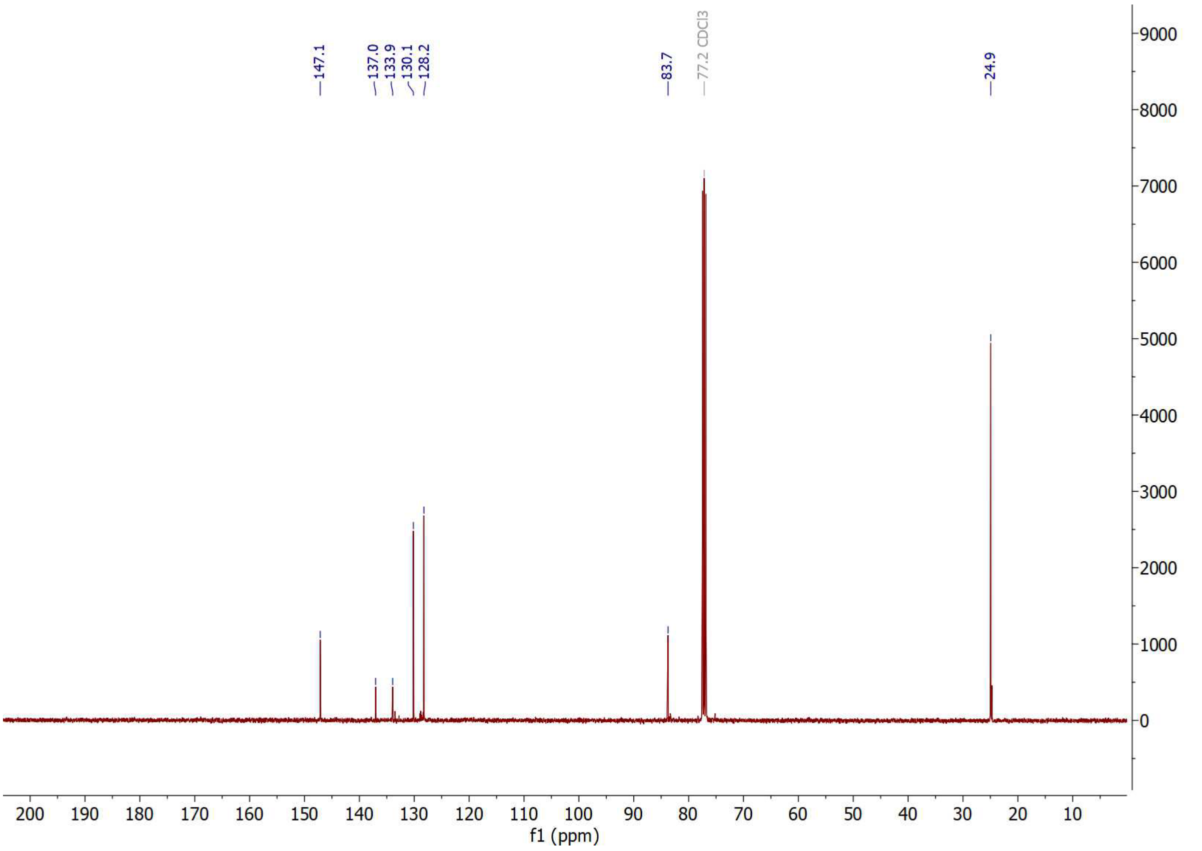

Figure S37. ^{13}C{^{1}H}-NMR spectrum of substrate 5f

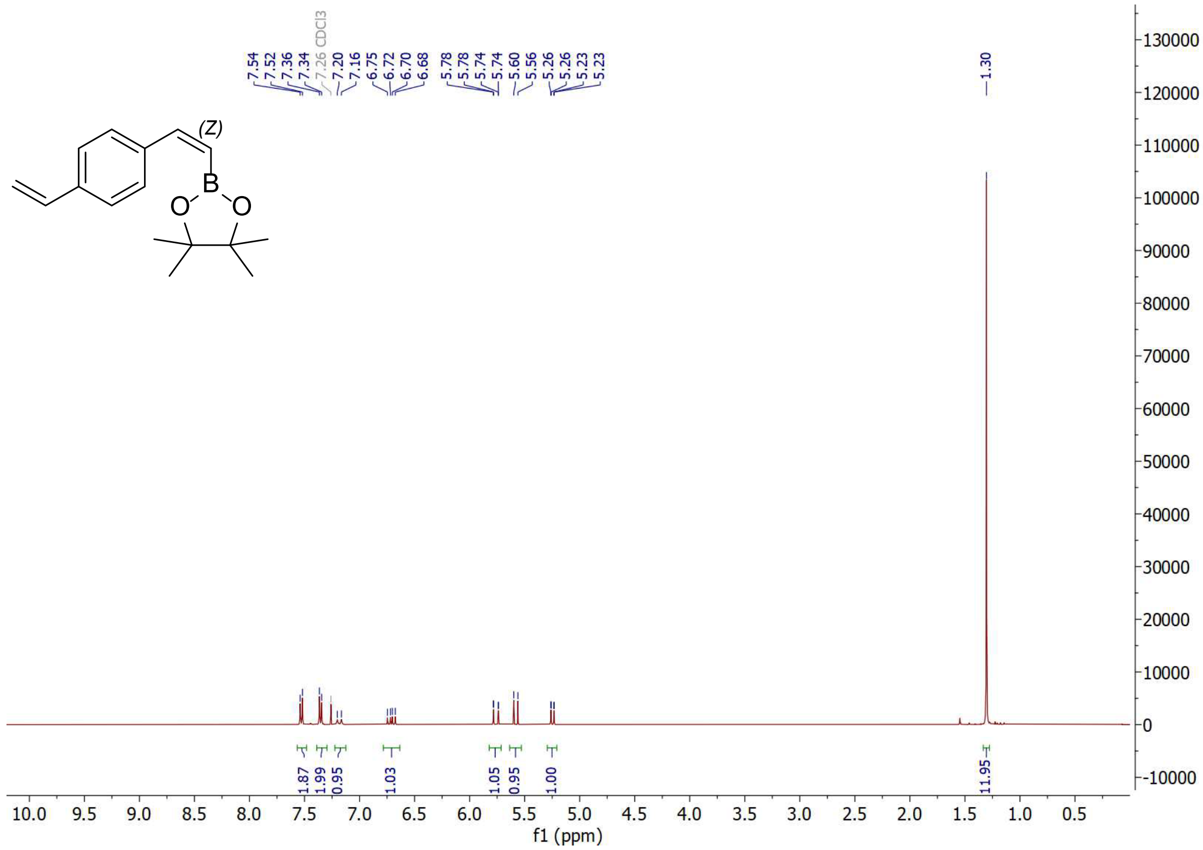

Figure S38. ^{1}H-NMR spectrum of substrate 5g

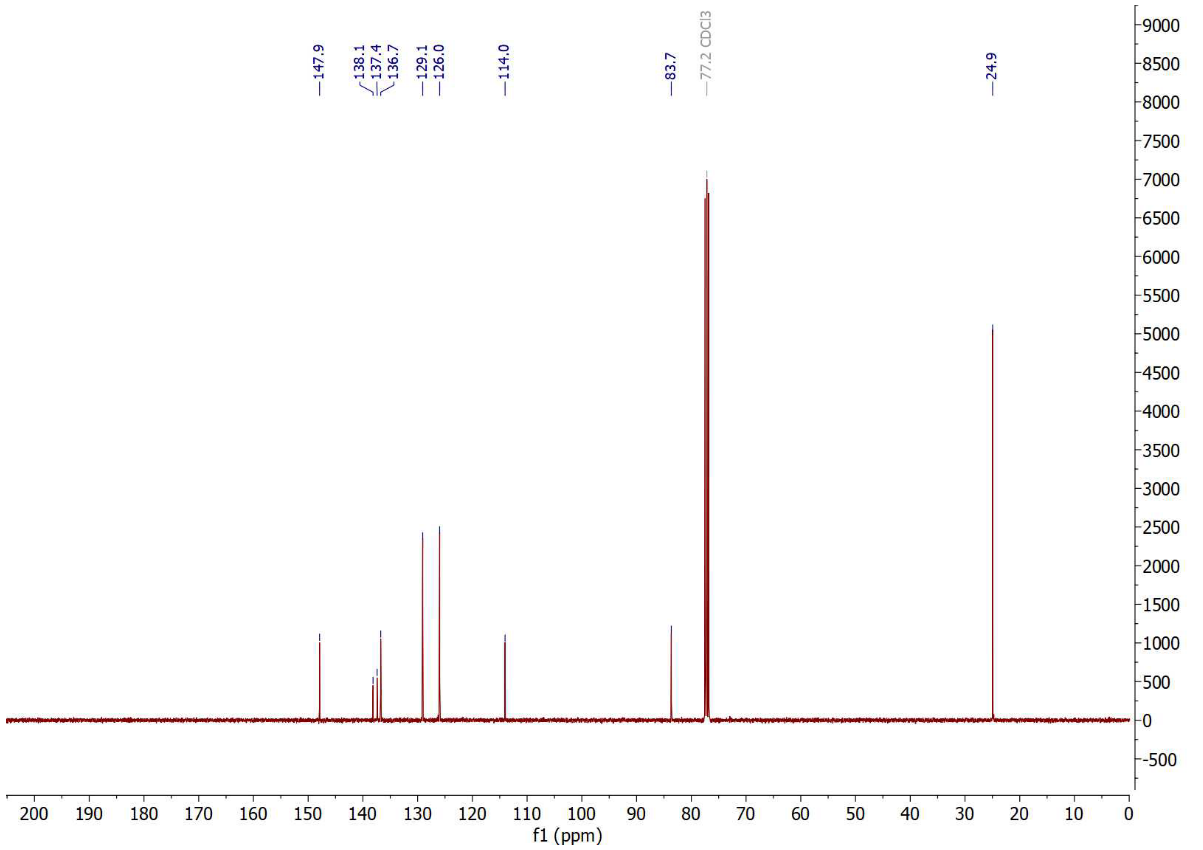

Figure S39. ^{13}C{^{1}H}-NMR spectrum of substrate 5g

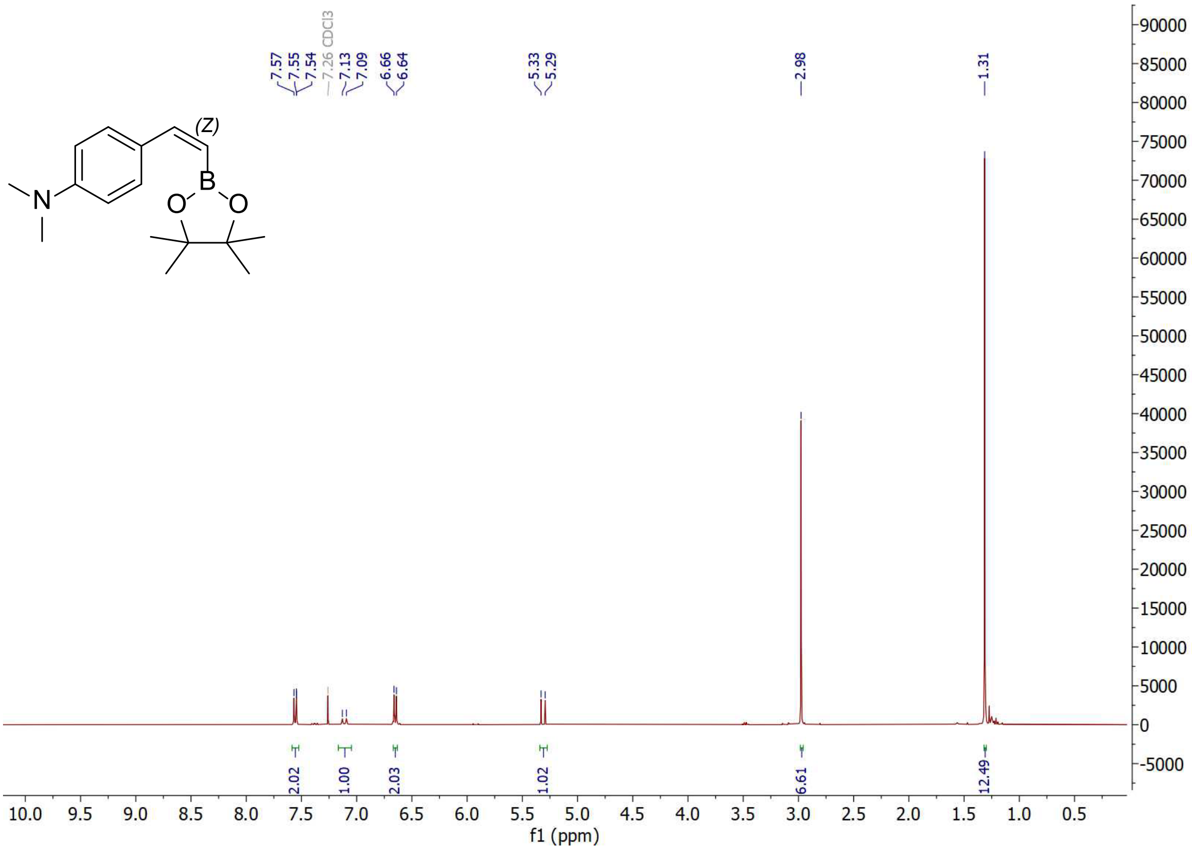

Figure S40. ^{1}**H-NMR spectrum of substrate 5h**

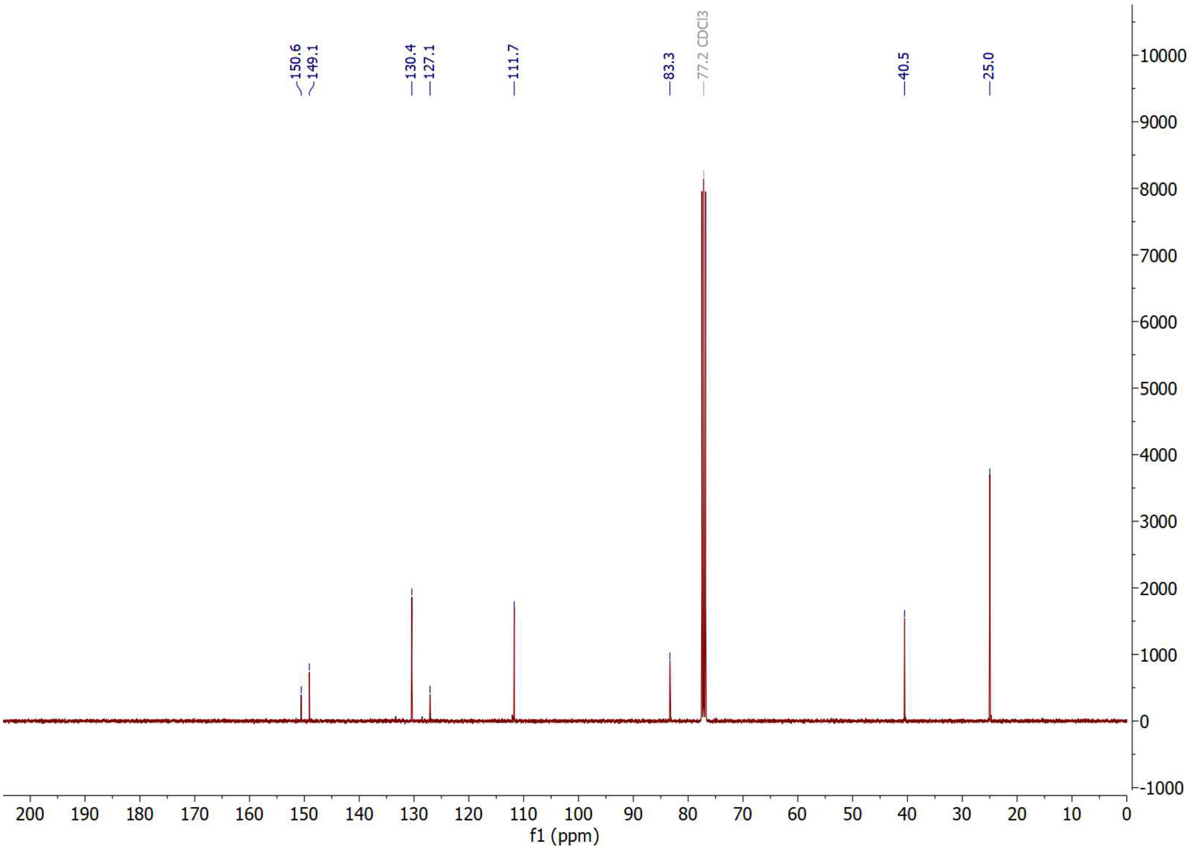

Figure S41. ^{13}**C{**^{1}**H}-NMR spectrum of substrate 5h**

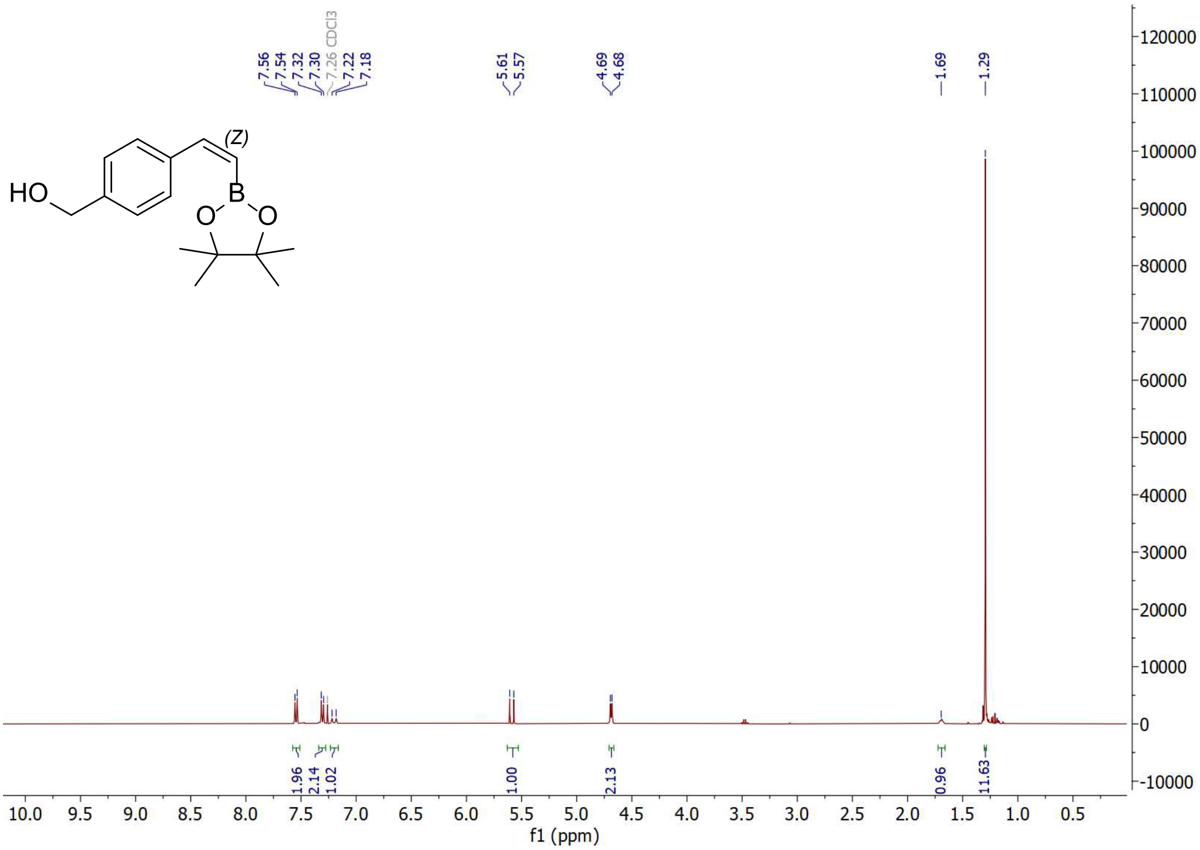

Figure S42. ^{1}H-NMR spectrum of substrate 5i

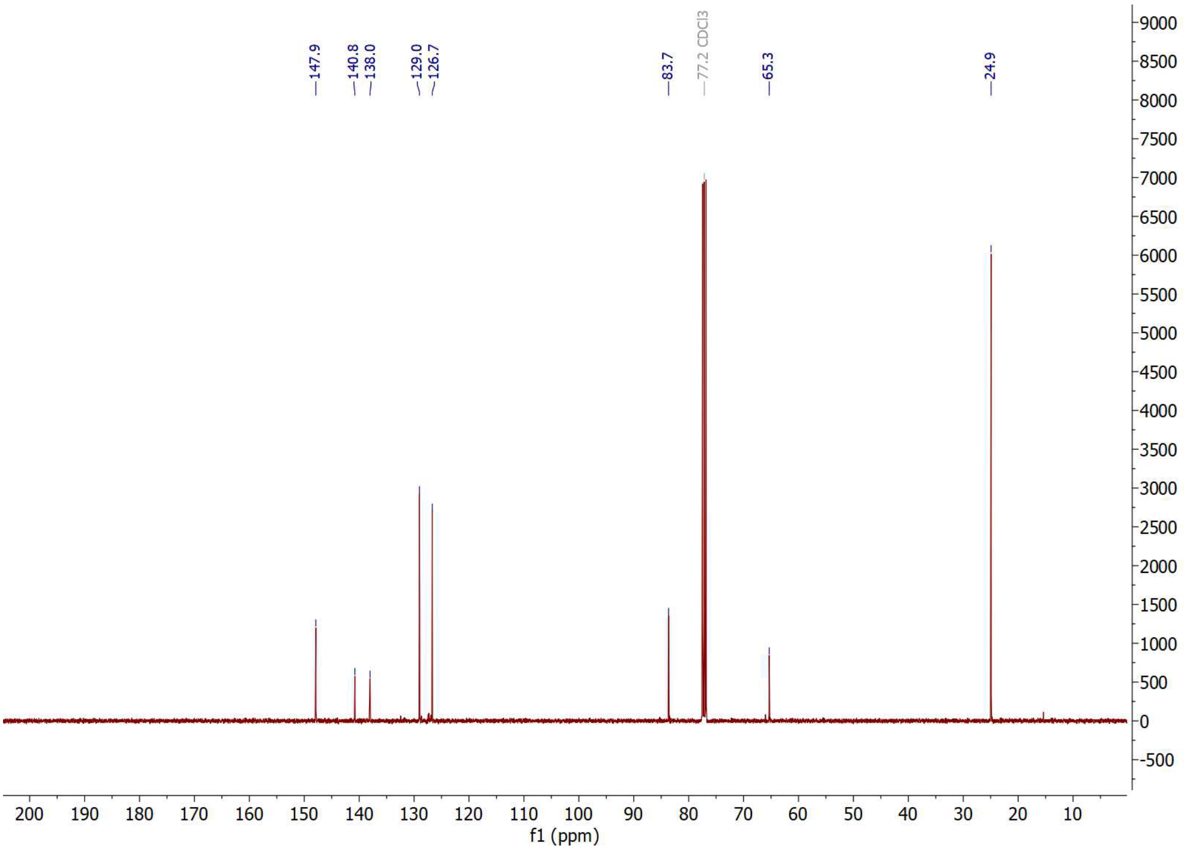

Figure S43. $^{13}C\{^{1}H\}$-NMR spectrum of substrate 5i

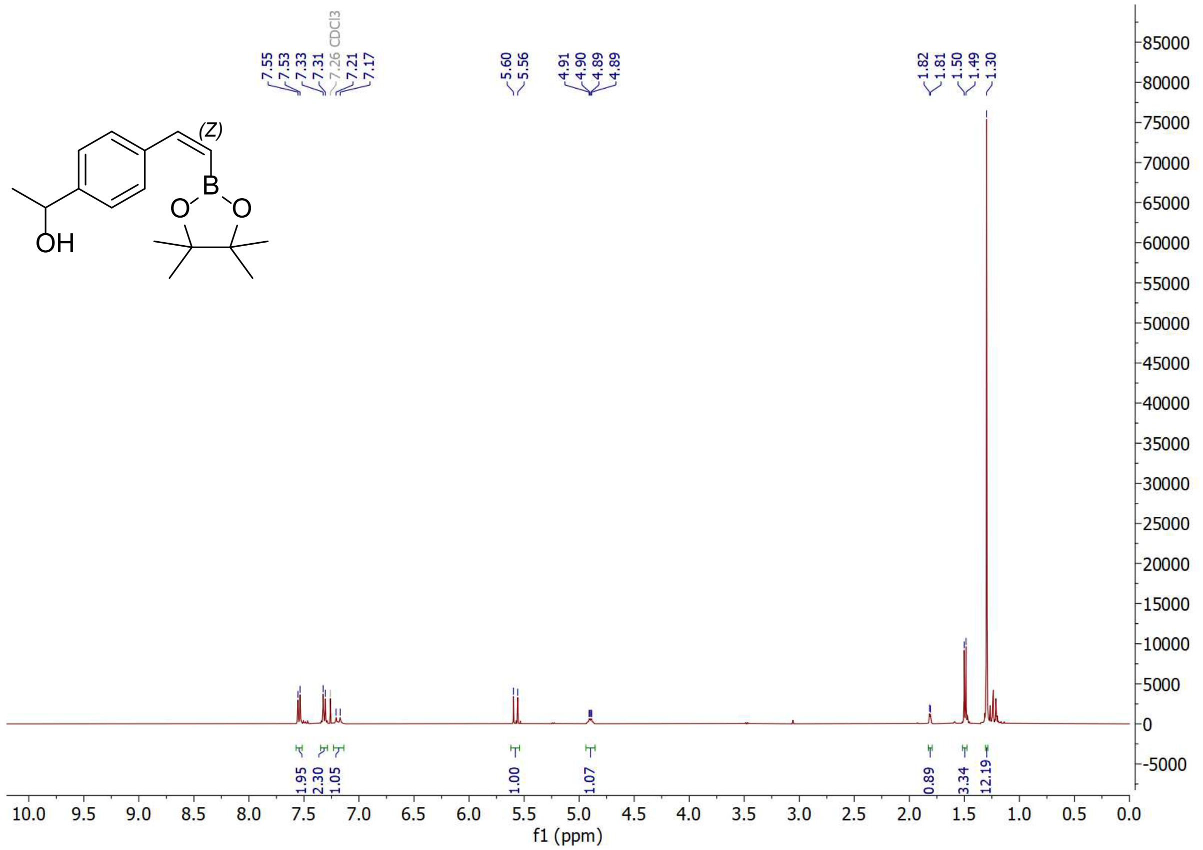

Figure S44. ^{1}H-NMR spectrum of substrate 5j

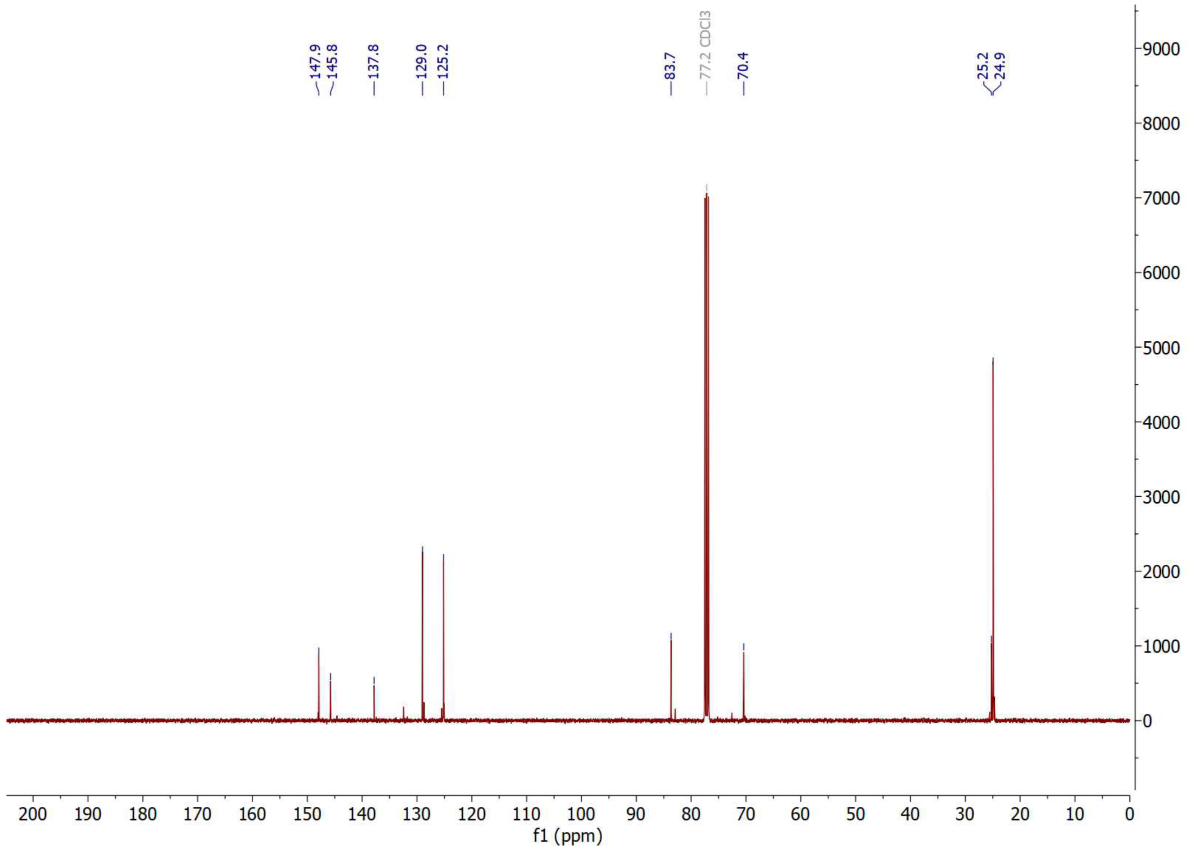

Figure S45. ^{13}C{^{1}H}-NMR spectrum of substrate 5j

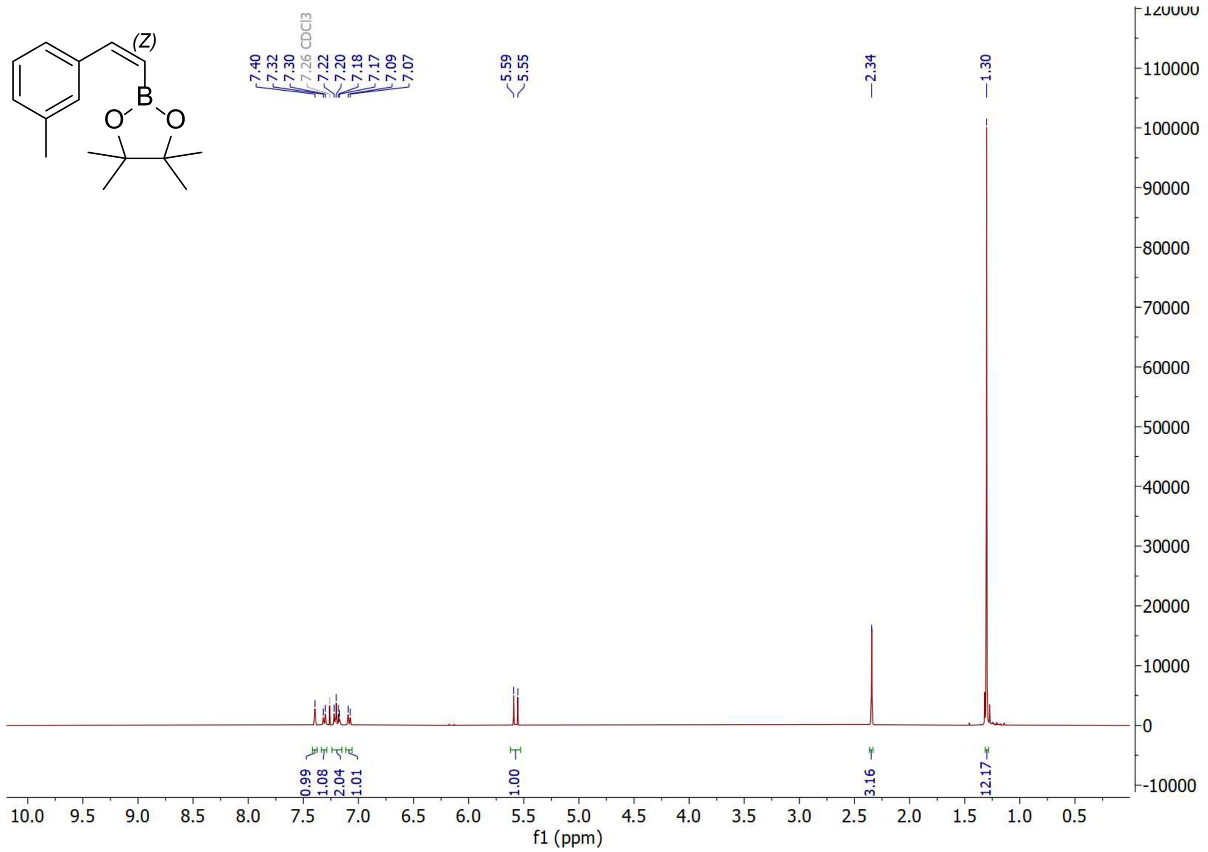

Figure S46. ^{1}H-NMR spectrum of substrate 5k

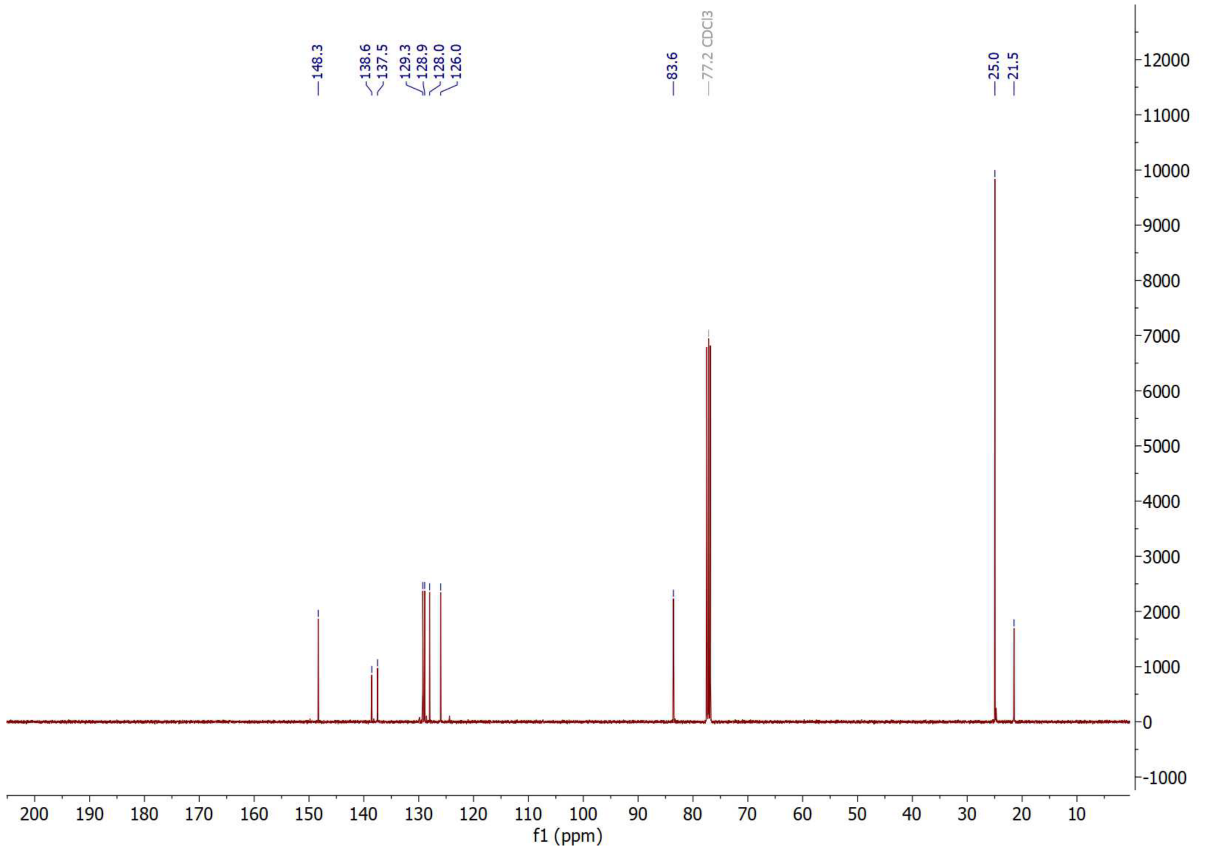

Figure S47. $^{13}C\{^{1}H\}$-NMR spectrum of substrate 5k

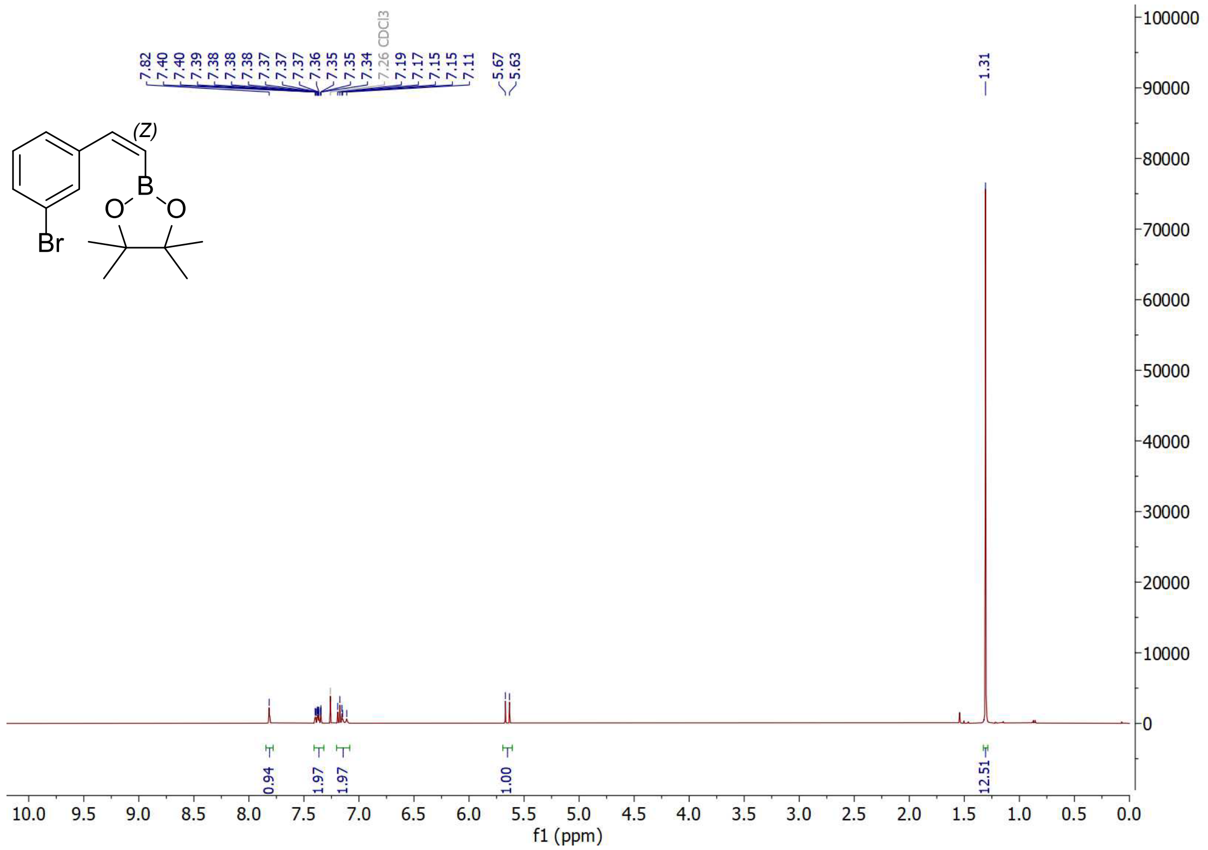

Figure S48. ^{1}H-NMR spectrum of substrate 5l

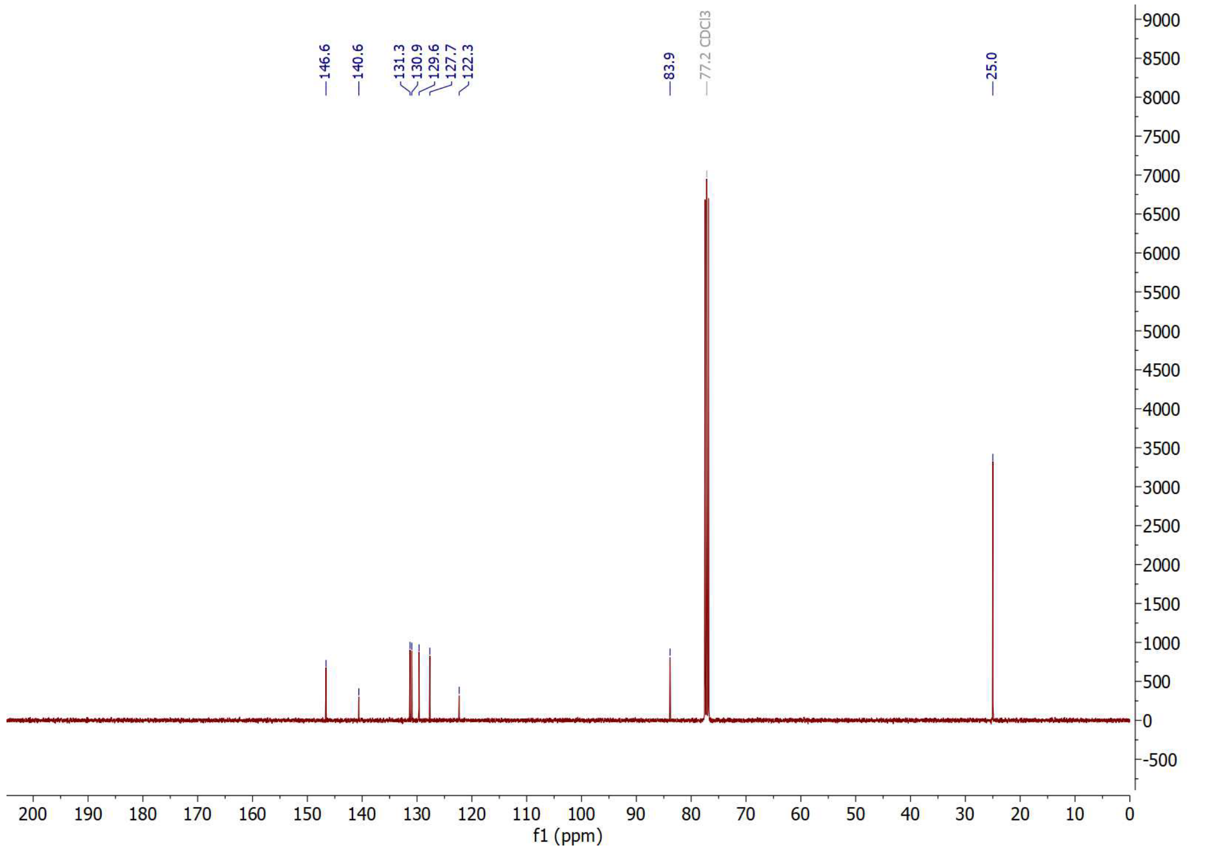

Figure S49. ^{13}C{^{1}H}-NMR spectrum of substrate 5l

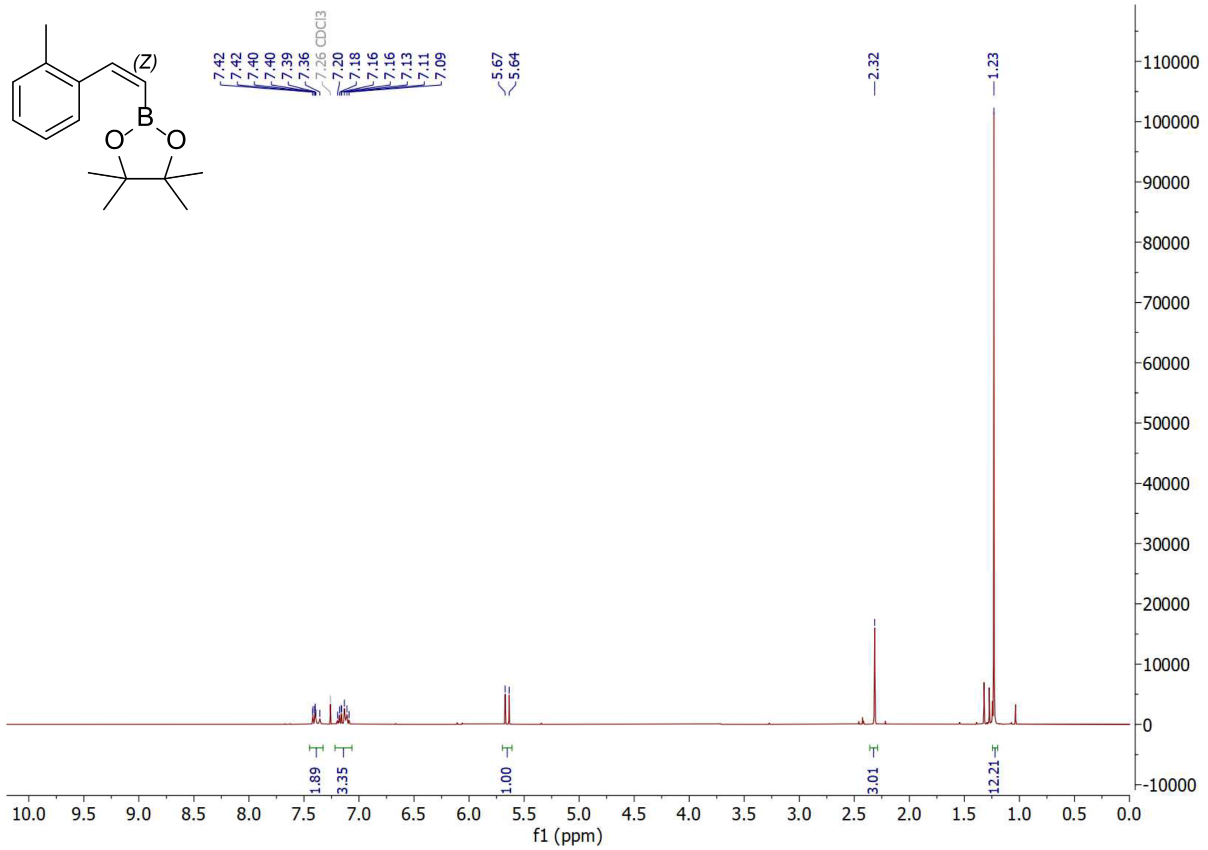

Figure S50. ^{1}H-NMR spectrum of substrate 5m

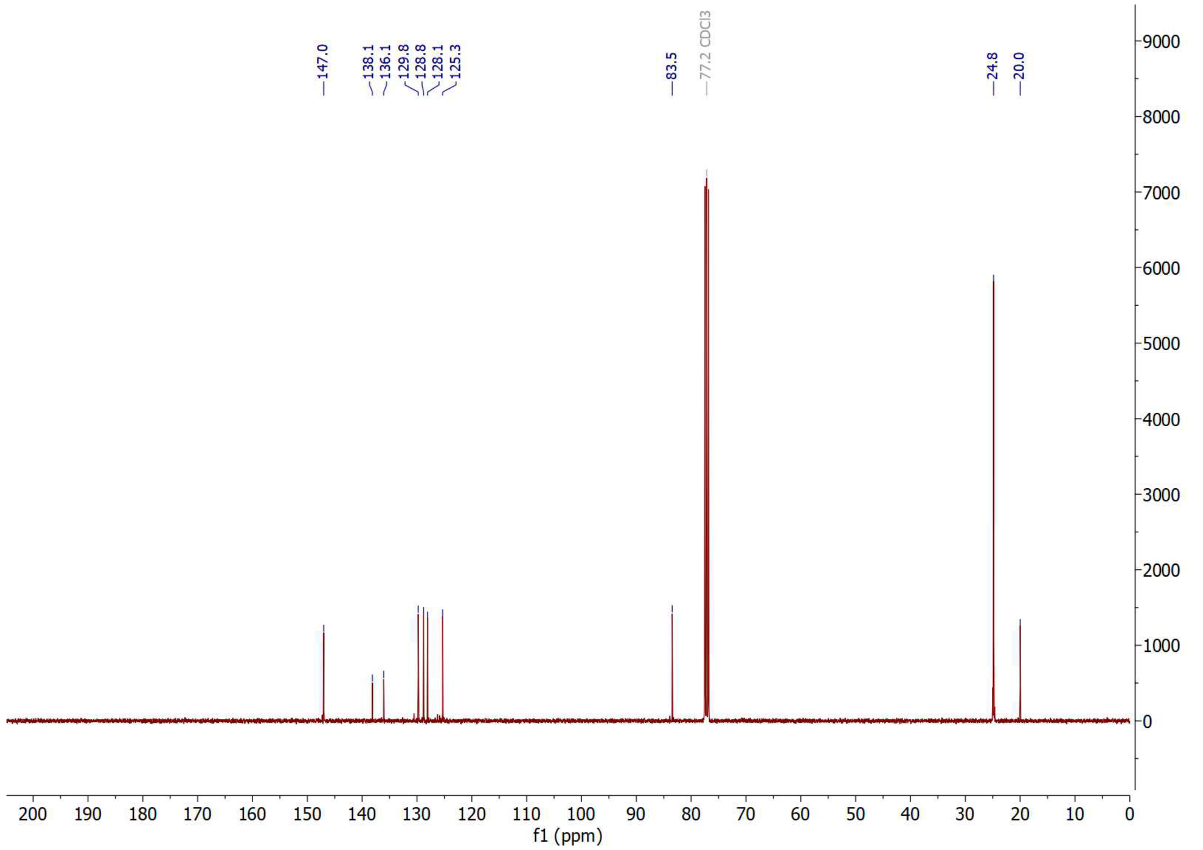

Figure S51. ^{13}C{^{1}H}-NMR spectrum of substrate 5m

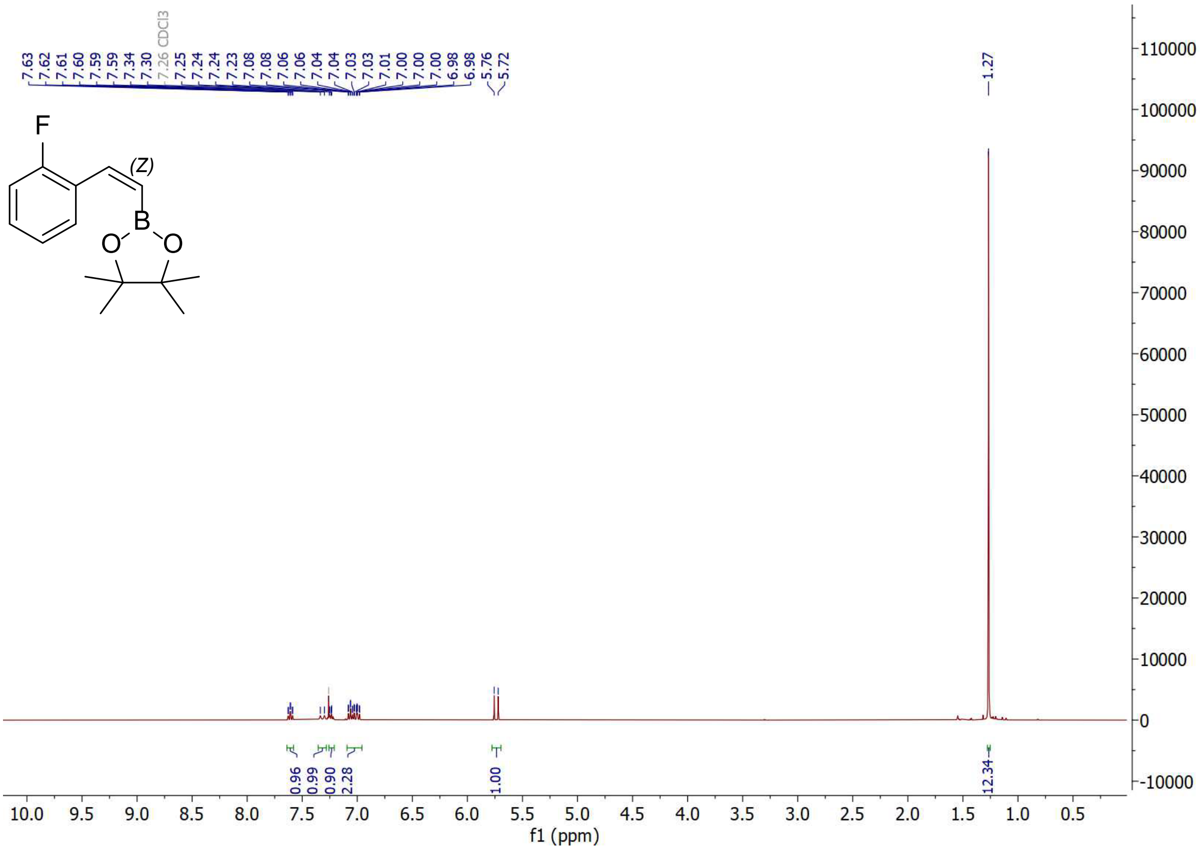

Figure S52. ^{1}H-NMR spectrum of substrate 5n

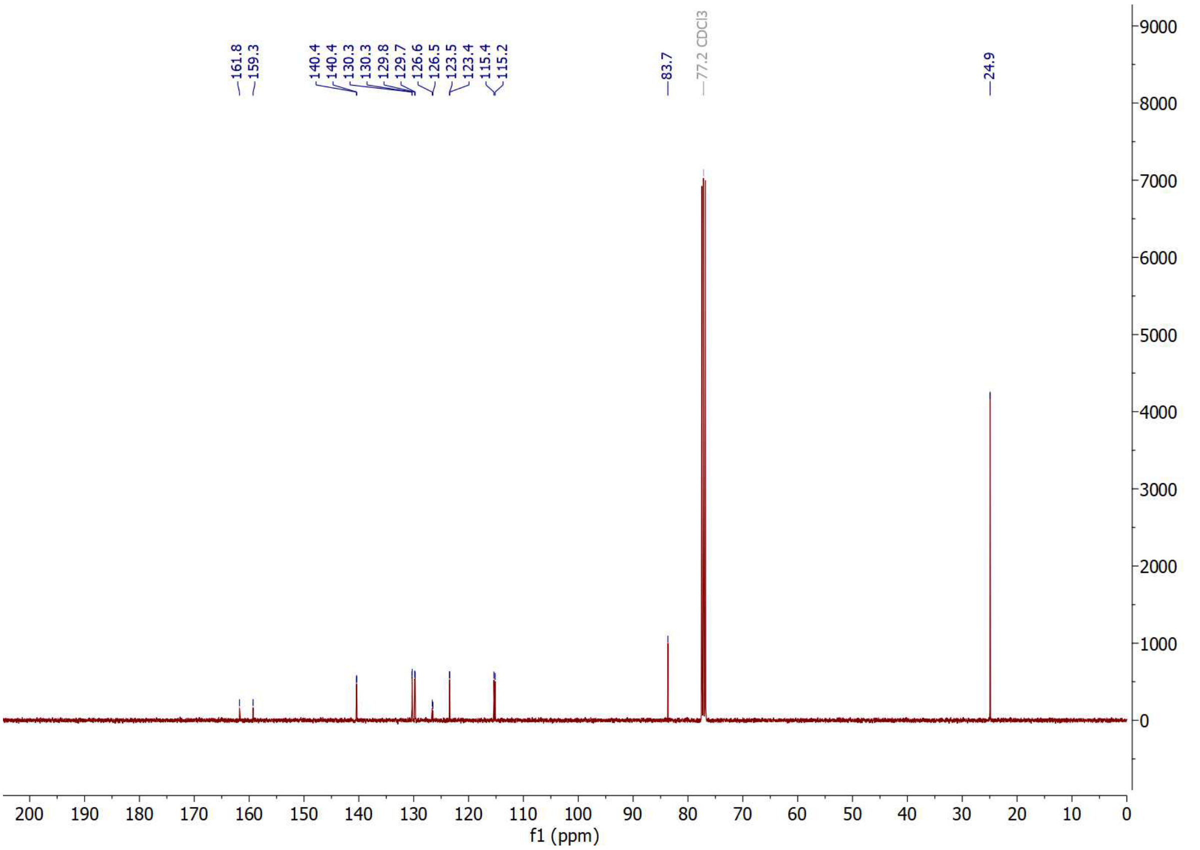

Figure S53. ^{13}C{^{1}H}-NMR spectrum of substrate 5n

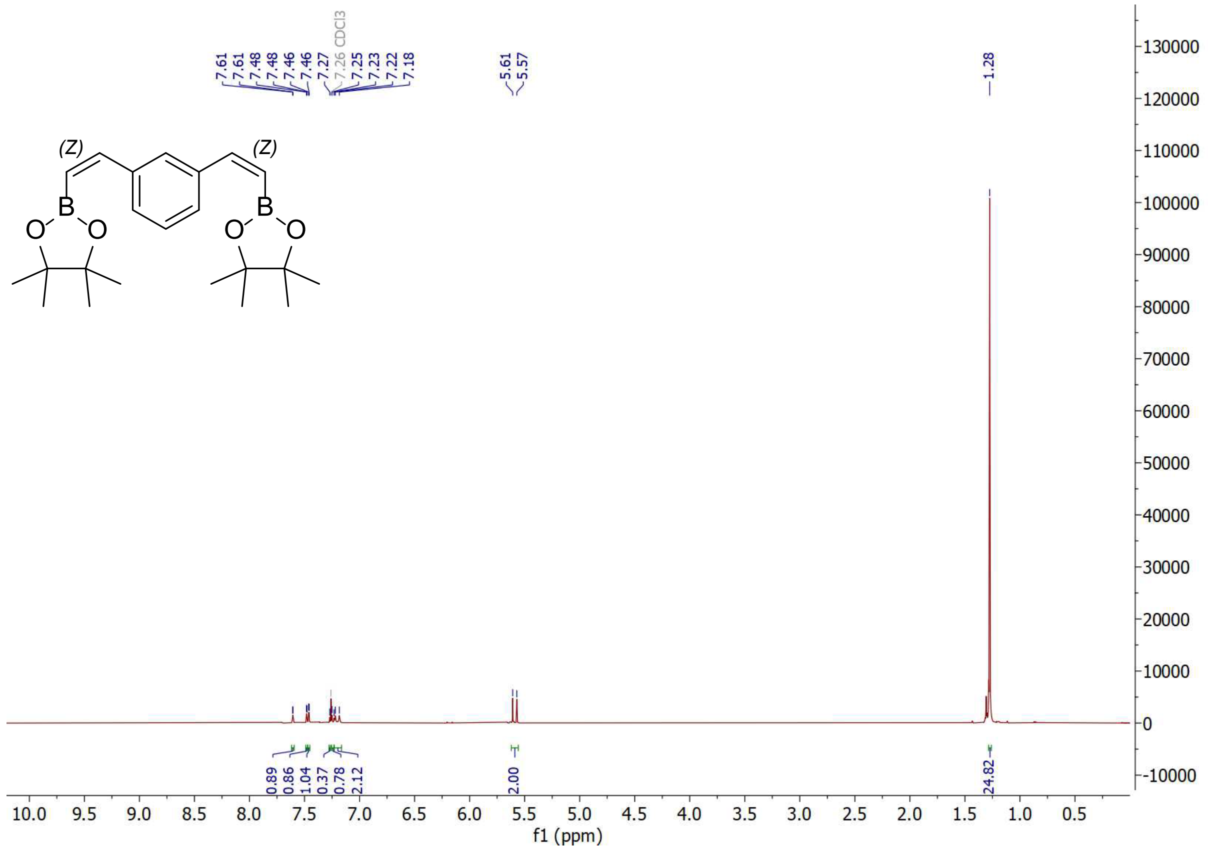

Figure S54. ^{1}H-NMR spectrum of substrate 5o

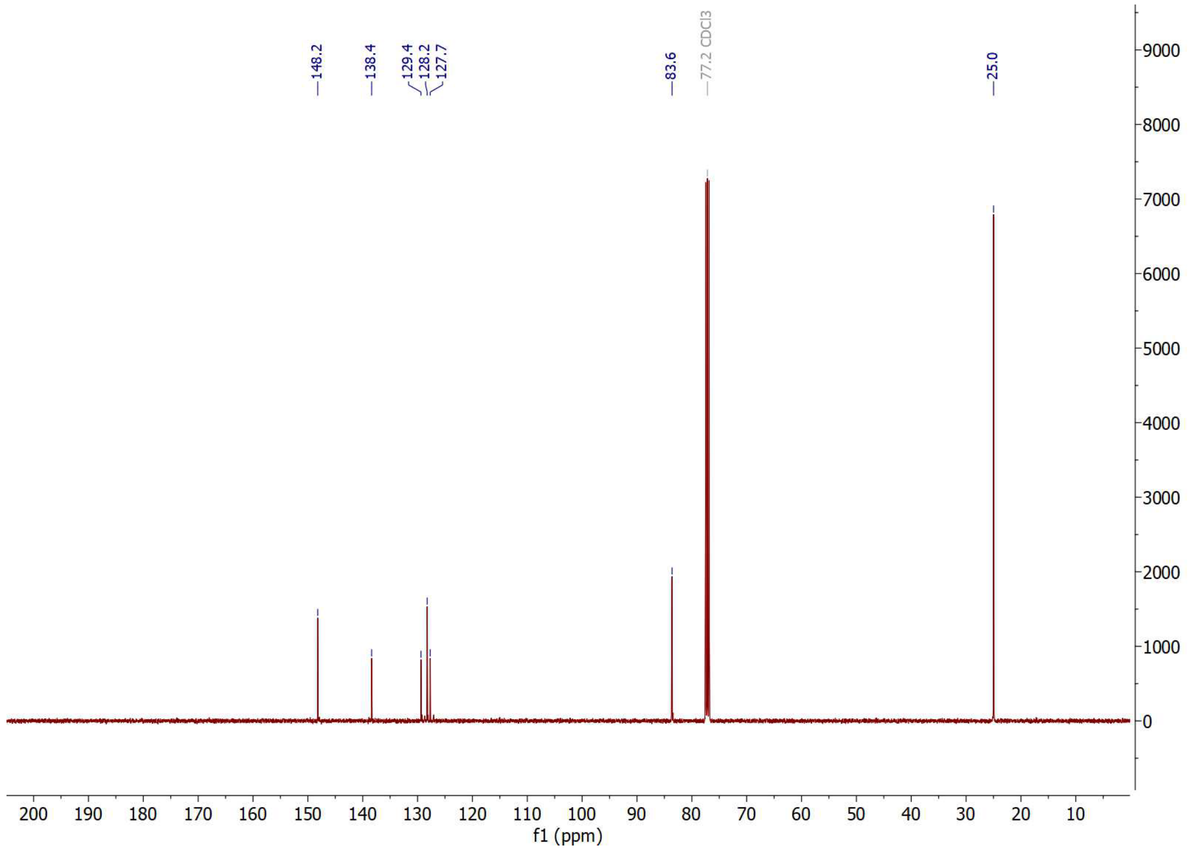

Figure S55. ^{13}C{^{1}H}-NMR spectrum of substrate 5o

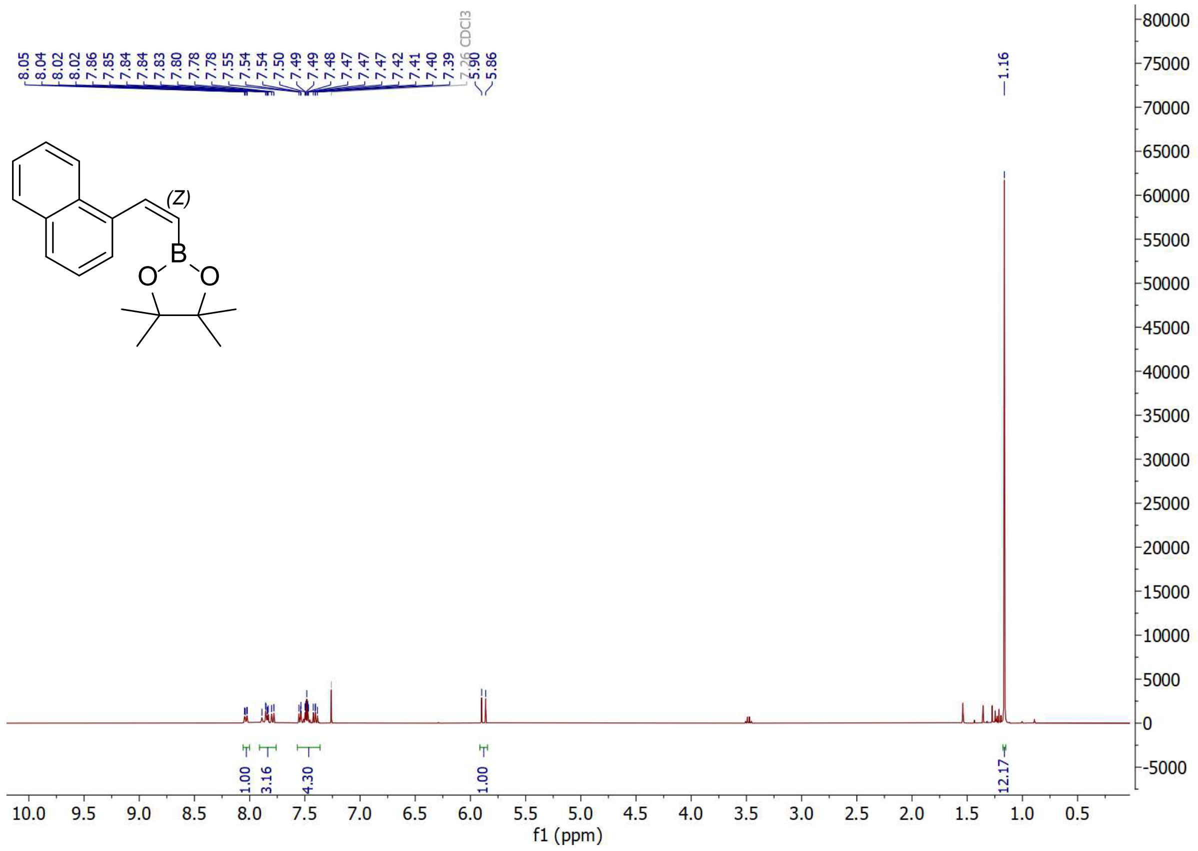

Figure S56. **^{1}H-NMR spectrum of substrate 5p**

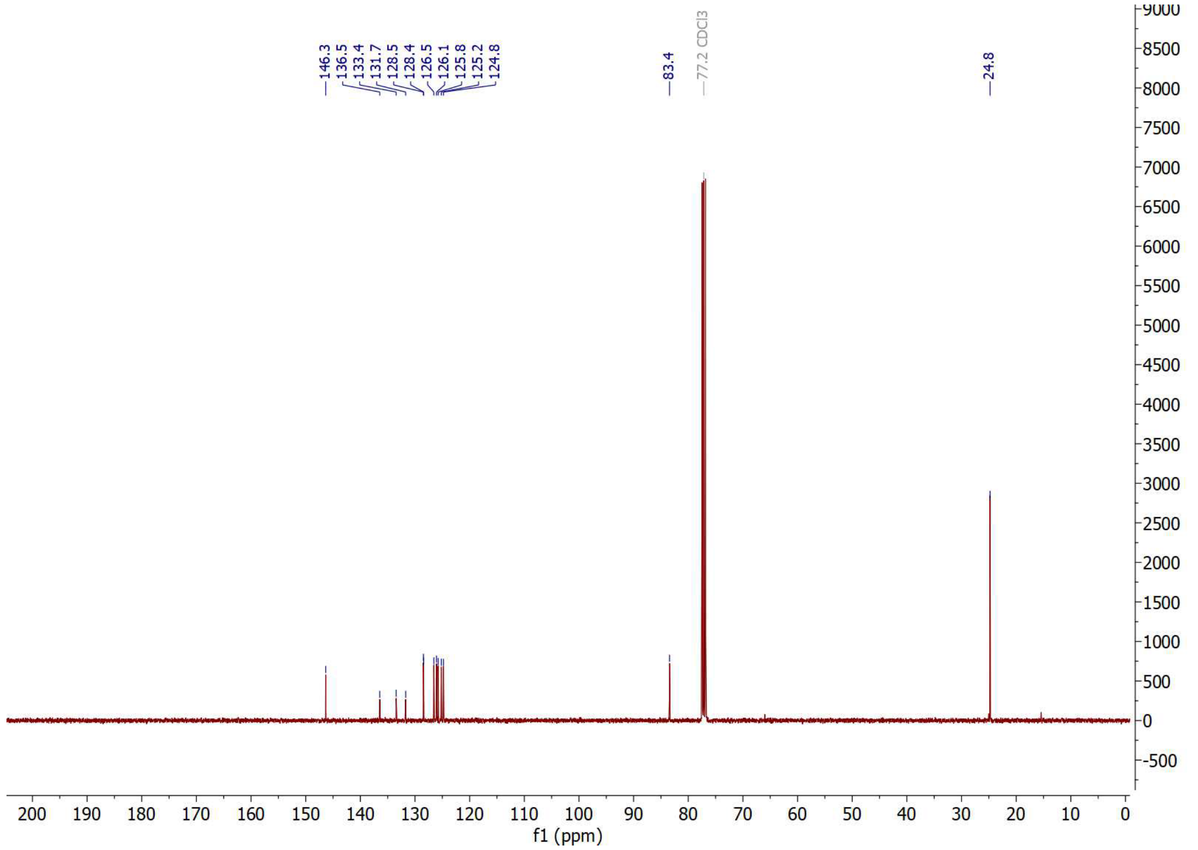

Figure S57. **^{13}C{^{1}H}-NMR spectrum of substrate 5p**

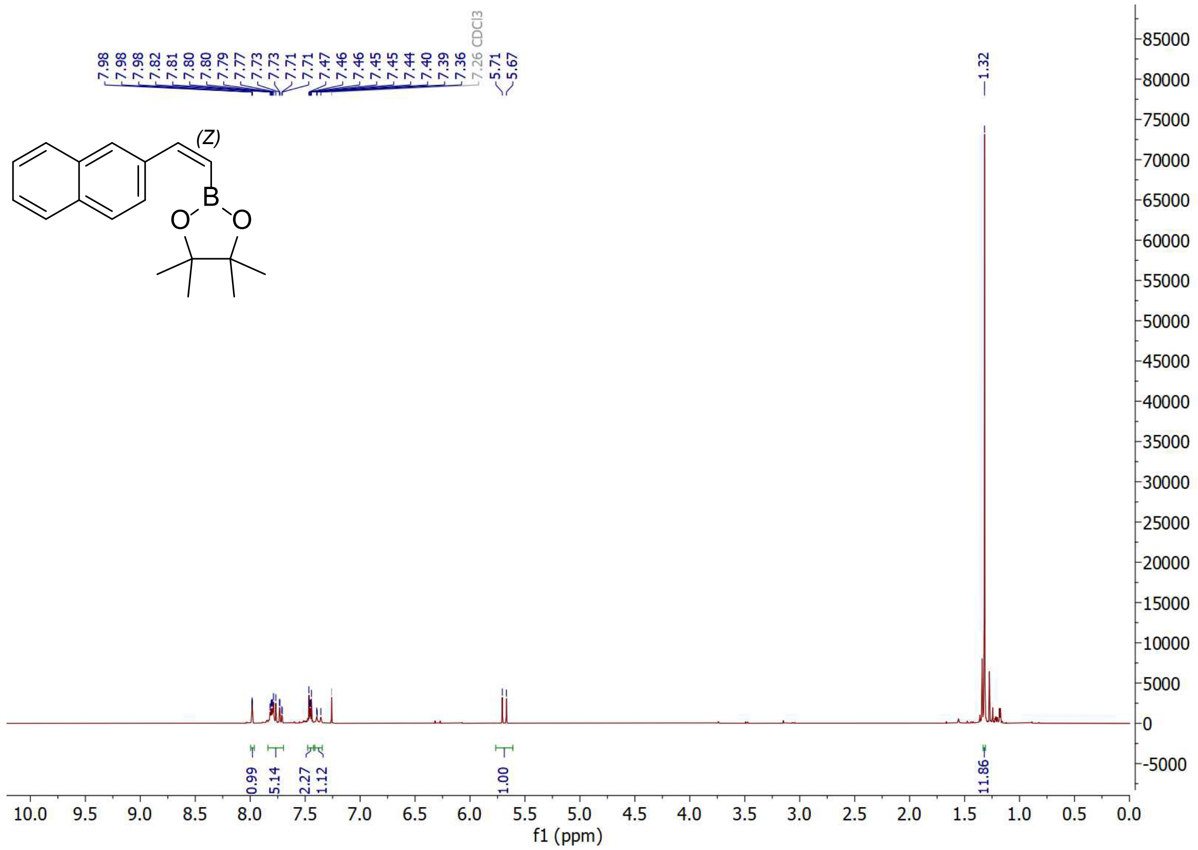

Figure S58. **^{1}H-NMR spectrum of substrate 5q**

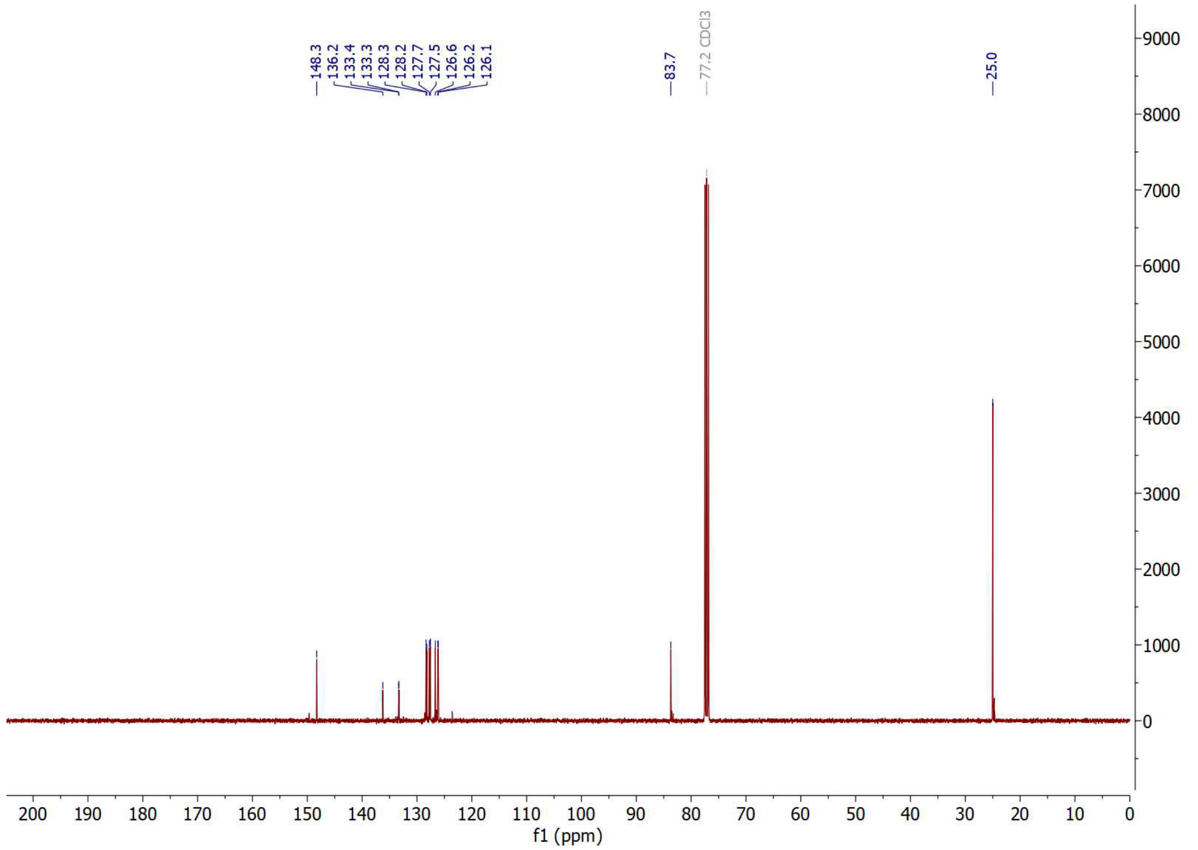

Figure S59. **^{13}C{^{1}H}-NMR spectrum of substrate 5q**

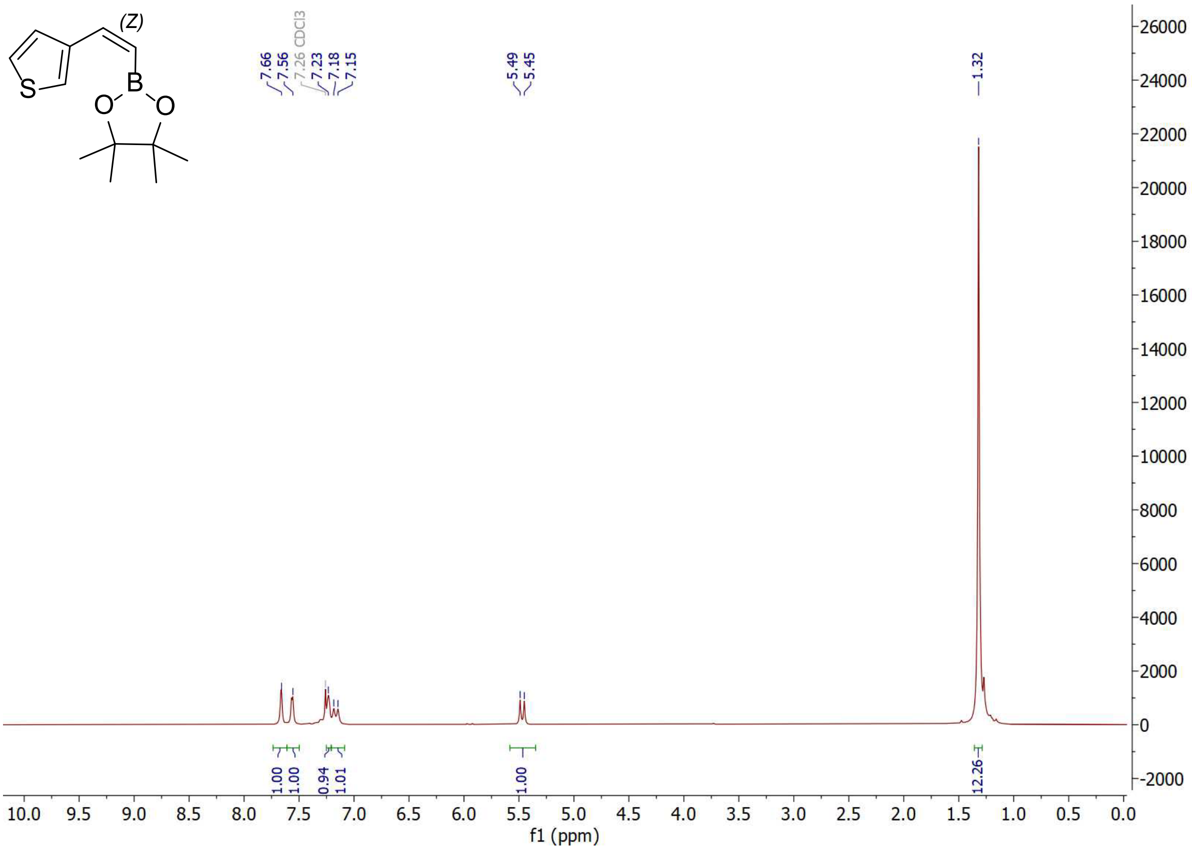

Figure S60. ^{1}**H-NMR spectrum of substrate 5r**

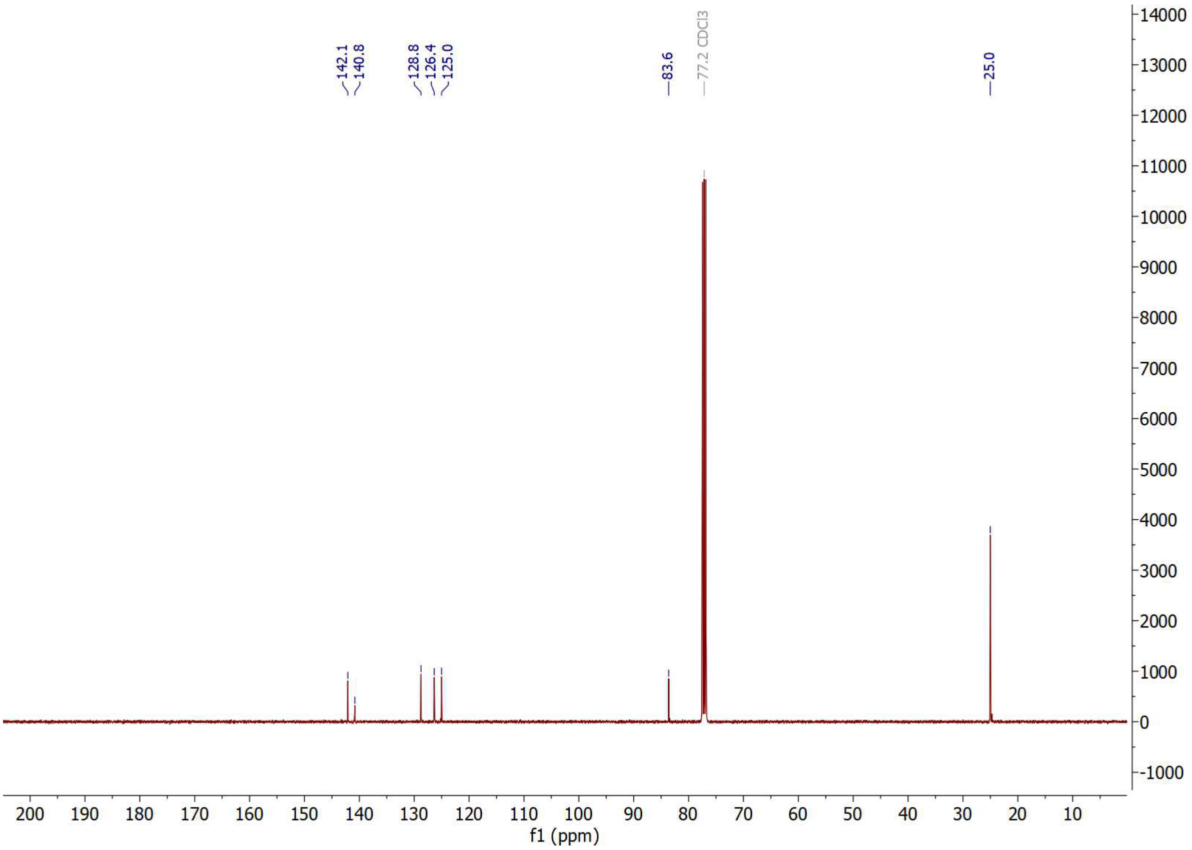

Figure S61. ^{13}**C{**^{1}**H}-NMR spectrum of substrate 5r**

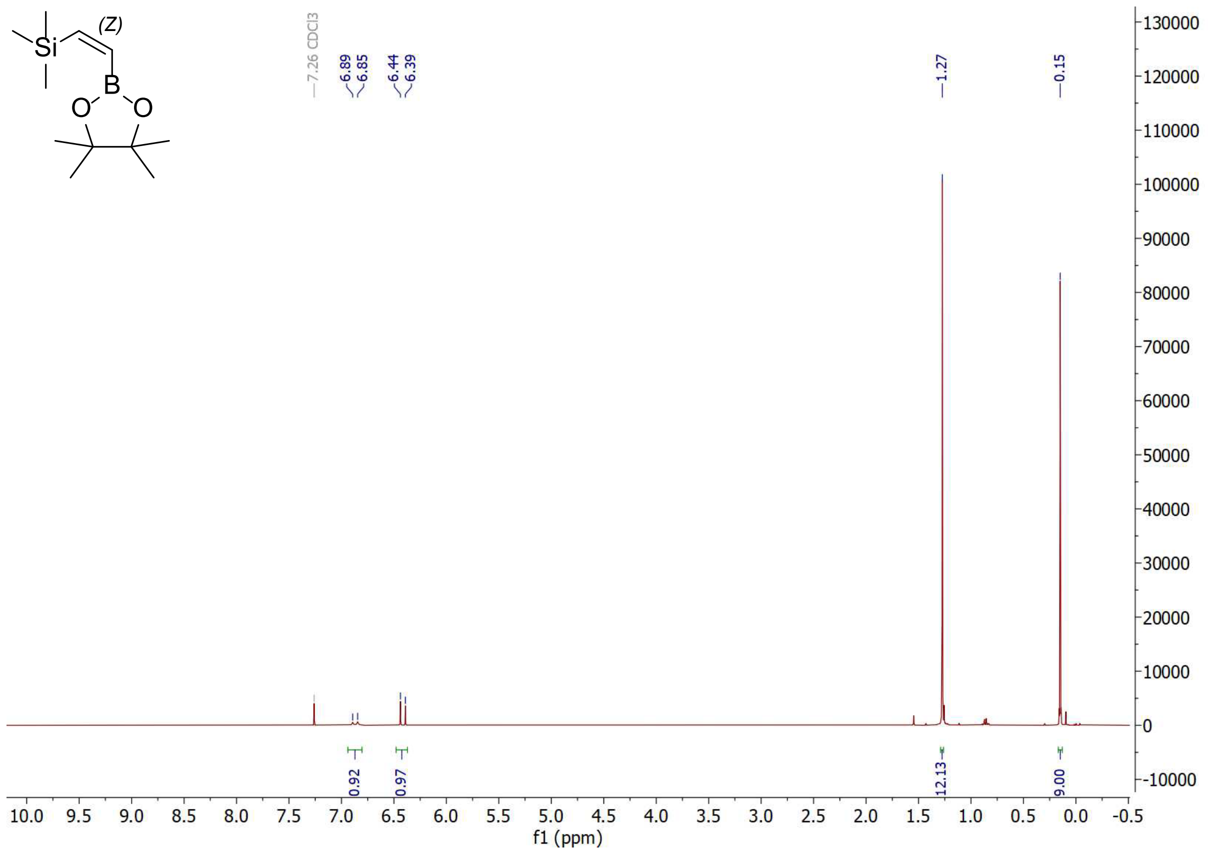

Figure S62. **^{1}H-NMR spectrum of substrate 5s**

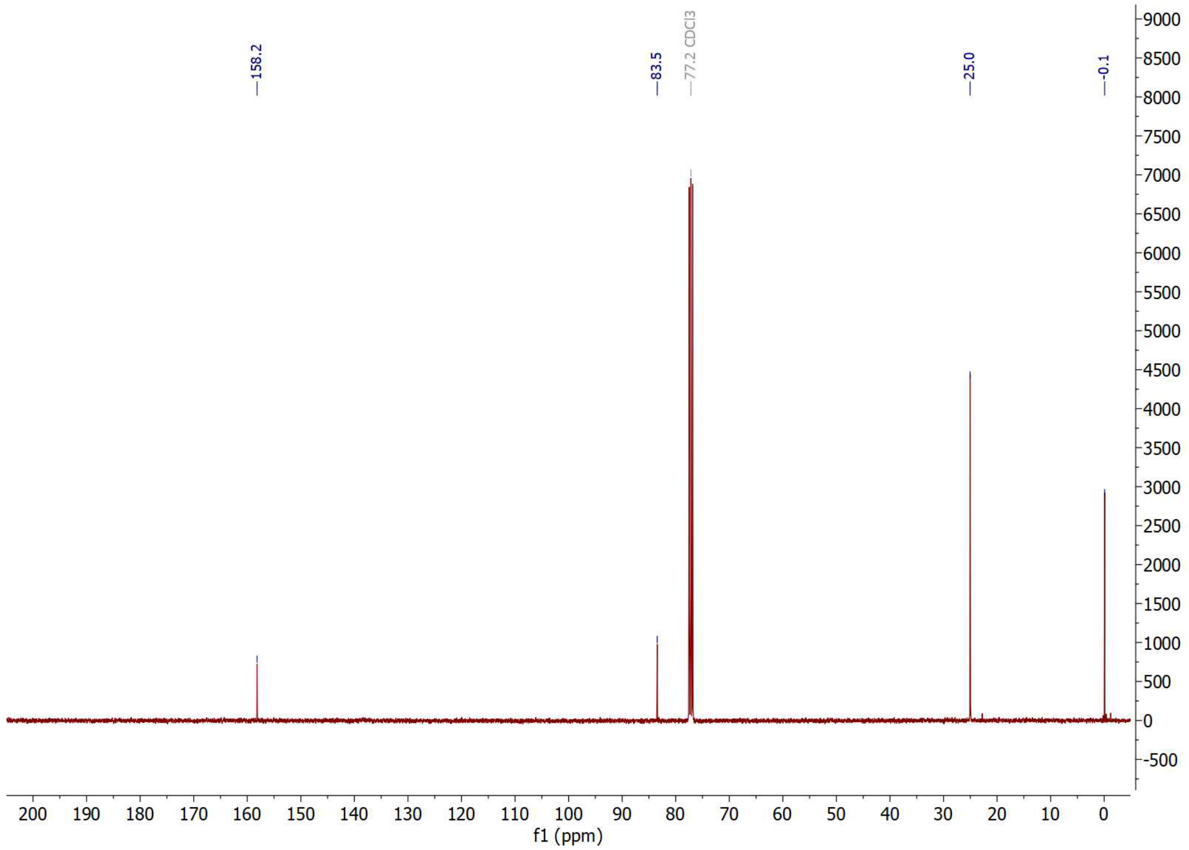

Figure S63. **^{13}C{^{1}H}-NMR spectrum of substrate 5s**

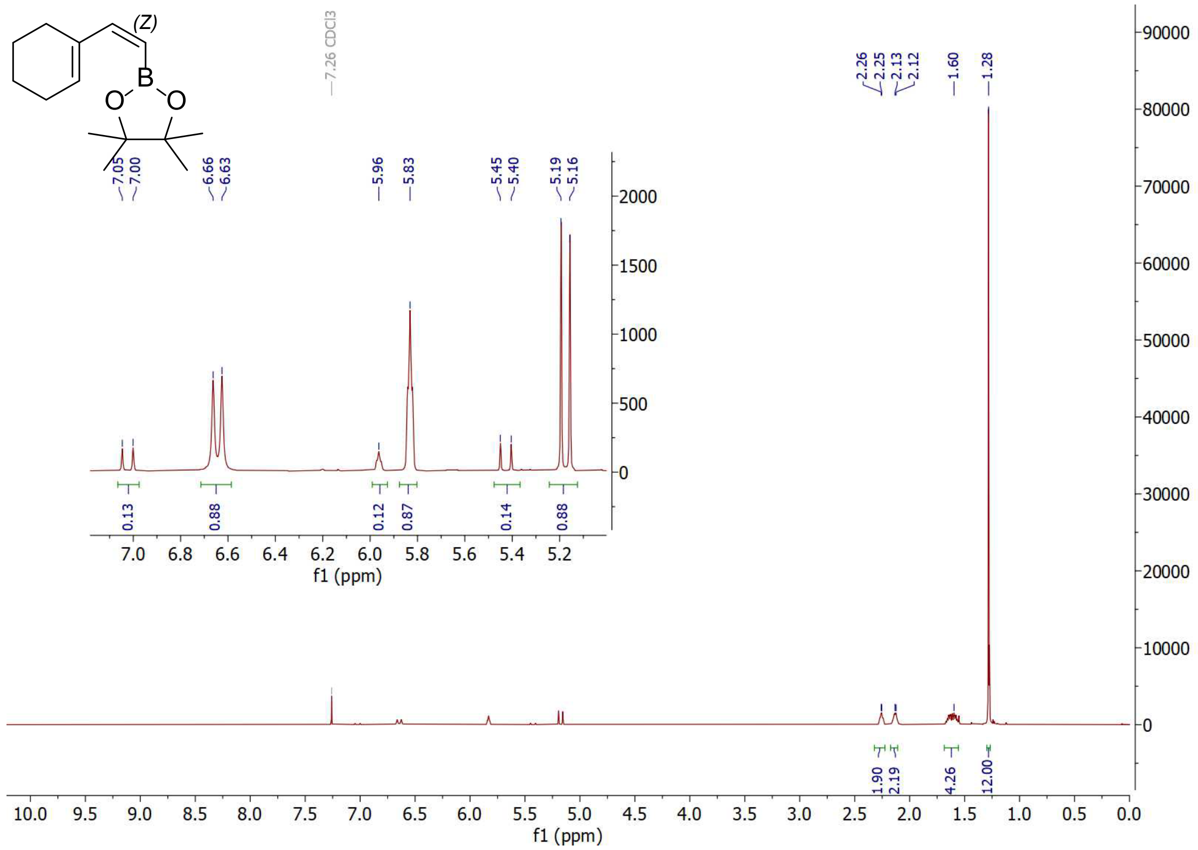

Figure S64. ^{1}H-NMR spectrum of substrate 5t

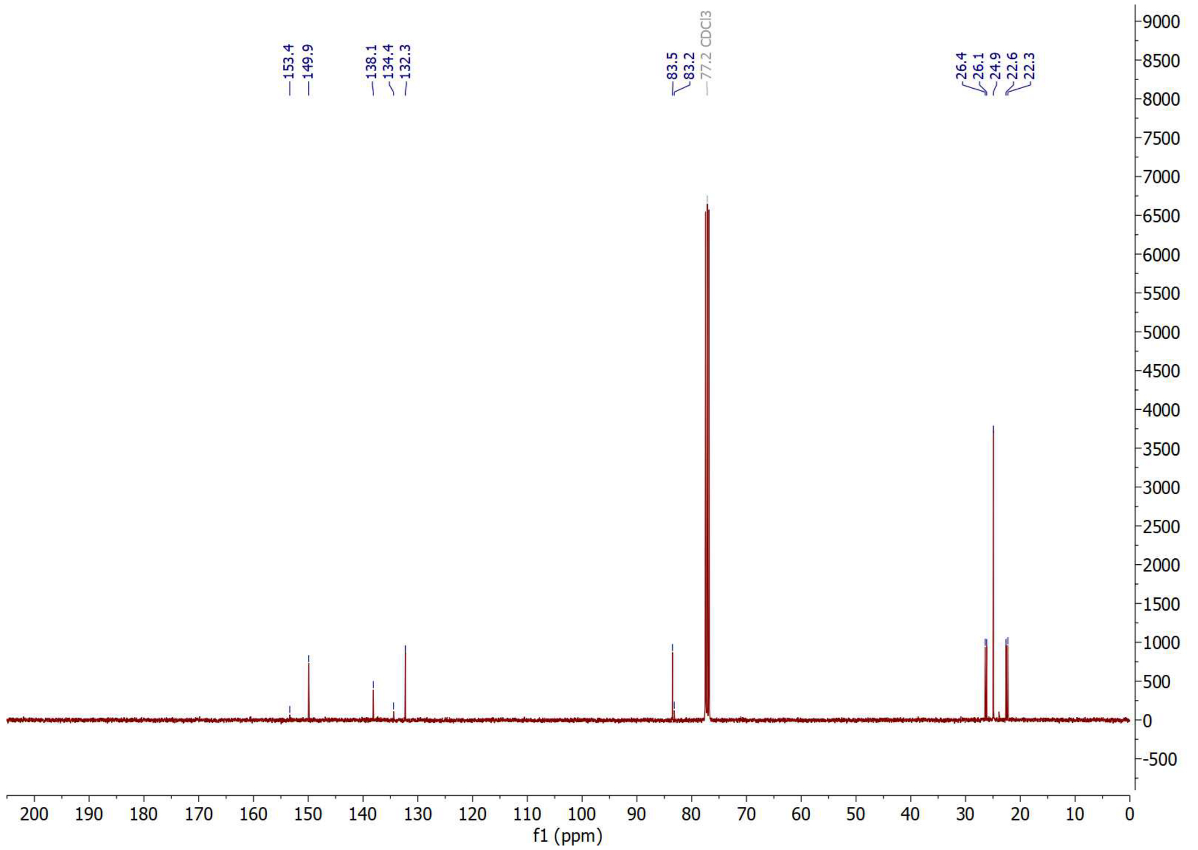

Figure S65. ^{13}C{^{1}H}-NMR spectrum of substrate 5t

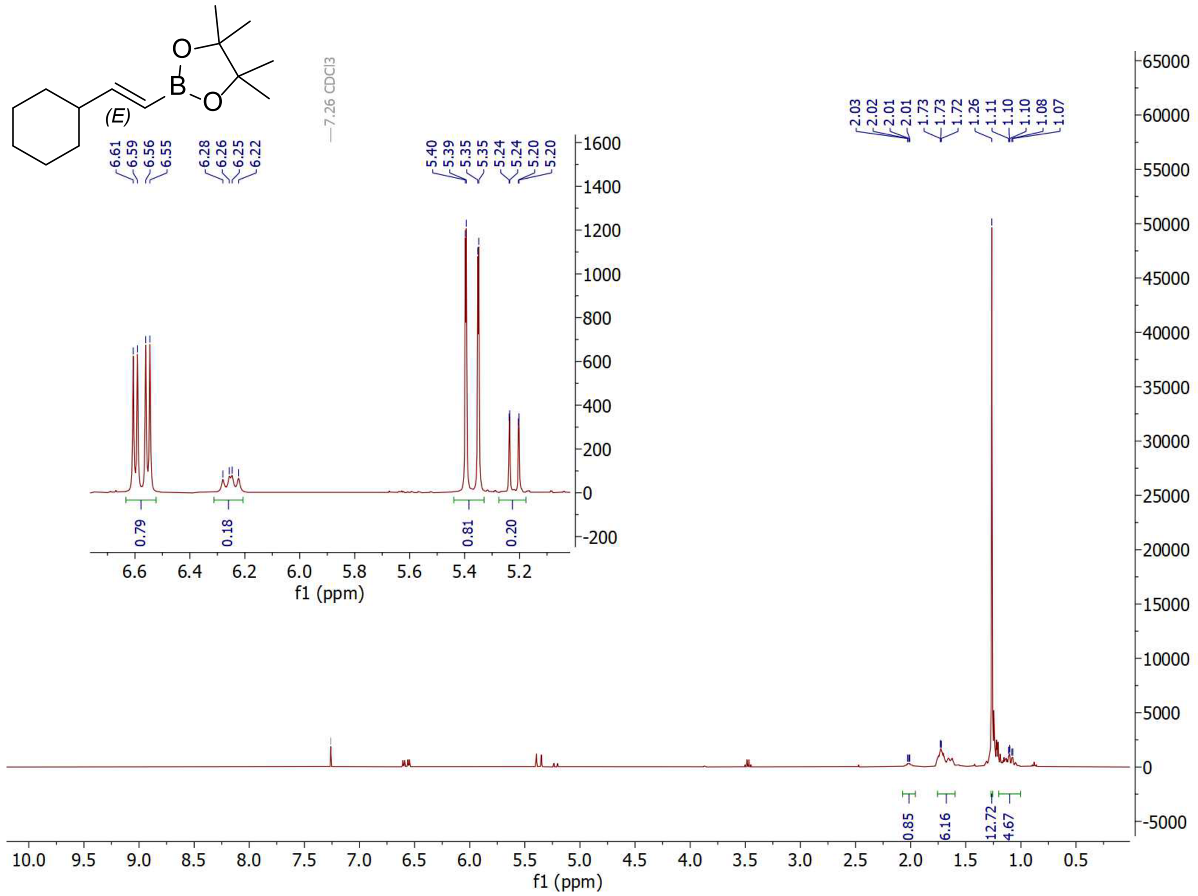

Figure S66. ^{1}H-NMR spectrum of substrate 5u

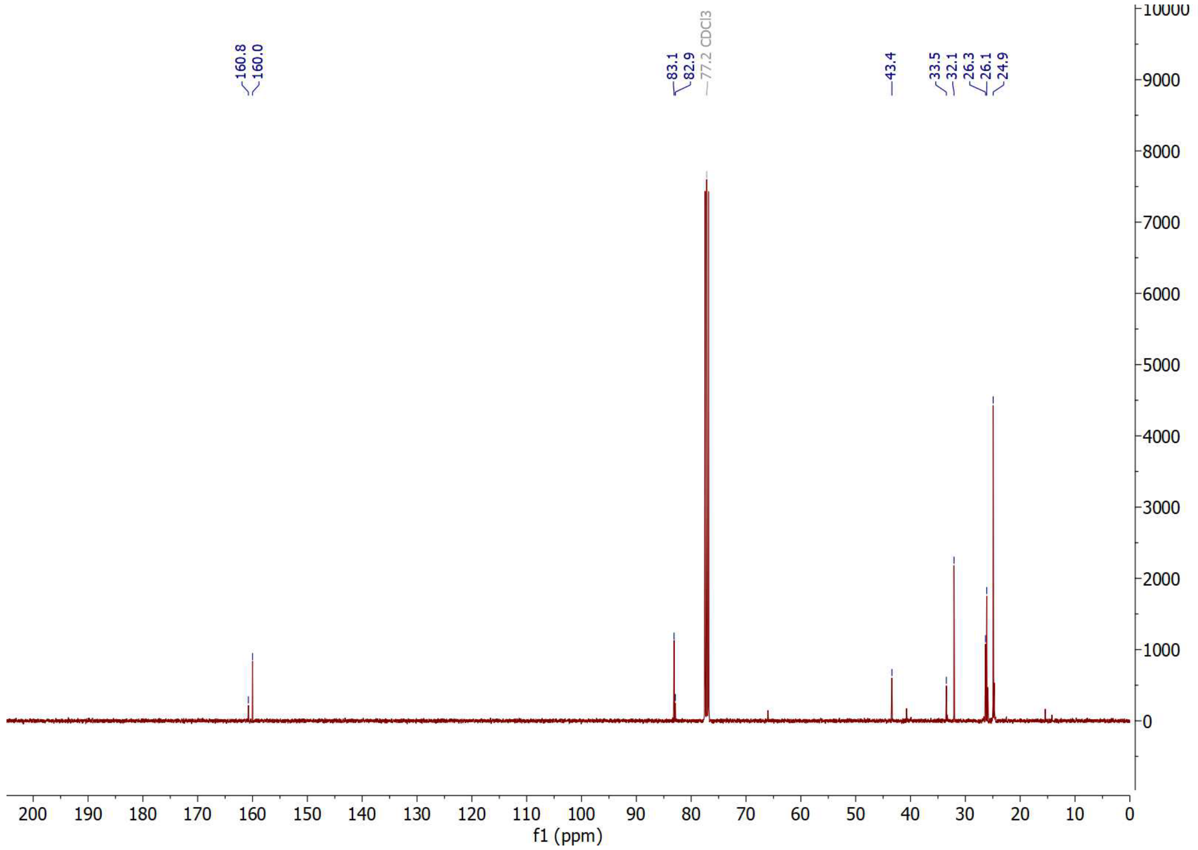

Figure S67. ^{13}C{^{1}H}-NMR spectrum of substrate 5u

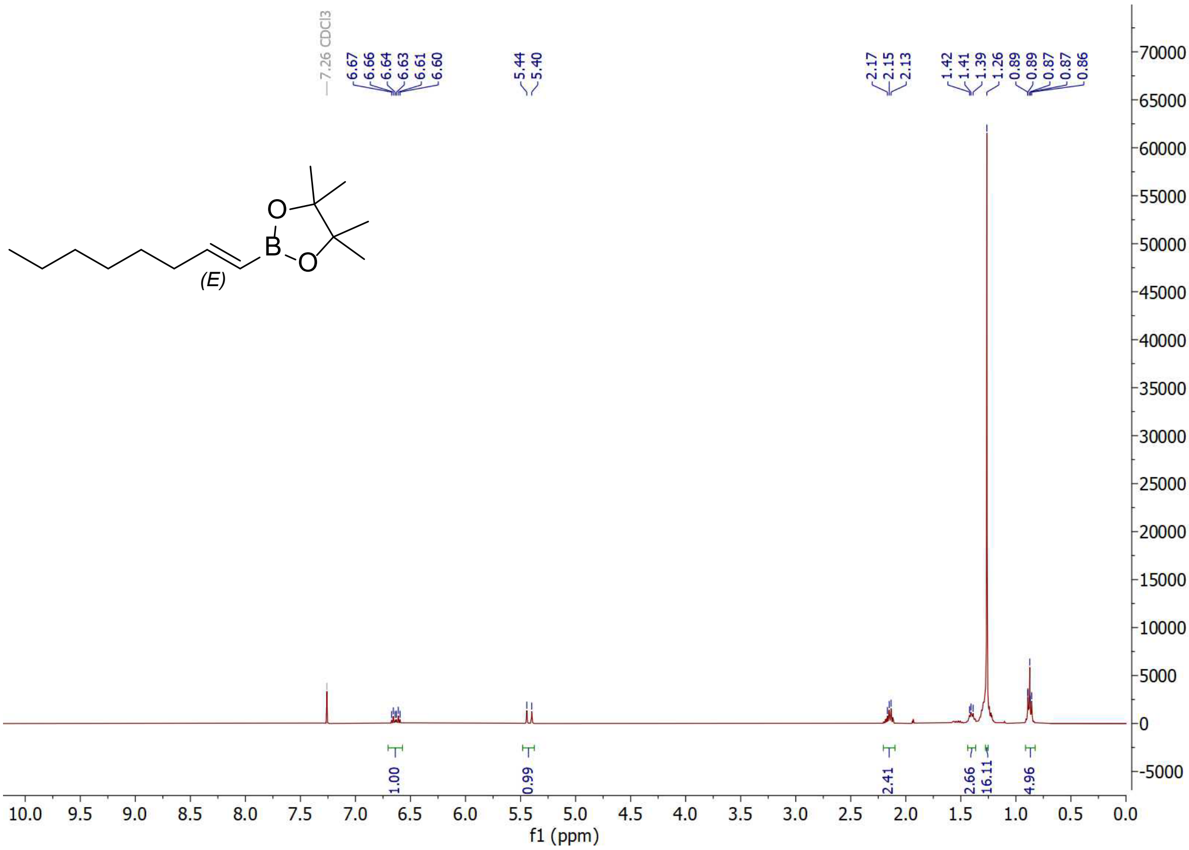

Figure S68. ¹H-NMR spectrum of substrate 5v

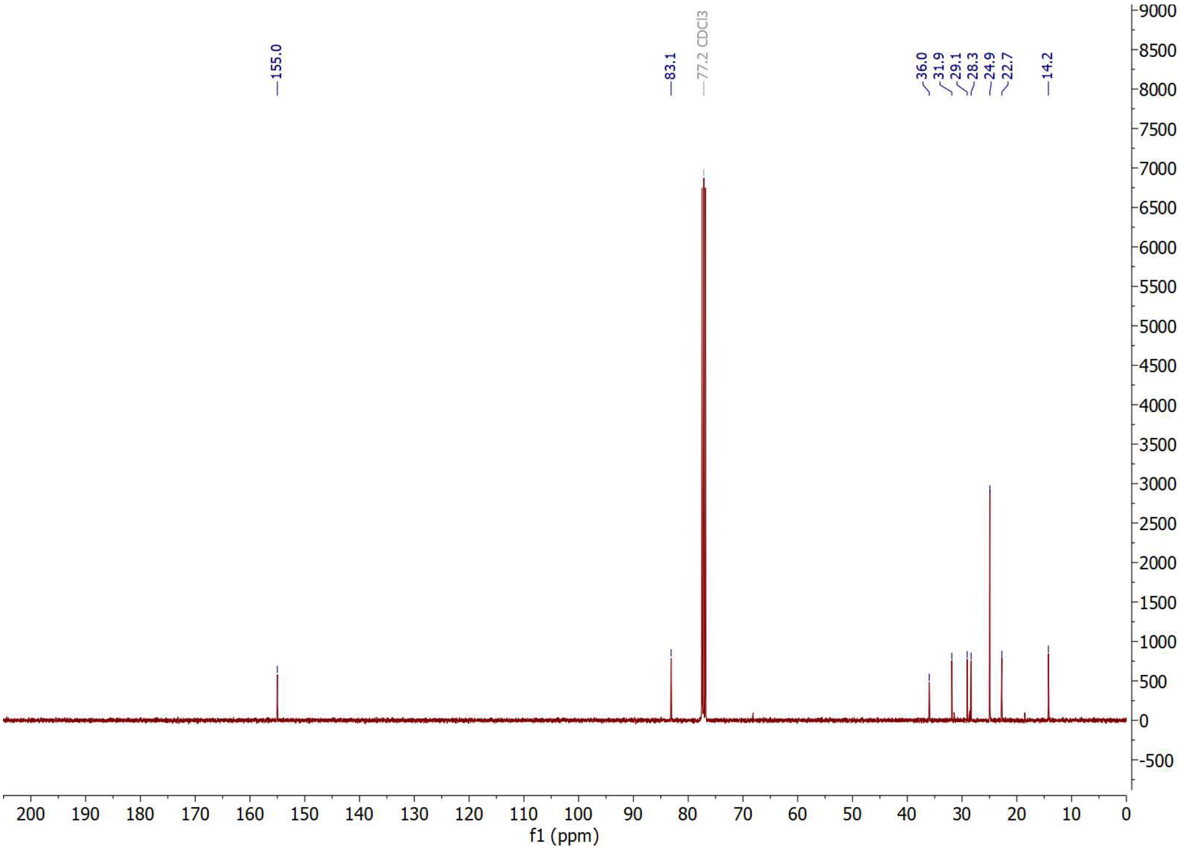

Figure S69. ¹³C{¹H}-NMR spectrum of substrate 5v

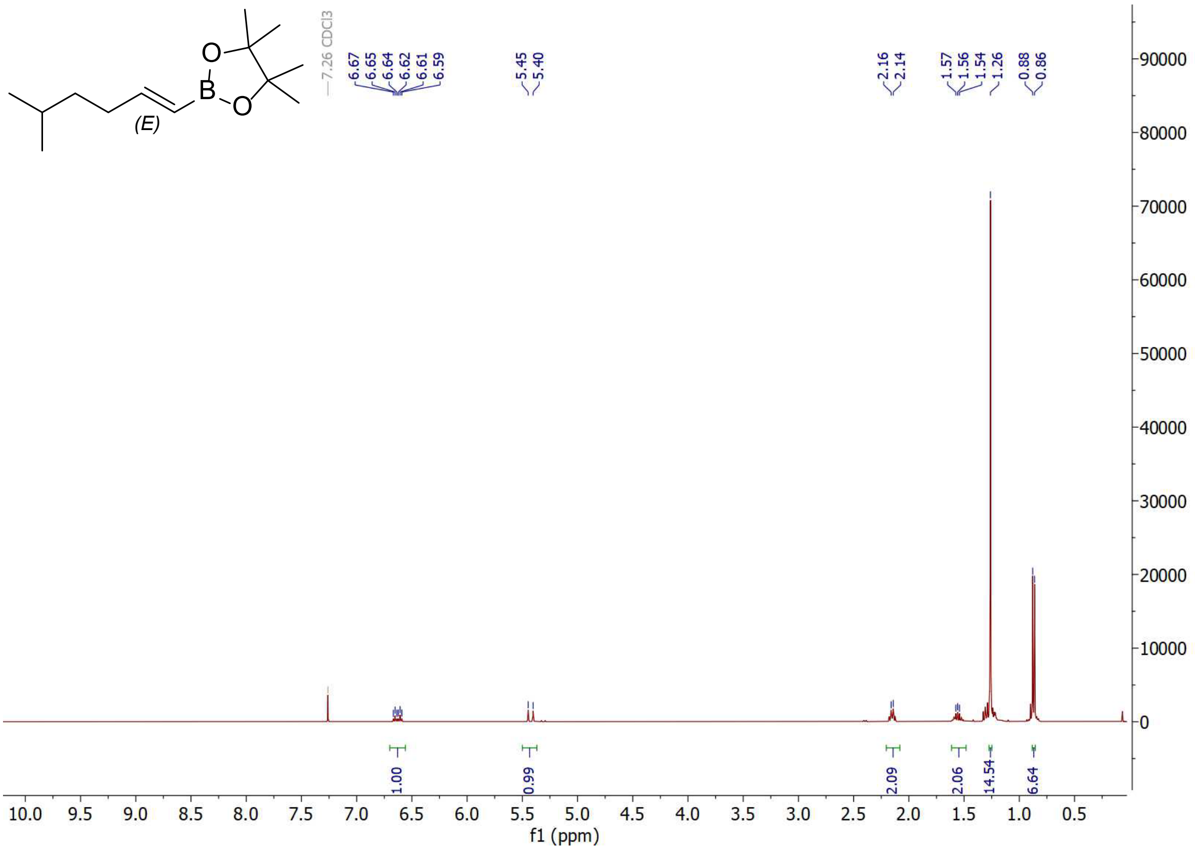

Figure S70. ^{1}H-NMR spectrum of substrate 5w

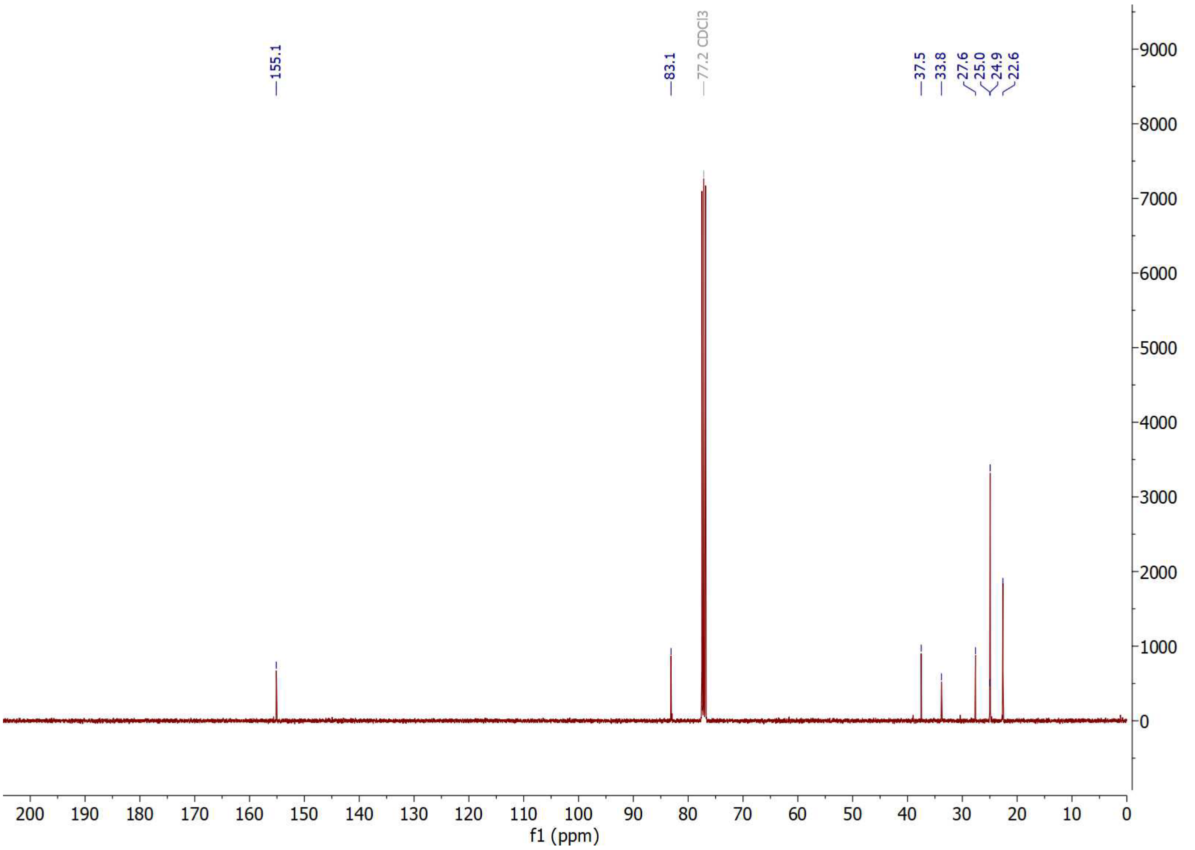

Figure S71. ^{13}C{^{1}H}-NMR spectrum of substrate 5w

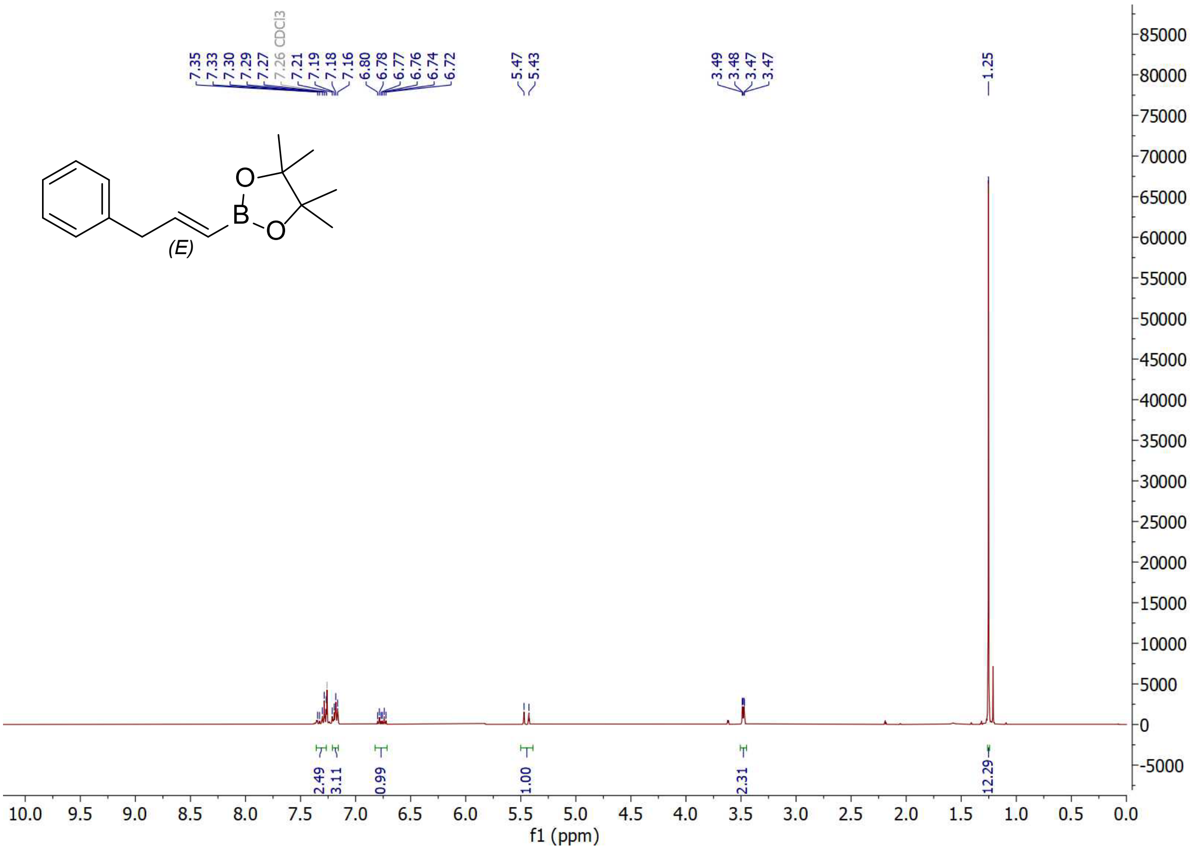

Figure S72. ^{1}H-NMR spectrum of substrate 5x

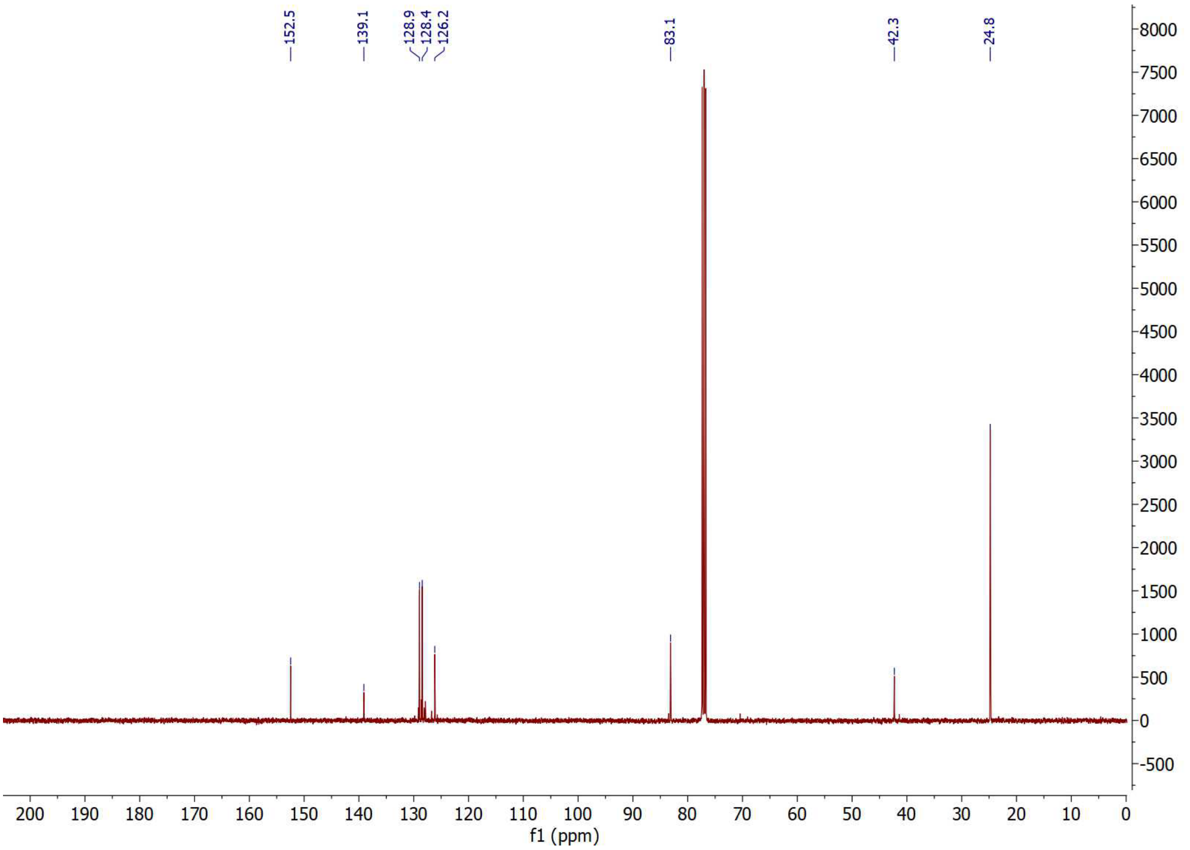

Figure S73. $^{13}C\{^{1}H\}$-NMR spectrum of substrate 5x
